# Evolution-guided protein design of IscB for persistent epigenome editing in vivo

**DOI:** 10.1038/s41587-025-02655-3

**Published:** 2025-05-07

**Authors:** Soumya Kannan, Han Altae-Tran, Shiyou Zhu, Peiyu Xu, Daniel Strebinger, Rachel Oshiro, Guilhem Faure, Lukas Moeller, Julie Pham, Kepler S. Mears, Heyuan M. Ni, Rhiannon K. Macrae, Feng Zhang

**Affiliations:** 1 Howard Hughes Medical Institute, Cambridge, MA USA; 2 Broad Institute of MIT and Harvard, Cambridge, MA USA; 3 McGovern Institute for Brain Research, Massachusetts Institute of Technology, Cambridge, MA USA; 4 Department of Brain and Cognitive Science, Massachusetts Institute of Technology, Cambridge, MA USA; 5 Department of Biological Engineering, Massachusetts Institute of Technology, Cambridge, MA USA; 6 Yang Tan Collective, Cambridge, MA USA

**Keywords:** Molecular engineering, Molecular biology

## Abstract

Naturally existing enzymes have been adapted for a variety of molecular technologies, with enhancements or modifications to the enzymes introduced to improve the desired function; however, it is difficult to engineer variants with enhanced activity while maintaining specificity. Here we engineer the compact Obligate Mobile Element Guided Activity (OMEGA) RNA-guided endonuclease IscB and its guiding RNA (ωRNA) by combining ortholog screening, structure-guided protein domain design and RNA engineering, and deep learning-based structure prediction to generate an improved variant, NovaIscB. We show that the compact NovaIscB achieves up to 40% indel activity (~100-fold improvement over wild-type OgeuIscB) on the human genome with improved specificity relative to existing IscBs. We further show that NovaIscB can be fused with a methyltransferase to create a programmable transcriptional repressor, OMEGAoff, that is compact enough to be packaged in a single adeno-associated virus vector for persistent in vivo gene repression. This study highlights the power of combining natural diversity with protein engineering to design enhanced enzymes for molecular biology applications.

## Abstract (web summary)

A combination of engineering approaches is used to improve the function of IscB for a variety of editing activities in vivo.

## Main

Natural programmable enzymes have transformed our ability to study and treat complex diseases. The application and engineering of RNA-guided systems, most notably clustered regularly interspaced short palindromic repeats (CRISPR)–Cas9, has accelerated genetic screening methods to discover the genetic basis of diseases, enabled the generation of precision cellular and animal disease models, and given rise to novel therapeutics to treat genetic diseases in the clinic^1^.

Expanding the therapeutic application of reprogrammable genome editors necessitates the development of highly efficient and specific systems that can be delivered safely and precisely to targeted cell types. Since the initial deployment of *Streptococcus pyogenes* CRISPR–Cas9 (SpCas9) for mammalian genome editing, genome mining^2–7^, rational mutagenesis and structure-guided protein design^8,9^, and directed evolution approaches^10,11^ have resulted in new systems with improved activity, specificity and size. However, balancing enhanced activity while maintaining specificity remains an outstanding challenge^12^ and there is also a critical need for genome editing tools that are compact to facilitate in vivo delivery, particularly for tools that incorporate other functional domains, such as transcriptional activators and repressors, resulting in large constructs that are challenging to deliver^13^.

**Fig. 1:**
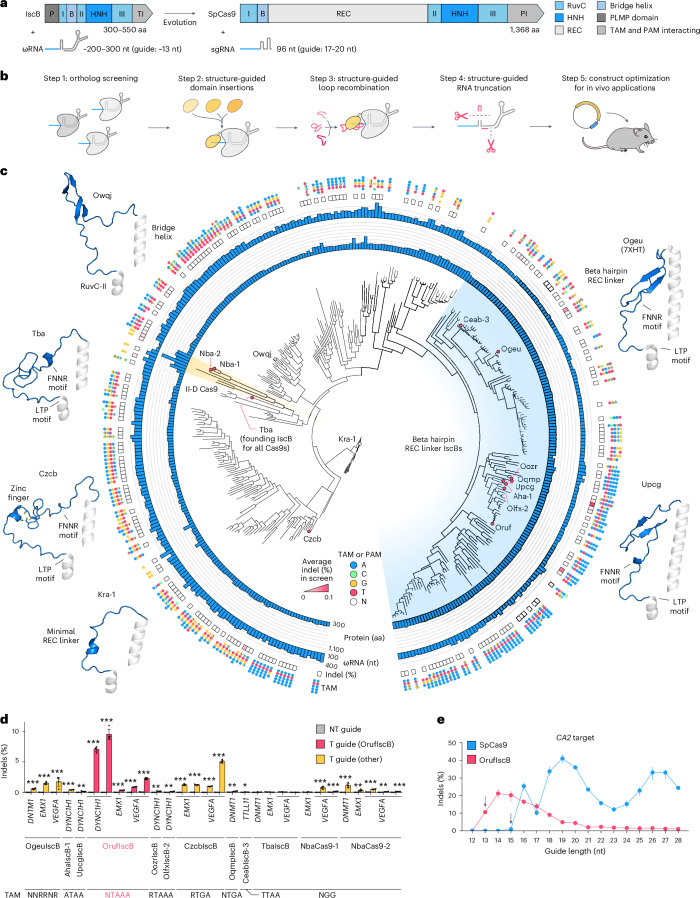
Overall evolution-guided engineering strategy and identification of OrufIscB as an active human genome editor. **a**, Comparison of IscB and Cas9 protein size, domain architecture and ncRNAs. P, PLMP domain; B, bridge helix domain; I, RuvC domain region I; II, RuvC domain region II; III, RuvC domain region III; TI, TAM-interacting domain; PI, PAM-interacting domain; ncRNA, noncoding RNA. **b**, Schematic overview of the overall discovery and engineering pipeline used to develop an IscB suitable for in vivo mammalian applications. **c**, Phylogenetic tree of all experimentally characterized IscBs and select type II-D Cas9s. Protein and associated ωRNA length, average indel activity across 12 targeted sites in the human genome in a pooled-guide assay, and dominant TAM preference are shown on the outer rings. The circled tree nodes indicate specific orthologs; red filled circles indicate the ortholog had genome editing activity in human cells. Example AlphaFold2 structures of REC linkers and mini-RECs of select orthologs are shown on the left and right, with the solved cryo-EM structure used for OgeuIscB (PDB: 7XHT (ref. ^16^)). **d**, Validation of the 12-guide pooled screen using individual guides. Indels are shown only for those combinations of protein and guide that showed activity in the pooled screen. T, targeting; NT, nontargeting control. Data are presented as mean ± s.d.; *n* = 4 replicate transfections. *P < 0.05; **P < 0.01; ***P < 0.001 from two-sided *t*-test relative to the NT guide. The *P* values are included in Supplementary Table 2. **e**, Indel formation at *CA2* mediated by OrufIscB or SpCas9 using guide lengths ranging from 12 to 28 nt. The black arrows show the shortest lengths with detectable activity. Data are presented as mean ± s.d.; *n* = 3 replicate transfections.

The recently discovered Obligate Mobile Element Guided Activity (OMEGA) systems are a distinct class of miniature RNA-guided nucleases capable of targeted genome editing in human cells^14,15^. OMEGA–IscB, the ancestor of Cas9^14^, is a particularly compelling candidate for genome and epigenome editing due to its small size (~300–550 amino acids (aa)), which renders it more conducive to delivery via adeno-associated viruses (AAVs); its dual nuclease composition, which enables control over which DNA strands are cleaved; and its large-structured guiding RNA (ωRNA), which offers an interface to scaffold additional interactions (Fig. 1a). In addition, the open and accessible R-loop formed by OMEGA–IscB binding to double-stranded DNA (dsDNA)^16–18^ enables double-strand-break-free applications, such as base editing^19^. However, IscBs may have low specificity, owing to their short effective guide lengths (~13 bp), in contrast to SpCas9, which uses a longer guide (~17–20 bp) (Fig. 1a)^14,20^.

Recent efforts have focused on engineering OMEGA effectors for increased editing efficiency in human cells^21–26^. However, due to the complex relationship between on-target activity and specificity, focusing predominantly on optimizing on-target activity may exacerbate the low specificity shown by these systems, potentially rendering the engineered variants unsuitable for in vivo and therapeutic applications^12^. Advances in protein design, genome mining and protein structure prediction^27–29^ offer promising new methods to simultaneously optimize the activity and specificity of IscB. Here we combine large-scale ortholog screening, evolution-guided protein design and rational RNA engineering to dramatically alter the activity and function of IscB as a genome editor (Fig. 1b). We subsequently engineer IscB fusion systems, including base editors and epigenome editors, and show that an IscB-based epigenome editor, OMEGAoff, can confer long-lasting epigenome edits with phenotypic outcomes in vivo using single-vector AAV delivery.

## Results

### Screening natural IscB variants for efficient genome editors

**Extended Data Fig. 1:**
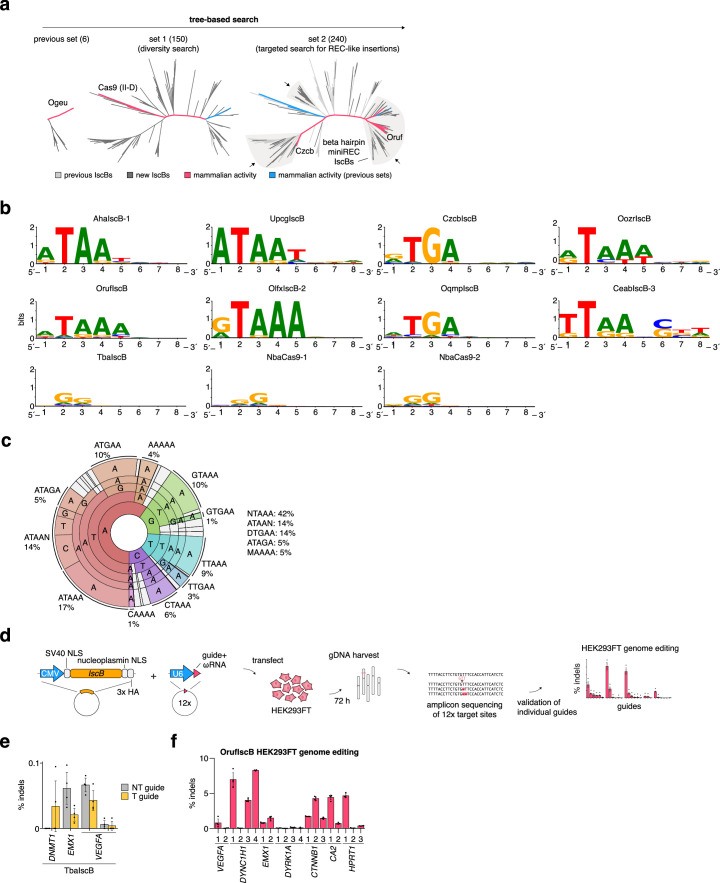
Ortholog screening of IscB family, related to Fig. 1. (**A**) Experimental traversal of the IscB phylogenetic tree to identify IscB orthologs active for human genome editing. Initial set of 6 tested IscBs shown on left. Set 1 of phylogenetically diverse IscBs/II-D Cas9s shown in middle. Set 2 of mainly larger IscBs with potential REC inserts shown on right. (**B**) TAMs of active IscB proteins determined by in vitro cleavage assays. All the protein sequences and accession of source contigs are listed in Table S1. (**C**) TAM wheel representation of OrufIscB TAM. All motifs adding up to 5% or more of cleaved TAMs are listed to the right. Constituents of each relevant motif are labeled around the circumference of the TAM wheel. (**D**) Schematic of the ortholog screening in HEK293FT cells (results shown in Fig. 1C). (**E**) Zoomed in y-axis representation of Indel formation mediated by TbaIscB with individual guides in HEK293FT cells displayed in Fig. 1D. Data are presented as mean +/- SD, n = 4 replicate transfections. (**F**) Indel formation mediated by WT OrufIscB with individual guides in HEK293FT cells. Data are presented as mean +/- SD, n = 4 replicate transfections.

**Extended Data Fig. 2:**
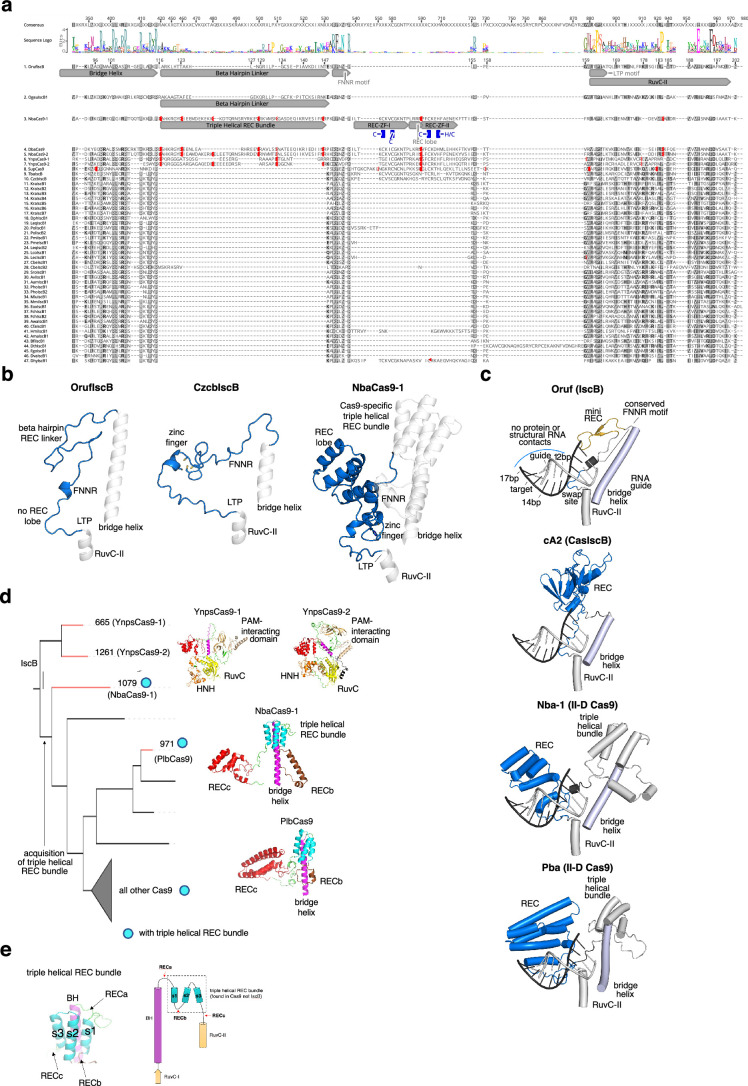
Sequence and structure comparison of IscBs and II-D Cas9s, related to Fig. 1, and evolution of the Cas9 REC domain triple helical bundle, related to Fig. 2. (**A**) Alignment of tested IscBs and early II-D Cas9s in the location between the bridge helix (BH) and RuvC-II regions using MAFFT. The consensus sequence is at the top, followed by the sequence logo. OrufIscB is set as the reference sequence for comparison. The conserved FNNR and LTP motifs flanking REC insertions are shown. Hidden insertions (from less than 2% of loci) are indicated by red jagged lines. (**B**) Comparison of AlphaFold2 models of OrufIscB, CzcbIscB, and NbaCas9-1 focusing on the REC insertion region between the bridge helix and RuvC-II. The conserved FNNR and LTP sites are shown. The conserved zinc finger is also shown along with the REC lobe of NbaCas9-1. OrufIscB contains a unique beta hairpin REC linker between the FNNR motif and bridge helix. (**C**) AlphaFold2 models of the REC domain and surrounding regions from two IscBs and two Type II-D Cas9s were superimposed onto the OgeuIscB structure (PDB: 7XHT ref. ^16^), using the RuvC-II and bridge helix regions as templates for alignment. The guide:target duplex from OgeuIscB is shown in the final model, but the OgeuIscB protein itself is not shown. (**D**) Left: Zoom in to the previously described phylogenetic tree^14^ focusing on early Cas9 evolution from IscB. Cluster names are shown along the tree. Right: AlphaFold2 models of the cluster representatives. Early II-D Cas9s contain RECc insertions and RECb insertions into the triple helical REC bundle that emerged quickly after IscB evolved into Cas9. (**E**) Architecture of the triple helical REC bundle. Top: structure model of a triple helical REC bundle. Bottom: schematic of the triple helical rec bundle. In both cases, arrows point to where 3 different classes of REC domains insert into (RECa, RECb, and RECc).

We previously tested six IscB orthologs for activity in human cells and identified one system, OgeuIscB, capable of mammalian genome editing (Extended Data Fig. 1a)^14^. However, OgeuIscB has an optimal guide length of 16 nucleotides (nt), which poses a challenge for sequence-specific targeting in the human genome. As a first step toward an improved OMEGA effector, we hypothesized that testing additional diverse IscBs may enable us to find an ortholog with higher baseline activity when paired with longer guides (20 nt). Sampling diversely across protein size, ωRNA scaffold type and taxonomic distribution, we first curated a set of 144 IscBs and 6 type II-D Cas9s (the most closely related Cas9 homologs to IscB, which we reasoned may provide insight into which protein and RNA features are necessary for mammalian genome editing activity) and tested them for in vitro function and target adjacent motif (TAM) preference using an in vitro transcription–translation (IVTT) TAM screen (Extended Data Fig. 1b,c and Supplementary Table 1). Orthologs showing activity in vitro were then tested for function in human cells using a pool of 12 guides of 20 nt length per ortholog (Extended Data Fig. 1d)^14^. We found that only the previously identified OgeuIscB, TbaIscB and two type II-D Cas9s showed potential genome editing activity in human cells using the pool of 12 guides (Fig. 1c and Extended Data Fig. 1a). These four proteins shared a common feature of diverse recognition lobe (REC)-like inserts located between the bridge helix (BH) and RuvC-II regions, such as the beta hairpin REC linker found in OgeuIscB (Fig. 1c and Extended Data Fig. 2a). This prompted us to perform a second, more targeted search for larger IscBs that may contain REC-like insertions (Extended Data Fig. 1a). Accordingly, we selected a second set of 240 IscBs, tested them as before—both in vitro and in human cells—and identified 8 additional IscBs that showed potential mammalian genome editing activity from this screening pipeline (Fig. 1c and Extended Data Fig. 1a). Thus, from our screens, we identified in total 10 IscBs and 2 type II-D Cas9s possessing potential genome editing activity in human cells in addition to OgeuIscB.

We then validated the activity of these proteins using individual guides and found that 10 IscBs, including OgeuIscB, along with the 2 type II-D Cas9s, NbaCas9-1 and NbaCas9-2, were active for human genome editing (Fig. 1d and Extended Data Fig. 1e). Of these, OrufIscB, which is 492 aa long, had the highest insertion–deletion (indel) rates relative to the other identified orthologs in both the initial pooled 12-guide screen and validation assays (Fig. 1d). OrufIscB recognizes an NTAAA TAM along with noncanonical TAMs consisting of ATAAA paired with a single mismatch from the distribution NWRRN (Extended Data Fig. 1b,c). To evaluate the robustness and range of the genome editing activity of OrufIscB, we tested OrufIscB with a panel of 20 guides adjacent to the most optimal ATAAA TAM, including 18 additional target sites, and found that it showed detectable indels at 14 out of 20 sites, with efficiencies ranging from 0.2% to 8% (Extended Data Fig. 1f and Supplementary Table 2), a five- to tenfold improvement over the previously reported activity of OgeuIscB^14^.

To further characterize OrufIscB, we defined the effective guide length as the minimum number of matched bases in the guide:target duplex required by the protein for cleavage activity. Effective guide length is a major contributor to specificity, as shorter effective guides have more potential off-targets across the human genome (for a given target sequence and fixed TAM or protospacer adjacent motif (PAM), a 12-nt guide with perfect fidelity would have on average ~4,100 more off-targets compared with an 18-nt guide with perfect fidelity). Moreover, simply extending the guide length beyond the effective guide length can result in worse specificity by further stabilizing mismatched duplexes, as was shown for SpCas9 (refs. ^30,31^). Therefore, systems with higher effective guide lengths are preferable for site-specific genome editing because they check more positions for match fidelity. To evaluate the effective guide length of OrufIscB, we tested OrufIscB with guide lengths ranging from 12 nt to 28 nt and observed optimal efficiency with 14–15-nt guides (Fig. 1e), with detectable activity using guides as short as 13 nt. This stands in contrast to SpCas9, which optimally uses 18–20-nt guides and does not show activity with guides shorter than 16 nt (Fig. 1e)^30^. The lower effective guide length of IscB is further supported by the lack of cryo-electron microscopy (cryo-EM) density of both protein and guide:target duplex in the TAM-distal region past position 13 in OgeuIscB^16,17^. We therefore reasoned that increasing the relatively low effective guide length of IscB could result in an OrufIscB variant with improved specificity.

To this end, we sought to simultaneously increase both the editing activity and effective guide length of OrufIscB through protein engineering. Eight of the IscB proteins with human genome editing activity belong to the same clade and are exclusively found in human gut-related metagenomes, which may partially explain why this clade is well adapted for genome editing in human physiological contexts. The IscBs in this clade (beta hairpin REC linker IscBs) contain a distinct, conserved beta hairpin REC linker that interacts with the guide:target duplex, potentially contributing to an extended guide:target duplex^14,16,17^ (Fig. 1c). We previously hypothesized that REC domains may facilitate DNA unwinding in the complex landscape of eukaryotic chromatin, analogous to the function of the larger REC lobe in Cas9 (ref. ^32^), which may also contribute to the increased rates of mammalian genome editing activity in this clade. CzcbIscB, which does not belong to this clade, also contains a small REC-like zinc finger that is homologous to and potentially an evolutionary precursor of the zinc finger found in larger type II-D Cas9 REC domains (Fig. 1c and Extended Data Fig. 2b), further supporting the hypothesis that an REC domain is important for editing activity of eukaryotic genomes. Consequently, we chose to focus on REC domain insertions and engineering for subsequent efforts.

### Evolution-guided redesign of OrufIscB for efficient editing

**Fig. 2:**
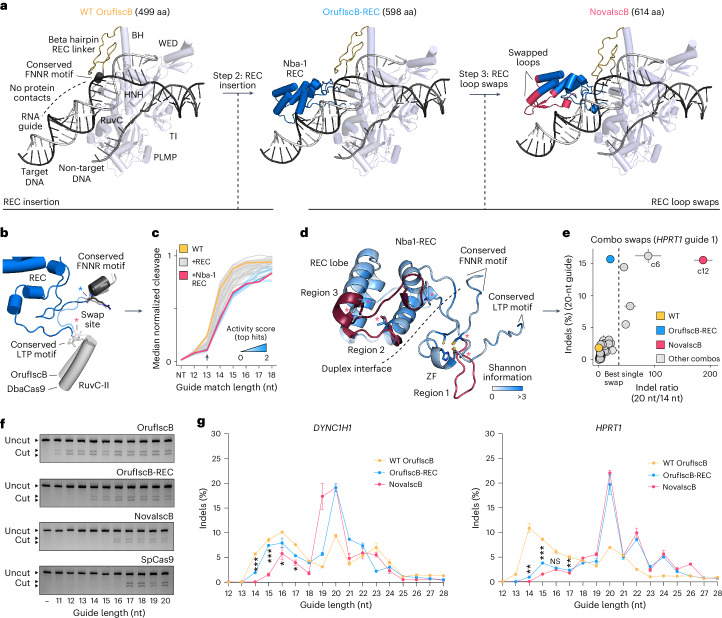
Evolution-guided protein engineering of OrufIscB. **a**, Stepwise evolution-guided engineering of OrufIscB. The AlphaFold2 structures of WT OrufIscB, OrufIscB-REC and NovaIscB are shown. **b**, AlphaFold2 model of the REC domain insertion site in OrufIscB overlaid with a homologous site in DbaCas9. **c**, In vitro cleavage activity of selected 55 REC domain grafts in OgeuIscB with guides with progressively increasing target-matching sequence. **d**, AlphaFold2 model of the REC region of OrufIscB-REC with swapped loops from AlphaFold2 models of other REC domains labeled in three shades of red corresponding to regions 1, 2 and 3. The AlphaFold2 model of the swap 49 loop is shown for region 1. Conserved amino acids flanking each swap region are depicted as side chains. Sequence alignment entropy is mapped onto the structure as a white-to-blue gradient, with entropies of up to 3 being shown as blue. ZF, zinc finger. Each asterisk denotes a swap site. **e**, Indel formation mediated by OrufIscB-REC harboring 54 double loop swaps in the REC domain with either a 20-nt ωRNA or a 14-nt ωRNA targeting *HPRT1*. The indel ratio (20 nt/14 nt) and indel activity with the 20-nt guide for each variant are plotted. Combination 12 (c12, red) was selected as NovaIscB. The dashed vertical line indicates the best single-swap indel ratio. Data are presented as mean ± s.d.; *n* = 3 replicate transfections. **f**, In vitro cleavage assay with WT OrufIscB, OrufIscB-REC, NovaIscB and SpCas9 using ωRNAs or guideRNAs (gRNAs) 11 nt to 20 nt long. The experiments were repeated two times independently. **g**, Indel formation mediated by WT OrufIscB, OrufIscB-REC and NovaIscB using guide lengths ranging from 12 nt to 28 nt at two target sites. Data are presented as mean ± s.d.; *n* = 3 replicate transfections. *P < 0.05; **P < 0.01; ***P < 0.001 from the two-sided *t*-test for OrufIscB-REC versus NovaIscB. NS, not significant. For the *DYNC1H1* guide, the *P* values are 0.00002 (14 nt), 0.00005 (15 nt), 0.02642 (16 nt) and 0.01150 (17 nt). For the *HPRT1* guide, the *P* values are 0.00153 (14 nt), 0.00024 (15 nt), 0.06375 (16 nt) and 0.00130 (17 nt). Source data

We set out to redesign OrufIscB via analysis of the structural evolution of IscBs and Cas9s (Fig. 2a–e). Using AlphaFold2 (ref. ^27^) modeling and superposition onto cryo-EM structures of the OgeuIscB ternary complex^16,17^, we observed that minimal protein and RNA contacts are made with the guide RNA:target DNA duplex in OrufIscB beyond 14 bp from the TAM (Extended Data Fig. 2c). However, in some IscBs and most Cas9s, the REC domain constitutes an extensive insertion that is predicted to contact the TAM-distal region of the RNA:DNA heteroduplex and recognize target complementarity with the guide (Extended Data Fig. 2c)^30,33^. We reasoned that increasing the surface area of protein in contact with the guide:target heteroduplex could increase the effective guide length. Accordingly, for our second engineering step, we hypothesized that inserting REC and REC-like domain segments into wild-type (WT) OrufIscB could enhance recognition of the exposed heteroduplex region and increase the effective guide length while performing mismatch detection (Fig. 2a).

**Extended Data Fig. 3:**
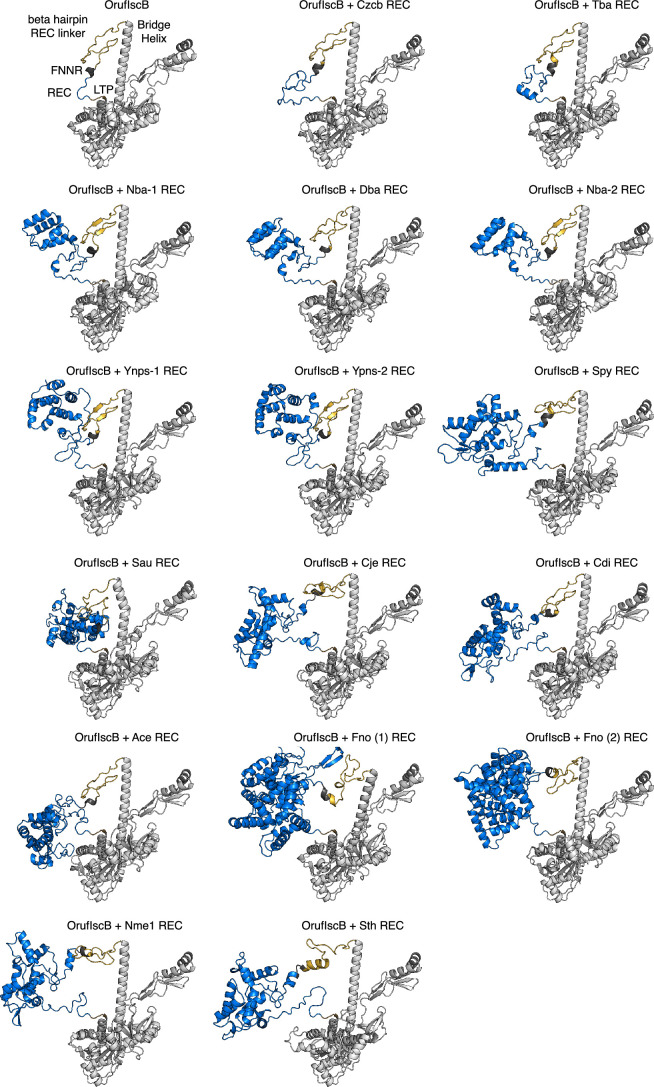
In silico testing of correct folding of OrufIscB REC grafts, related to Fig. 2. AlphaFold2 models of REC domains grafted into OrufIscB. REC domains are colored in blue. The FNNR motif is colored in black, while the LTP motif is colored in brown. The beta hairpin REC linker is colored in orange. The bridge helix is additionally shown, but not colored. OrufIscB + Nba-1 REC, for example, maintains correct folding of the FNNR motif, the beta hairpin REC linker, and the corresponding REC lobe, as well as the REC zinc finger.

To determine where to insert REC domains in the OrufIscB scaffold, we used AlphaFold2 to model the REC and REC-like insertion sites in multiple distinct IscBs and Cas9s. Previous observations have suggested that the REC domains of Cas9s are generally not conserved^32^. However, our own analysis of Cas9 early evolution showed that Cas9s have a conserved triple helical REC bundle that scaffolds additional diverse, weakly conserved insertions in the REC domain (Extended Data Fig. 2d,e), indicating conserved attachment points to the BH. Furthermore, we found that the small REC domains in some type II-D Cas9s share short (5–7 aa) flanking motifs that are conserved at both the structure and sequence levels (Fig. 1c and Extended Data Fig. 2a,c). These include a conserved positively charged RNA-contacting FNNR motif (named after the consensus sequence EARFNNR) and a conserved helical RuvC-II LTP motif (named after the consensus sequence LTPSA) (Figs. 1c and 2b). These conserved flanking residues are also shared by TbaIscB, the founding IscB member of the Cas9 lineage^14^, CzcbIscB, OrufIscB, OgeuIscB and other beta hairpin REC linker IscBs (Fig. 1c and Extended Data Fig. 2a,b). Given the homology surrounding REC domains, we reasoned that REC domains from most type II-D and some type II-A, B and C Cas9s could be inserted into the homologous location in OrufIscB (Fig. 2b). We modeled various REC insertions grafted into OrufIscB using AlphaFold2 and found that most REC insertions, including those from SpCas9 and type II-D Cas9s, maintained apparently correct folding of the REC domain and the rest of the IscB protein without disrupting the beta hairpin REC linker (Extended Data Fig. 3 and Supplementary Table 3).

**Extended Data Fig. 4:**
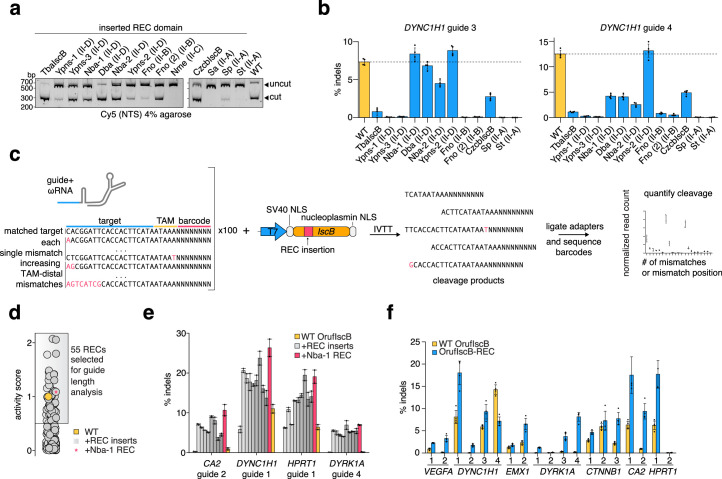
Experimental validation and screening of REC grafts into OrufIscB, related to Fig. 2. (**A**) IVTT cleavage assay of OrufIscB (WT) with various REC insertions using IVTT protein and WT ωRNA with labeled target (target strand: Cy3; non-target strand: Cy5). Gel was imaged in Cy5 channel only. WT: Wildtype, NTS: non-target strand. (**B**) Indel formation mediated by WT OrufIscB with various REC insertions for two different guides in HEK293FT cells. Data are presented as mean +/- SD, n = 4 replicate transfections. (**C**) Schematic of the IVTT REC screening assay that tests for cleavage with varying levels of TAM distal mismatches. (**D**) In vitro cleavage activity score of all the screened REC domains in IVTT REC screening assay. (**E**) Indel formation mediated by WT OrufIscB and variants with various REC domain insertions in HEK293FT cells. Data are presented as mean +/- SD, n = 2 replicate transfections. (**F**) Indel formation mediated by OrufIscB (WT) vs OrufIscB-REC (OrufIscB + Nba-1 REC) across a larger panel of guides in HEK293FT cells. Data are presented as mean +/- SD, n = 4 replicate transfections. Source data

On the basis of the success of folding in silico REC insertions in OrufIscB, we constructed 14 OrufIscB-REC chimeras and found that 12 of them retained DNA cleavage activity in vitro (Extended Data Fig. 4a). We tested these chimeric proteins at 2 target sites in the human genome using 20-nt guides and found that the REC domain chimeras from the type II-D Cas9s (Nba-1, Dba, Nba-2 and Ypns-2) and from CzcbIscB retained appreciable activity in human cells (Extended Data Fig. 4b). This result may be partially explained by the accurate apparent folding (in silico) of the beta hairpin REC linker for insertions originating from type II-D Cas9s and CzcbIscB (Extended Data Fig. 3). Given these positive preliminary results, we constructed a larger set of 183 OrufIscB-REC domain chimeras derived mainly from early type II-D Cas9s, which appeared to be the most compatible with OrufIscB from our initial experiments (Supplementary Table 3). To assess the ability of these REC domain insertions to increase effective guide length and retain high dsDNA cleavage efficiency, we tested each variant using an IVTT target and guide screen with a library of 100 ωRNAs with 20-nt guides targeting human genomic sequences and a corresponding library of targets including every possible mismatch and increasing numbers of TAM-distal mismatches for each of the 100 targets to assess the effective guide length (Extended Data Fig. 4c and Supplementary Table 3). From this screen, we identified 55 REC insertion variants that showed appreciable activity relative to the WT (Extended Data Fig. 4d). Of these 55, 43 showed decreased cleavage with 5-, 6- and 7-nt TAM-distal mismatches (effective guide lengths of 15, 14 and 13 nt, respectively) relative to WT OrufIscB (Fig. 2c). From these, we selected 10 top candidates for further validation in human cells. We found that several improved the on-target activity of OrufIscB in human cells, with the variant containing the REC domain from Nba-1, which we termed OrufIscB-REC, showing the best improvement on average across 4 tested target sites (Extended Data Fig. 4e). We further compared the activity of WT OrufIscB and OrufIscB-REC and found that the chimeric protein improved activity up to 20-fold for some guides and showed activity at several sites where no indels were generated by the WT protein (Extended Data Fig. 4f).

We assessed the effect of guide length on the activity of OrufIscB-REC both in vitro and at two target sites in the human genome. We found that the chimeric version showed an increase in the minimum guide length that supports activity in vitro relative to WT OrufIscB (from 11 to 14 nt) (Fig. 2f), as well as a pronounced increase in activity with ~19–20-nt guides and a simultaneous decrease in activity with 13–17-nt guides in HEK293FT cells (Fig. 2g and Supplementary Table 2).

**Extended Data Fig. 5:**
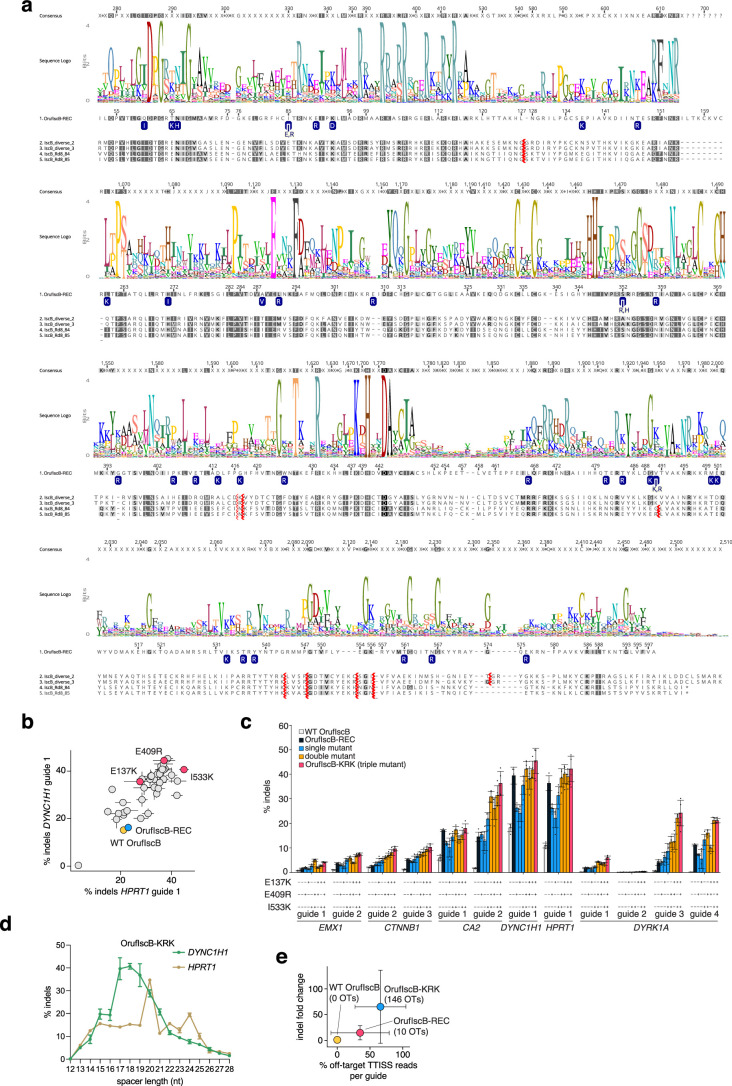
Point mutation selection and testing for increasing OrufIscB-REC activity, related to Fig. 2. (**A**) Multiple sequence alignment and weblogo of all tested IscBs and early Cas9 (II-D) against a reference sequence of OrufIscB-REC, including the proposed point mutations for increasing activity of IscB. Most proposed point mutations were selected from the natural amino acid distribution at each position, prioritizing mutations that may improve contacts with the DNA or improve packing of the protein. The first 4 non-OrufIscB-REC sequences are shown as examples in the figure, but the multiple sequence alignment used for this analysis contains all sequences. (**B**) Indel formation mediated by OrufIscB-REC with each point mutation across 2 target sites. Data are presented as mean +/- SD, n = 4 replicate transfections. (**C**) Indel formation mediated by OrufIscB-REC with single, double, and triple mutation combinations using the top 3 mutations from distant parts of the protein that were hypothesized to combine synergistically across a panel of 12 guides. Data are presented as mean +/- SD, n = 4 replicate transfections. (**D**) Indel formation mediated by OrufIscB-KRK using guide lengths ranging from 12 to 28 nt at two target sites. Data are presented as mean +/- SD, n = 3. (**E**) Specificity analysis based on the average indel fold changes of OrufIscB-REC or OrufIscB-KRK vs WT OrufIscB and % off-target reads in total from tagmentation-based tag insertion site sequencing (TTISS) using a pool of 20 nt guides with WT OrufIscB, OrufIscB-REC, and OrufIscB-KRK.

We next sought to improve the efficiency of OrufIscB-REC via rational mutagenesis. We selected naturally occurring amino acids across diverse IscBs at positions that probably contact nucleic acids or show poor hydrophobic packing based on the AlphaFold2 model of OrufIscB and introduced 37 mutations into OrufIscB-REC (Extended Data Fig. 5a). We found that most variants improved activity by 1.5–2× relative to OrufIscB-REC across 2 tested guides (Extended Data Fig. 5b). From these, we selected 3 mutants at different locations in the predicted structure of OrufIscB (E137K, E409R, I533K) and tested all possible combinations of them, including single, double and triple mutants. We found that the triple mutant, which we termed OrufIscB-KRK after the three amino acid changes, showed the best improvement in activity relative to OrufIscB-REC (Extended Data Fig. 5c). We further evaluated the effect of guide length on the activity of OrufIscB-KRK and found that OrufIscB-KRK significantly boosted the activity on short guides down to 13 nt, raising the possibility that this hyperactive variant may generate increased off-targets in the genome (Extended Data Fig. 5d). To assess this, we compared genome-wide off-target cleavage induced by WT OrufIscB, OrufIscB-REC and OrufIscB-KRK with 12 guides using tagmentation-based tag insertion site sequencing (TTISS)^12^. We found that although the Nba-1 REC insertion into OrufIscB improved the on-target activity while minimally compromising specificity, we observed a sharp drop in specificity when the three mutations were introduced (Extended Data Fig. 5e and Supplementary Table 5), suggesting that point mutations generally alter overall binding affinity to DNA without substantially altering differential binding to on-target versus off-target DNA.

### Optimizing REC domain duplex recognition for NovaIscB

To achieve a higher-specificity variant, in our third engineering step, we sought to optimize OrufIscB-REC by modifying multiple residues in flexible regions of the protein to potentially create new epistatic effects that would allow the system to preferentially bind on-target DNA over off-target DNA. Specifically, we hypothesized that by swapping extruding loops in the Nba-1 REC domain with those derived from other REC domains, we could take advantage of natural variation in REC domains to improve interaction between the inserted REC domain and the guide:target heteroduplex (Fig. 2a). Accordingly, we made swaps in three regions: a potential duplex-facing loop in the conserved REC zinc finger and two RNA–DNA duplex-facing loops, each with conserved flanking residues (Fig. 2d and Supplementary Fig. 1). Individual swaps were created by replacing the sequence between the conserved flanking residues with corresponding sequences from other orthologs identified via multiple sequence alignment (Supplementary Fig. 1).

**Extended Data Fig. 6:**
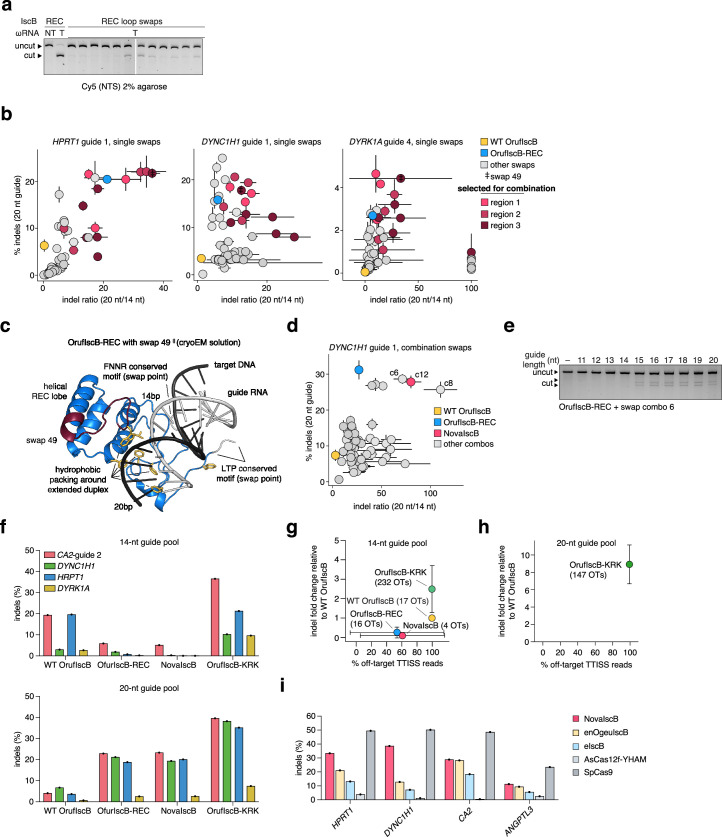
Structure guided engineering of the REC domain in OrufIscB-REC, related to Fig. 2. (**A**) IVTT cleavage assay of OrufIscB-REC with 12 single loop swaps in the REC domain using IVTT protein and WT ωRNA with labeled target (target strand: Cy3; non-target strand: Cy5). Gel was imaged in Cy5 channel only. NTS: non-target strand. (**B**) Indel formation mediated by OrufIscB-REC harboring 52 single loop swaps in the REC domain with either a 20-nt ωRNA or a 14-nt ωRNA targeting HPRT1, DYNC1H1 and DYRK1A. The indel ratio (20 nt/14 nt) and indel activity with the 20-nt guide for each variant are plotted. The selected candidates for further combination tests were labeled in shades of red. Double dagger indicates swap 49. Data are presented as mean +/- SD, n = 3 replicate transfections. (**C**) Cryo-EM ternary complex structure of OrufIscB-REC-swap 49 with a native ωRNA and target DNA heteroduplex with a focus on the hydrophobic interactions between the engineered REC domain and the guide:target heteroduplex. Swap 49 in swap region 3 is shown in red. (**D**) Indel formation mediated by OrufIscB-REC harboring 54 double loop swaps in the REC domain with either a 20-nt ωRNA or a 14-nt ωRNA targeting DYNC1H1. The indel ratio (20 nt/14 nt) and indel activity with the 20-nt guide for each variant are plotted. Variant c12 (red, swap combo 12) was selected as NovaIscB. Data are presented as mean +/- SD, n = 3 replicate transfections. (**E**) In vitro cleavage assay with OrufIscB-REC with swap combo 6 using ωRNAs 11 to 20-nts long. (**F**) On-target indel formation at 4 target sites mediated by WT OrufIscB, OrufIscB-REC, NovaIscB or OrufIscB-KRK in TTISS samples using 14-nt (top) or 20-nt (bottom) guide pool. (**G**) Specificity analysis of OrufIscB-REC, NovaIscB, OrufIscB-KRK and WT OrufIscB with 14-nt guides. % off-target reads is the average of the number of off-target reads divided by total number of reads for each guide from TTISS using a pool of four 14-nt guides. Indel fold change is the average of fold change of percent indels generated relative to WT OrufIscB across the same four guides as measured by amplicon sequencing of the same samples. Error bars represent standard deviation. The numbers of detected off-target sites are included in brackets. (**H**) Specificity analysis of OrufIscB-KRK with 20-nt guides. X and Y axes are calculated as in (G). (**I**) On-target indel formation at 4 target sites mediated by NovaIscB, enOgeuIscB, eIscB, AsCas12f-YHAM and SpCas9 in TTISS samples using individually transfected guides. Source data

To test the feasibility of this engineering strategy, we constructed a pilot set of 12 swaps and evaluated their dsDNA cleavage activity using an IVTT cleavage assay. We observed that approximately half of the tested variants retained some cleavage activity (Extended Data Fig. 6a and Supplementary Table 4). We then comprehensively evaluated the ability of REC domain loop swaps to improve specificity by evaluating their capability to suppress the activity of short guides while maintaining the activity of longer guides in human cells. We constructed a more expansive set of 52 swaps across the three positions (Supplementary Table 4) and tested each variant at 3 target sites in the human genome using both 20-nt and 14-nt guides. Swap 49 produced the largest increase in the ratio between 20-nt guides and 14-nt guides without decreasing activity, indicating that it could be more specific than OrufIscB-REC (Extended Data Fig. 6b).

**Extended Data Fig. 7:**
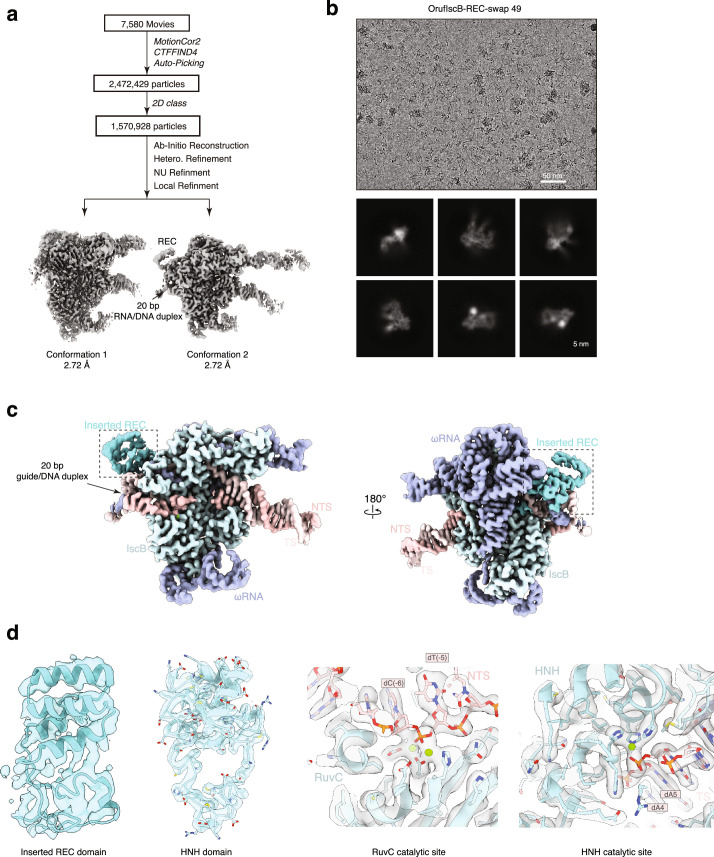
Structural analysis of OrufIscB-REC-swap 49-ωRNA-target DNA complex, related to Fig. 2. (**A**) Flow chart of cryoEM data analysis of OrufIscB-REC-swap 49-ωRNA-target DNA complex including example micrographs of the imaged particles (top row), estimated particle clusters (second row). (**B**) Representative cryo-EM image from 7,580 movies (top) and representative 2D averages (bottom) of the OrufIscB-REC-swap 49-ωRNA-target DNA complex. (**C**) Cryo-EM density of the OrufIscB-REC-swap 49-ωRNA-target DNA complex. Light blue: OrufIscB-REC-swap 49; green, inserted REC domain; purple: ωRNA; pink, DNA non-target strand; light pink, DNA target strand. (**D**) EM density and atomic model of the inserted REC domain, HNH domain, the catalytic site of the RuvC domain and the catalytic site of the HNH domain.

**Extended Data Fig. 8:**
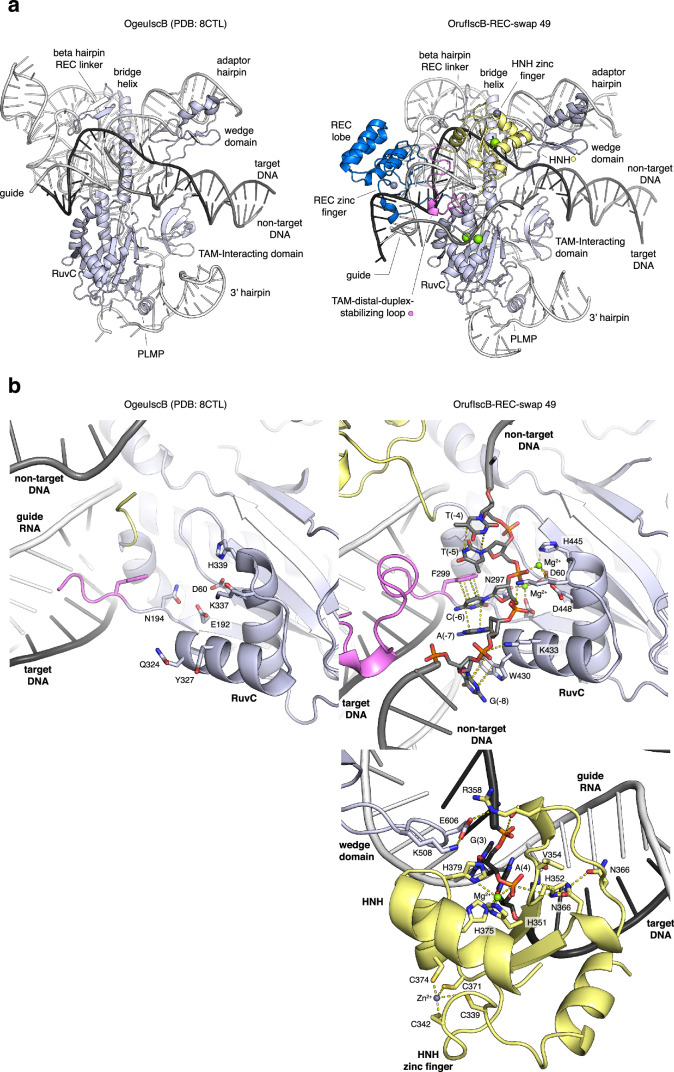
Structural comparison of OrufIscB-REC-swap 49 to OgeuIscB, related to Fig. 2. (**A**) Structural comparison of OgeuIscB (PDB: 8CTL) and OrufIscB-REC-swap 49 RNPs showing conserved protein domain architecture except the engineered REC lobe in OrufIscB-REC-swap 49. (**B**) Close-up view of the interaction between REC domain and extended RNA/DNA duplex and the catalytic cleavage mechanisms on HNH domain in OrufIscB-REC-swap 49.

To understand the mechanism by which the Nba-1 REC plus swap 49 improves specificity in OrufIscB, we solved the cryo-EM structure of OrufIscB-REC–swap 49 (Extended Data Figs. 6c and 7a–d, and Supplementary Table 6). A reconstruction at 2.72-Å resolution revealed that the inserted REC domain extends toward the TAM-distal end of the guide:target heteroduplex (Extended Data Figs. 6c and 7a–e), possibly leading to stabilization of a longer heteroduplex region as indicated by our ability to resolve up to 20 bp of the guide:target heteroduplex compared with only 14 bp visible in structures of OgeuIscB (Extended Data Figs. 7 and 8)^16,17^. Overall folding of the REC domain and the loop swap in the cryo-EM structure were similar to AlphaFold models (Fig. 2d and Extended Data Fig. 6c). Unexpectedly, rather than using polar interactions, the REC insert primarily uses nonpolar interactions to stabilize the extended guide:target duplex with interactions occurring as far as position 20 of the target:guide duplex, potentially explaining why the optimal guide length for OrufIscB-REC–swap 49 is around 20 bp (Extended Data Fig. 6c). We further observed that the REC zinc finger contacts the guide:target duplex, the ωRNA scaffold, the helical REC lobe and the RuvC-II domain, suggesting that it may play a central role in relaying target match and mismatch information to the rest of the protein (Extended Data Fig. 6c). In contrast to OgeuIscB, in which the nontarget strand is relatively flexible, the OrufIscB-REC–swap 49 structure resolves a precise placement of the nontarget strand, which is facilitated by the extended RNA:DNA duplex from the REC domain and further stabilized by the region of F299–G325, which we termed the TAM-distal-duplex-stabilizing loop. F299 from this loop and W430 from the RuvC form pi-stacking interactions with the nontarget DNA strand next to the RuvC catalytic site (DNA positions −6 and −8 respectively) (Extended Data Fig. 8b).

Due to the clear density of both RuvC and HNH endonuclease domains (Extended Data Fig. 7c,d), our structure also reveals the catalytic cleavage mechanisms of IscB, which were not fully elucidated in previous structural studies of IscBs^16–18^. The well-resolved HNH domain in the OrufIscB structure suggests that it is structurally more stable compared with OgeuIscB, in which the HNH domain shows flexibility, which may explain why OrufIscB is more active than OgeuIscB. Unexpectedly, the HNH active site arrangement appears to be distinct from that of Cas9 (Extended Data Fig. 8b). Specifically, the HNH domain of OrufIscB-REC–swap 49 coordinates a single magnesium ion through a tripartite histidine cluster, whereas Cas9 uses an asparagine and an aspartic acid^30^. This magnesium ion is positioned to cleave the phosphate between position 3 and 4 in the target DNA. The HNH domain is further stabilized by the wedge domain through a salt bridge between R358 and E606, suggesting that the wedge domain plays a role in both TAM recognition and pre-cleavage HNH arrangement, potentially contributing to the precise target DNA cleavage pattern shown by IscB^14^. Moreover, as predicted by the AlphaFold2 models and multiple sequence alignments, the HNH domain contains an embedded C4 zinc finger domain that is directly adjacent in sequence to the catalytic H375 residue, a configuration not observed in Cas9 (Extended Data Fig. 8b). The RuvC of OrufIscB-REC–swap 49, on the other hand, appears to use the same active site configuration as the Cas9 RuvC, coordinating two magnesium ions that surround the phosphate group between the −5 and −6 positions on the nontarget strand before cleavage.

Having determined that the REC domain insertion aids with the stabilization of the extended guide:target duplex, we sought to enhance its effect by combining different loop swaps. From the initial test of 52 individual swaps, we identified 3 top candidates from the first region, 4 from the second region and 6 from the third region based both on their overall high activity and high ratios of indel activity with 20-nt guides versus 14-nt guides (Extended Data Fig. 6b). We then constructed all possible combinations of double swaps from the selected single swaps, resulting in 54 double-swap combinations (Supplementary Table 4). We similarly evaluated these combination swaps and identified 3 double swaps that maintained similar on-target activity to OrufIscB-REC while significantly reducing activity with 14-nt guides, resulting in up to ~200-fold difference in activity between 20-nt guides and 14-nt guides (Fig. 2e and Extended Data Fig. 6d). Combinations 6 and 12 emerged as the top two candidates, both of which included swap 49, which is derived from the NbaCas9-2 REC domain in REC region 3, as well as a loop from different type II-D Cas9s from metagenomic sources in REC region 2.

We evaluated the effective guide length of combinations 6 and 12 in vitro and found that they increased the effective guide length to 15 nt and 16 nt, respectively, bringing the effective guide length close to that of SpCas9 in vitro (17 nt) (Fig. 2f and Extended Data Fig. 6e). We selected combination 12, comprising swap 11 in REC region 2 and swap 49 in REC region 3, referred to hereafter as NovaIscB, for continued evaluation and optimization given its longer effective guide length. We assessed the guide length preference of NovaIscB in human cells at 2 target sites, and found that this version further reduced the activity on guides shorter than 17 nt compared with OrufIscB-REC while maintaining similar on-target efficiency using 20-nt guides for both sites (Fig. 2g and Supplementary Table 2).

**Fig. 3:**
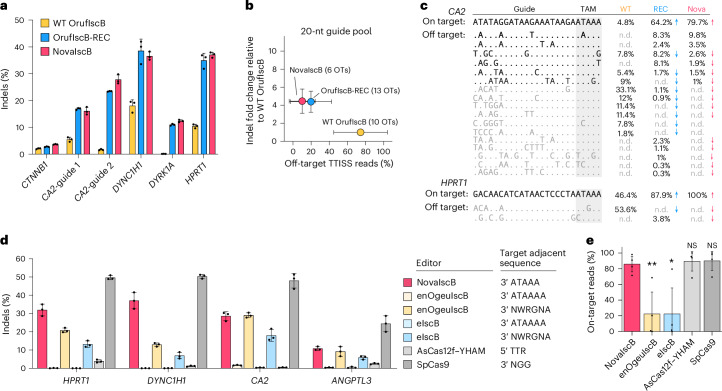
Characterization of NovaIscB activity in mammalian cells. **a**, Indel formation mediated by WT OrufIscB, OrufIscB-REC and NovaIscB using 20-nt guides at six target sites across the human genome. Data are presented as mean ± s.d.; *n* = 3 replicate transfections. **b**, Specificity analysis based on the average indel fold changes of OrufIscB-REC or NovaIscB relative to WT OrufIscB and average percentage of off-target reads per guide from TTISS using a pool of four 20-nt guides each with WT OrufIscB, OrufIscB-REC and NovaIscB. The numbers of detected off-target (OT) sites are included in brackets. Error bars denote s.d.; *n* = 4 target sites. **c**, Sequences of two detected on-target and related detected off-target sites from TTISS. The percentages of reads related to the on-target site and each off-target site out of the total reads corresponding to each guide were included. Mismatched nucleotides in each off-target site relative to each on-target site are shown. Off-targets found with NovaIscB are shown in black. Gray shading denotes the TAM region. n.d., not detected. **d**, Indel formation mediated by NovaIscB, two engineered versions of OgeuIscB (enOgeuIscB and eIscB), AsCas12f–YHAM and SpCas9 with guides targeting *HPRT1*, *DYNC1H1, CA2* and *ANGPTL3*. For NovaIscB, AsCas12f and SpCas9, 20-nt guides were used, and for enOgeuIscB and eIscB, 16-nt guides were used. Data are presented as mean ± s.d.; *n* = 3 replicate transfections. **e**, Fraction of on-target reads measured by TTISS for NovaIscB, enOgeuIscB, eIscB, AsCas12f–YHAM and SpCas9. Each point represents a different guide, individually transfected for each system compared. Data are presented as mean ± s.d. among different target genes; *n* = 3 target sites (AsCas12f–YHAM); *n* = 4 target sites (NovaIscB, enOgeuIscB, eIscB and SpCas9). *P < 0.05; **P < 0.01; NS, *P* > 0.05 from two-sided *t*-test for NovaIscB versus others. The *P* values are 0.00990 (enOgeuIscB), 0.01914 (eIscB), 0.70565 (AsCas12f–YHAM) and 0.66636 (SpCas9).

We then evaluated the on-target activities of WT OrufIscB, OrufIscB-REC and NovaIscB on more target sites using 20-nt guides, and found that both OrufIscB-REC and NovaIscB yielded much improved activities on all the tested sites (from 2-fold to 100-fold compared with WT OrufIscB) in human cells (Fig. 3a). Compared with the previously reported OgeuIscB (Fig. 1d), NovaIscB achieved roughly 100-fold improvement across various target sites.

We compared genome-wide off-target cleavage by WT OrufIscB, OrufIscB-REC, NovaIscB and OrufIscB-KRK with 14-nt and 20-nt versions of 4 guides in a single pooled transfection, which were previously shown to generate efficient indels with the full-length ωRNA scaffold (Fig. 3a) using TTISS^12^. In the pooled TTISS experiment, OrufIscB-REC and NovaIscB showed significantly decreased on-target activity with the 14-nt guides, consistent with previous results, whereas OrufIscB-KRK showed high on-target activity (Extended Data Fig. 6f and Supplementary Table 2). OrufIscB-REC and NovaIscB also achieved significantly improved specificities with 20-nt guides, with NovaIscB performing the best overall (Fig. 3b,c, Extended Data Fig. 6g,h and Supplementary Table 5). We detected more on-target reads and off-target sites for OrufIscB-REC compared with WT OrufIscB, which is most probably owing to the significantly enhanced activity of OrufIscB-REC with 20-nt guides (Fig. 3c). NovaIscB generated more on-target TTISS reads with minimal detected off-target sites for both guide lengths, probably by reducing the off-target sites with TAM-distal mismatches (Fig. 3c).

Finally, we compared NovaIscB with another compact genome editor, AsCas12f–YHAM^34^, two recently developed engineered versions of OgeuIscB (enOgeuIscB^23^ and eIscB^26^) and SpCas9 for both on- and off-target activity. For both engineered OgeuIscB variants, we used guides targeting previously reported efficiently edited sites^23,26^ (NWRGNA TAM) and those matched with NovaIscB-targeted sites (ATAAAA TAM). We observed that while NovaIscB shows approximately 80% of the activity as SpCas9 for comparable target sites, it is 1.0–2.9× more active than enOgeuIscB (at sites with the more optimal NWRGNA TAM), 1.6–5.3× than eIscB (at sites with the more optimal NWRGNA TAM) and 8.1–61.3x than AsCas12f–YHAM at comparable sites (Fig. 3d). Notably, we observed that the activity of both engineered OgeuIscBs relies on the presence of a G as the fourth nucleotide in the TAM sequence.

We additionally compared the specificity of NovaIscB, AsCas12f–YHAM, enOgeuIscB, eIscB and SpCas9 with four guides each assessed in separate transfections. NovaIscB shows comparable genome-wide targeting specificity to both SpCas9 and AsCas12f–YHAM, with 14.3% of TTISS reads attributable to off-target activity with NovaIscB when averaging across 4 guides, compared with 10.2% for SpCas9 and 10.6% for AsCas12f–YHAM, while maintaining similar on-target indel activities for all the editors (Extended Data Fig. 6i). NovaIscB is also significantly more specific than either enOgeuIscB or eIscB (using the more optimal NWRGNA TAM-targeting guides), which both show 77.6% of TTISS reads attributable to off-target activity (Fig. 3e and Supplementary Table 5).

### Structure-guided engineering of the OrufIscB ωRNA

**Fig. 4:**
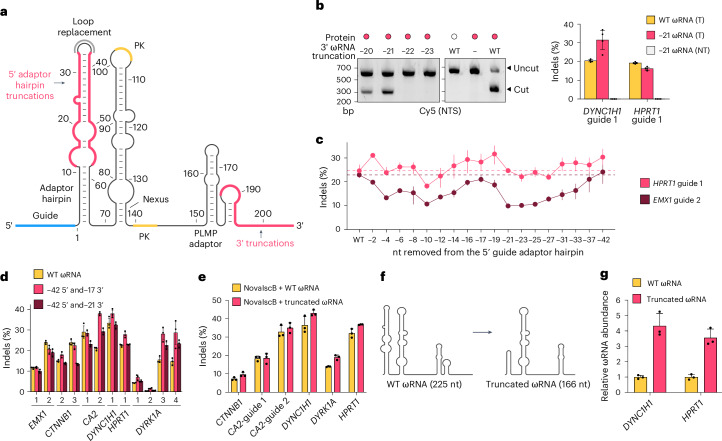
Structure-guided engineering of the ωRNA scaffold. **a**, Schematic of OrufIscB ωRNA secondary structure model. Red, stretches that were considered for deletion; orange, pseudoknot (PK); blue, guide region. **b**, Left: IVTT cleavage assay with WT OrufIscB and 3′ truncated or WT ωRNA. Right: indel formation of WT ωRNA versus 3′ truncated ωRNAs in HEK293FT cells. NTS, nontargeting strand. Data are presented as mean ± s.d.; *n* = 3 replicate transfections. **c**, Indel formation mediated by OrufIscB-KRK in HEK293FT cells using ωRNA scaffolds progressively truncated from the top of the guide adapter hairpin at the 5′ end. Data are presented as mean ± s.d.; *n* = 3 replicate transfections. **d**, Indel formation using ωRNAs with combined 5′ and 3′ truncations versus WT ωRNA mediated by OrufIscB-KRK in HEK293FT cells. Data are presented as mean ± s.d.; *n* = 3 replicate transfections. **e**, Indel formation mediated by NovaIscB with WT ωRNA or combined (42-nt 5′ and 17-nt 3′) truncated ωRNA on different target sites. Data are presented as mean ± s.d.; *n* = 3 replicate transfections. **f**, Schematic of OrufIscB WT ωRNA scaffold and combined truncated ωRNA scaffold. **g**, Normalized ωRNA abundance in HEK293FT cells 4 days after co-transfection with NovaIscB and WT ωRNAs or combined truncated ωRNAs targeting two different genes. Data are presented as mean ± s.d.; *n* = 3 replicate transfections. Source data

**Extended Data Fig. 9:**
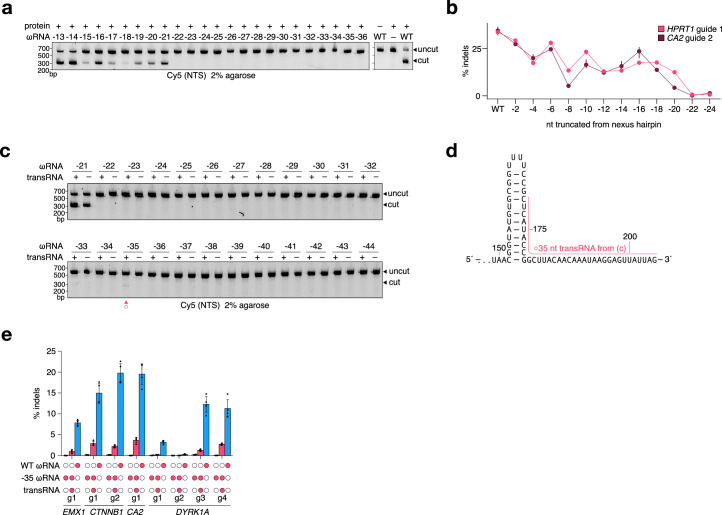
Conditional activation switch of OrufIscB’s activities, related to Fig. 4. (**A**) IVTT cleavage assay with WT OrufIscB and ωRNAs progressively truncated from the 3′ end at single nucleotide resolution. WT: wild-type; NTS: non-target strand. (Note a subset of this data is shown in Fig. 4B). (**B**) Indel formation using ωRNAs with combined 5′/3′ truncations plus truncations in the nexus hairpin using the OrufIscB-KRK protein at two target sites. Data are presented as mean +/- SD, n = 3 replicate transfections. (**C**) IVTT cleavage assay with WT OrufIscB and ωRNAs progressively truncated from the 3′ end at single nucleotide resolution with or without the truncated component included as an additional RNA in trans (transRNA). The red circle indicates cleavage with the 35 nt transRNA. WT: wild-type; NTS: non-target strand. (**D**) Schematic of secondary structure and sequence of OrufIscB ωRNA U149-G205. Red line indicates the sequence truncated and reconstituted in trans as indicated by the red circle in (C). (**E**) Indel formation in HEK293FT cells by OrufIscB-KRK with full-length ωRNA (blue), or ωRNA with 35 nt truncated from the 3′ end, with (red) or without (gray) the truncated 35 nt sequence added back in trans (transRNA). Data are presented as mean +/- SD, n = 4 replicate transfections.

In parallel with our efforts to engineer the inserted REC domain, we also sought to engineer the OrufIscB ωRNA to reduce its size and potentially increase its expression levels through improved stability (step 4 in our engineering process). We reasoned that this would help optimize the system for delivery in limited cargo capacity and limited expression contexts, such as delivery with AAV. We selected the unstructured 3′ end and the 5′ guide adaptor hairpin for further investigation based on the OrufIscB ωRNA cryo-EM structure (Fig. 4a). First, we progressively truncated the 3′ end of the ωRNA at single-nucleotide resolution in an IVTT cleavage assay with WT OrufIscB and observed that the ωRNA could be truncated by up to 21 nt without loss of cleavage activity in vitro and in human cells (Fig. 4b and Extended Data Fig. 9a). Concurrently, we progressively truncated the 5′ guide adaptor hairpin beginning at the top of the hairpin and found that we could remove 42 nt in the hairpin stem (A12–A57 → GAAA) with no apparent loss of activity in human cells when combined with the OrufIscB-KRK protein, which we used in these experiments owing to its high signal in on-target activity assays (Fig. 4c). We also attempted to truncate the nexus hairpin (Fig. 4a) but found that even small truncations reduced activity in human cells (Extended Data Fig. 9b).

From our cryo-EM structure, we observed that G180–C188 appeared to form a short stacked hairpin structure that was not present in the predicted secondary structure, despite our earlier observation that 21 nt (C185–G205) could be removed from the 3′ end. Therefore, we combined the 5′ and 3′ truncations, testing both a 17-nt (A189–G205) and 21-nt (C185–205) truncation on the 3′ end to account for the short 3′ stacked hairpin observed in the cryo-EM structure. We found that removing both 42 nt from the 5′ guide adaptor hairpin and 17 nt from the 3′ end of the ωRNA resulted in maintenance or slight improvement of the indel-generating activity of the OrufIscB ribonucleoprotein (RNP) complex in human cells when combined with OrufIscB-KRK (Fig. 4d). Finally, we combined this shortened 166-nt ωRNA scaffold with the NovaIscB protein and observed robust activity across all guides tested (Fig. 4e). In total, we were able to remove multiple secondary structures, ultimately reducing the size of the ωRNA by 59 nt (Fig. 4f), which yielded improved ωRNA expression levels in human cells by about fourfold compared with the WT ωRNA scaffold (Fig. 4g).

We then sought to exploit the large scaffold of the OrufIscB ωRNA by engineering a conditional activation switch. In our cryo-EM structure, we observed that the longer hairpin (G153–G179) at the 3′ end of the ωRNA contacts the protein only at the base of the hairpin despite our inability to truncate this hairpin without loss of function (Fig. 4b and Extended Data Fig. 9a). We hypothesized that by splitting the hairpin, we would be able to deactivate the function of IscB and reconstitute it through the addition of the missing RNA sequence in *trans*, which we termed a transRNA. In an IVTT cleavage assay, we observed reconstitution of cleavage activity using an ωRNA truncated by 35 nt at the 3′ end (Extended Data Fig. 9c,d). We recapitulated this across 8 tested target sites in the human genome, where the reconstituted ωRNA showed activity up to 20% of that observed with the full-length ωRNA (Extended Data Fig. 9e). TransRNAs provide an additional level of control over the activation of genome editing by OrufIscB.

### OrufIscB is a versatile genome interrogation tool

**Fig. 5:**
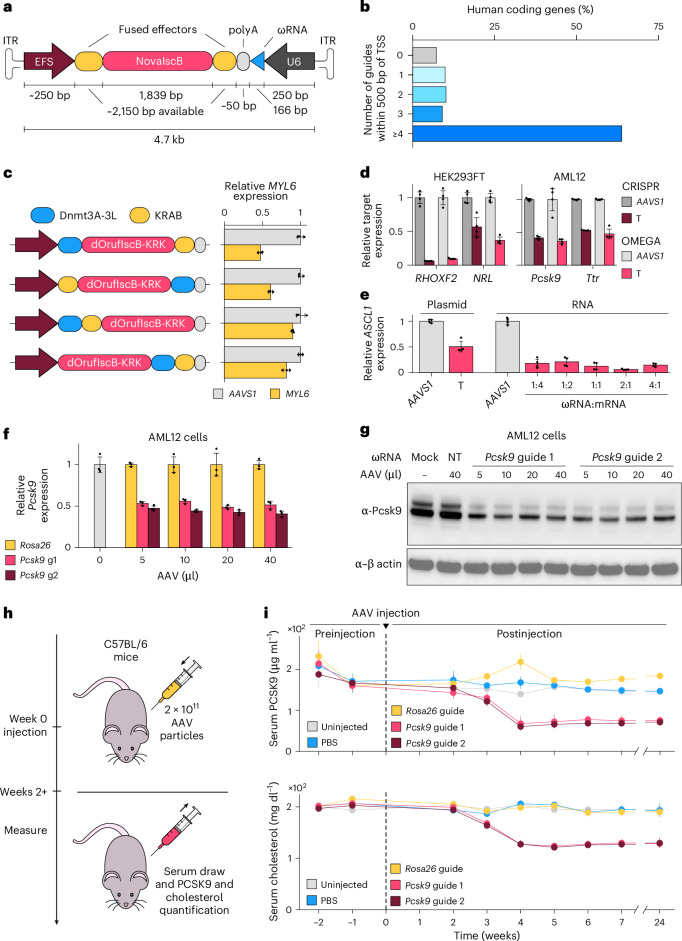
NovaIscB is a platform for compact genome and epigenome modification. **a**, Schematic of AAV packaging capacity when using NovaIscB. EFS, a promoter to drive the expression of protein components. **b**, Distribution of the number of NovaIscB-compatible NTAAA TAM sites within 500 bp of a transcription start site of all human coding genes. TSS, transcription start site. **c**, Left: various protein fusion architectures tested in the OMEGAoff system. Right: Normalized transcript levels of the *MYL6* transcript in HEK293FT cells measured by RT-qPCR for each corresponding fusion architecture with NT (*AAVS1* targeting) or T (*MYL6* targeting) guides. Data are presented as mean ± s.d.; *n* = 3 replicate transfections. **d**, Normalized target transcript levels in HEK293FT or AML12 cells co-transfected with CRISPRoff (SpCas9) and OMEGAoff (OrufIscB-KRK) plasmids with negative control (*AAVS1* targeting) guides or targeting guides as assayed by RT-qPCR. Data are presented as mean ± s.d.; *n* = 4 replicate transfections. **e**, Normalized *ASCL1* transcript levels in HEK293FT cells co-transfected with plasmids or in vitro-transcribed RNAs encoding ωRNAs and OMEGAoff (OrufIscB-KRK) as assayed by RT-qPCR. Data are presented as mean ± s.d.; *n* = 4 replicate transfections. **f**,**g**, Normalized *Pcsk9* transcript levels measured by RT-qPCR (**f**) and PCSK9 protein levels assayed by western blot (**g**) in AML12 cells 1 week after AAV transduction of OMEGAoff (NovaIscB) targeting *Rosa26* (nontargeting) or *Pcsk9* (targeting). Data are presented as mean ± s.d. The error bars denote s.d.; *n* = 3 replicate transductions. **h**, Experimental workflow for in vivo *Pcsk9* repression using AAV delivery of OMEGAoff (NovaIscB). **i**, Time course of serum PCSK9 (top) and total cholesterol (bottom) levels in mice. The error bars denote s.d.; *n* = 4 animals for each condition across all time points. Source data

**Extended Data Fig. 10:**
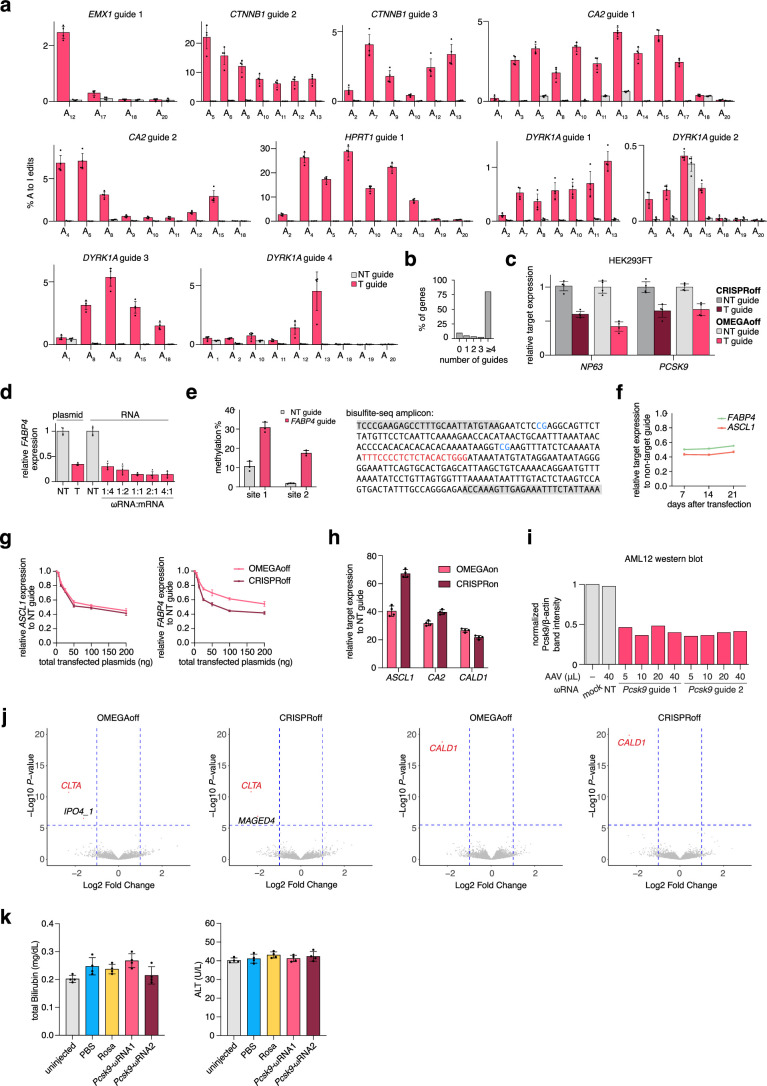
Applications of NovaIscB, related to Fig. 5. (**A**) A-to-G conversion efficiency of OrufIscB-KRK-ABE8e at 10 target sites in HEK293FT cells. Data are presented as mean +/- SD, n = 4 replicate transfections. (**B**) Distribution of the number of guides targeting each human CDS for knockdown by OrufIscB/NovaIscB using an NTAAA TAM. (**C**) Normalized target transcript levels in HEK293FT cells co-transfected with CRISPRoff or OMEGAoff plasmids and negative control (AAVS1 targeting) guides or guides targeting NP63 and PCSK9 as assayed by RT-qPCR. Data are presented as mean +/- SD, n = 4 replicate transfections. (**D**) Normalized target (FABP4) transcript levels in HEK293FT cells transfected with plasmids or in vitro transcribed RNAs encoding ωRNAs and OMEGAoff as assayed by RT-qPCR. Data are presented as mean +/- SD, n = 4 replicate transfections. (**E**) DNA methylation levels at CpG sites in the targeted FABP4 promoter region assayed by bisulfite sequencing (BS-Seq) in HEK293FT cells co-transfected with OMEGAoff plasmids and negative control (AAVS1 targeting) ωRNA or FABP4 targeting ωRNA. Data are presented as mean +/- SD, n = 3 replicate transfections. The amplicon sequence for BS-Seq is included. The guide sequences are labeled as red. The two CpG sites are labeled as blue. Gray shading denotes primer binding regions to make the amplicon. (**F**) Normalized target transcript levels in HEK293FT cells at time points from 1 to 3 weeks after co-transfection of CRISPRoff or OMEGAoff plasmids and guides targeting FABP4 or ASCL1 relative to an AAVS1 targeting negative control guide as assayed by RT-qPCR. Data are presented as mean +/- SD, n = 4 replicate transfections. (**G**) Normalized target (ASCL1) transcript levels in HEK293FT cells transfected with various plasmid amounts of OMEGAoff or CRISPRoff and associated guide RNAs as assayed by RT-qPCR. Data are presented as mean +/- SD, n = 4 replicate transfections. (**H**) Normalized target transcript levels in HEK293FT cells co-transfected with dCas9-VPR or dOrufIscB-KRK-VPR plasmids and negative control (AAVS1 targeting) guides or guides targeting ASCL1, CA2 or CALD1 as assayed by RT-qPCR. Data are presented as mean +/- SD, n = 4 replicate transfections. (**I**) Quantification of PCSK9 protein levels assayed by western blot in AML12 cells after AAV infection of the OMEGAoff targeting Rosa26 (non-targeting) or Pcsk9. Assays were conducted 1 week post infection with different volumes of viruses as indicated, related to Fig. 5G. (**J**) RNA-seq analysis of HEK293FT cells co-transfected with OMEGAoff or CRISPRoff plasmids and guides targeting CLTA or CALD1 relative to untransfected. The comparison of target guides vs negative control was conducted by DESeq2 and displayed as volcano plots. The differential expressed transcripts (-Log10P-value > 5.5, Log2 fold change < -1 or > 1) were labeled, n = 2 replicate transfections. (**K**) Liver function tests (total Bilirubin and ALT assays) of serum samples from uninjected, PBS injected, Rosa26-targeting, and Pcsk9-targeting ωRNAs injected mice at the time point of 24 weeks (6 months) after injection. Data are presented as mean +/- SD, n = 4 animals.

The compact size of NovaIscB (614 aa) and associated ωRNA variant (166 nt) allows for the inclusion of both fused domains and an ωRNA expression cassette into a single AAV genome (Fig. 5a). To develop such a platform, in parallel with our development of NovaIscB, we tested the compatibility of OrufIscB with both base editing and targeted methylation. We first tested adenine base editing by fusing a RuvC-inactivated (D61A) OrufIscB-KRK to ABE8e^35^ and assessing its ability to perform A-to-G edits at 12 sites using full-length ωRNA scaffolds at various sites in the human genome containing adenines at a variety of positions. We found that OrufIscB-KRK–ABE was capable of base editing at all targets tested with varying efficiencies (Extended Data Fig. 10a and Supplementary Table 7). The editing window typically included positions 3–17, counted from the 5′ end of the guide sequence, which is slightly larger than previous observations with both IscB and Cas9 base editors^35^.

Next, we developed an epigenome editing and transcriptional repression system using OrufIscB-KRK. Despite the seemingly restrictive NTAAA TAM of OrufIscB, 90% of human genes can theoretically be targeted for gene knockout (Extended Data Fig. 10b) while 93% of human genes can theoretically be targeted for methylation-based knockdown using the flexible targeting window of ±500 bp from the transcription start site for transcriptional repression via epigenome editing (Fig. 5b)^36^. We fused dOrufIscB-KRK (RuvC and HNH inactivated OrufIscB-KRK, D61A/H347A) with the DNA methyltransferase 3A (Dnmt3A) and DNA methyltransferase 3-like (Dnmt3L) DNA methylation writers and a Krüppel associated box (KRAB) transcriptional repression domain, which together have been adopted for the CRISPRoff system with SpCas9 (ref. ^36^). We tested different fusion architectures of these proteins with dOrufIscB-KRK by transient plasmid transfection and found that an N-terminal Dnmt3A-3L and C-terminal KRAB, the same architecture as CRISPRoff-V2 (ref. ^36^), achieved the best repression activity when targeting the promoter of *MYL6* (Fig. 5c). We termed this construct OMEGAoff; we assessed the ability of OMEGAoff to repress the expression of various genes in the human and mouse genomes in HEK293FT and alpha mouse liver 12 (AML12) cells, respectively, including the clinically relevant genes *Pcsk9* and *Ttr*^37,38^, and compared OMEGAoff with CRISPRoff-V2.1 (ref. ^36^). We found that OMEGAoff was capable of repressing transcription of all targeted genes to similar levels as CRISPRoff-V2.1 as measured by quantitative reverse transcription PCR (RT-qPCR) (Fig. 5d and Extended Data Fig. 10c). We also achieved strong repression when delivering the OMEGAoff components as RNA targeting *ASCL1* and *FABP4*, with optimal repression observed using a 2:1 mass ratio of ωRNA to mRNA (Fig. 5e and Extended Data Fig. 10d). We then performed bisulfite sequencing on the targeted *FABP4* promoter region and observed elevated DNA methylation levels compared with a control (*AAVS1* targeting) ωRNA at CpG sites in the *FABP4* promoter region (Extended Data Fig. 10e), suggesting that the observed repression is due to OMEGAoff-mediated methylation in this region. We performed a time course to evaluate the stability of repression by OMEGAoff and observed repression up to 21 days after transient plasmid transfection, suggesting that the altered methylation patterns result in durable repression even after loss of OMEGAoff expression (Extended Data Fig. 10f). In addition, we titrated the total amount of plasmids transfected for both OMEGAoff and CRISPRoff and observed a similar trend for the target repression levels of *ASCL1* and *FABP4* with both systems, with a plateau in target repression around 100 ng transfected per well in a 96-well plate (Extended Data Fig. 10g).

To explore the capability of gene upregulation, we fused dOrufIscB-KRK with VP64, p65 and Rta (VPR), a configuration adopted by dCas9-based OMEGAon^36^. We found that this system, termed OMEGAon, was able to increase the expression of target genes significantly and to similar levels as CRISPRon (Extended Data Fig. 10h). Together, these results show the flexibility of OrufIscB-KRK to mediate diverse genome interrogation modalities. Furthermore, the mechanistic similarities between IscB and Cas9 apparently enable straightforward construct design using systems previously optimized for Cas9.

### In vivo epigenome editing with NovaIscB

Finally, we applied OMEGAoff in vivo by targeting *PCSK9* in the liver, which is relevant for cholesterol regulation^39^. We first tested the OMEGAoff system with our most specific, efficient and compact IscB composition, NovaIscB, in cultured cells. We fused dNovaIscB (RuvC and HNH inactivated NovaIscB) with Dnmt3A-3L and KRAB to create an optimized OMEGAoff design and packaged this system in a single vector using the hepatocyte-tropic AAV serotype 8. Using the truncated 166-nt ωRNA scaffold, we tested two guides targeting sites adjacent to CTAAA and TTAAA sequences in the promoter of mouse *Pcsk9*, as there were no sites with ATAAA sequences present in this region. A *Rosa26*-targeting guide served as a negative control. We transduced AML12 cells, a mouse hepatocyte line, with various volumes of AAV and found that both ωRNAs enabled twofold repression of *Pcsk9* expression as measured by RT-qPCR (Fig. 5f and Supplementary Table 7). In addition, both the pro-PCSK9 and mature PCSK9 protein levels were significantly decreased as measured by western blot, indicating that repression of target genes by AAV-delivered OMEGAoff can yield functional outcomes (Fig. 5g and Extended Data Fig. 10i). Increasing the volume of AAV did not increase repression, suggesting that robust target repression can be achieved even with low doses. Although we find that NTAAA TAMs are compatible with OMEGAoff, the TAM requirements for the NovaIscB nuclease and other fusions may vary in human cells.

We additionally assessed the global specificity of OMEGAoff and CRISPRoff by performing RNA sequencing (RNA-seq) of cells co-transfected with ωRNAs or sgRNAs (single guide RNAs), respectively, targeting the promoters of *CLTA* or *CALD1*. Both OMEGAoff and CRISPRoff repressed target genes in a highly specific manner relative to nontargeting guides (Extended Data Fig. 10j), consistent with previous results for the CRISPRoff system^36^.

On the basis of these results, we intravenously injected mice with AAVs encoding OMEGAoff with either the *Pcsk9*- or *Rosa26*-targeting guides at a dosage of 2 × 10^11^ total viral particles per animal and collected blood samples to isolate serum for measuring PCSK9 protein and cholesterol levels (Fig. 5h). Beginning at 3 weeks postinjection, we observed a significant decrease in serum PCSK9 levels in mice receiving the *Pcsk9*-targeting guide compared with uninjected, PBS-treated and *Rosa26*-targeting controls (Fig. 5i). This decrease persisted over the 6-month observation period (Fig. 5i and Supplementary Table 7). Similarly, we observed a significant decrease in serum cholesterol levels beginning 4 weeks after injection that persisted for the duration of the study, achieving a similar range of reduction compared with clinical outcomes of PCSK9 monoclonal antibodies^40^ (Fig. 5i). To evaluate the toxicity of OMEGAoff, we measured the serum total bilirubin and alanine aminotransferase (ALT) levels at the 6-month time point. We did not observe significant changes in the *Pcsk9*-targeting mice compared with the uninjected, PBS-treated and *Rosa26*-targeting controls (Extended Data Fig. 10k). This study highlights the potential of using OMEGAoff for persistent gene repression and epigenome editing.

## Discussion

In this study, we combined engineering strategies to generate an optimized, compact (614 aa) IscB, NovaIscB, and applied it to create a compact epigenome editor. NovaIscB was built on OrufIscB, the native ortholog that showed the highest activity in our natural diversity screen. We note that more orthologs might have activity in human cells with shorter guides, but given our focus on generating a high-specificity variant, we performed our ortholog screen with 20-nt guides. This was borne out by our later observations that for IscBs, high activity appears to come hand in hand with low specificity.

To address this limitation, we used an evolution- and structure-guided engineering strategy to insert and subsequently engineer a short exogenous domain, the REC domain, from related proteins, including other IscBs and Cas9s. Though source proteins for these REC domains often lacked efficient genome editing activity in human cells, many REC domains maintained or improved activity when combined with OrufIscB, highlighting the use of natural diversity beyond ortholog screening. We also observed that larger REC domains such as those from type II-A, II-B and II-C Cas9s, such as SpCas9 and FnCas9, maintained cleavage activity when grafted into OrufIscB, but substantially reduced the mammalian genome editing activity, potentially owing to disruption of the FNNR motif and beta hairpin REC loop folding (Extended Data Fig. 3). We continued to engineer the top REC domain variant by swapping regions from REC domains of other proteins to further improve characteristics of OrufIscB relevant for genome editing. In addition, we refined the ωRNA through structure-guided truncations, resulting in higher expression levels in human cells and rendering the ωRNA short enough to enable chemical synthesis and modifications of the ωRNA, making this system compatible with RNA-based delivery platforms. By engineering the ωRNA scaffold and recombining protein parts from systems with low genome editing activity, we achieved a compact, high-activity, effective guide length-extended version of OrufIscB, NovaIscB, that optimally uses 20-nt guides while detecting mismatches along this extended duplex, enabling the simultaneous improvement of both activity and specificity.

Recent efforts from other groups to develop IscB-based genome editors have focused on engineering OgeuIscB^21,26^, which we previously observed to possess mammalian genome editing activity^14^, or conducting small-scale ortholog screens to identify additional human cell-active IscB proteins^23^. Two main strategies have been used in these studies to enhance the activity of IscB–ωRNA complexes: mutagenesis of the IscB protein and truncation of the ωRNA scaffold. While these strategies have improved IscB activity in human cells to clinically relevant levels, mutagenesis of the protein scaffold in particular apparently results in increased nonspecific DNA affinity of the complex, reducing genome-wide targeting specificity compared with WT^23,24,26^. By contrast, by first screening hundreds of orthologs for those with substantial activity using longer guides and, second, adding a REC domain, which is known to be involved in guide:target heteroduplex mismatch detection in the related Cas9 family^30,33^, we engineered an IscB variant, NovaIscB, with both high specificity and activity. We anticipate that further engineering of the inserted REC domain and design of the IscB-REC domain graft to improve allosteric regulation of REC domain function may allow for further optimization along both parameters. Comparison of NovaIscB with these and other compact RNA-guided DNA-targeting systems (enOgeuIscB, eIscB and AsCas12f–YHAM), which present comparable benefits as each other relative to the established SpCas9 system, showed that NovaIscB possesses a combination of both high activity and high specificity not shown by any other compact system we tested. Further assessment of off-target profiles using an alternative method to tag integration, which may be subject to biases owing to differing target sequences and cleavage patterns across guides and systems, will aid in more comprehensive benchmarking of the numerous available genome editors. Although SpCas9 remains a gold standard for highly efficient genome editing, the compact size of NovaIscB compared with that of SpCas9 renders this system ideal for further development for in vivo delivery.

We furthermore showed that NovaIscB can be fused with a methyltransferase and a transcriptional repressor to create a compact OMEGAoff construct. OMEGAoff can be delivered with a single AAV to facilitate stable epigenetic modifications resulting in targeted transcriptional repression in vivo. Moreover, the repression of target genes is maintained over the course of multiple months, possibly owing to the stable expression of AAV and the lasting effect of epigenome modifications. Recent work has shown long-term repression of *Pcsk9* by epigenome editing using zinc finger proteins fused to the same methyltransferase and transcriptional repression domains used here, with lipid nanoparticles as the delivery platform in vivo^41^. The ability to deliver OMEGAoff with AAV opens the door to efficient epigenome modulation in specific tissues that can be targeted with diverse AAV serotypes, and in contrast to zinc finger proteins, the ease of programmability of OMEGAoff provides more flexibility for rapid development of compositions targeting different genes of interest. Moreover, OMEGAoff achieves long-lasting knockdown without introducing DNA breaks or nicks, a potentially safer alternative to traditional gene editing tools that cut DNA. Furthermore, as OrufIscB originates from a human gut metagenome sample, it may show reduced immunogenicity; however, assessment of the immunogenicity of NovaIscB and other IscB-based genome editors will be a critical future step toward their in vivo use.

Finally, we reasoned that, although the NTAAA TAM of NovaIscB permits the targeting of 90% of human genes for gene knockout (Extended Data Fig. 10b) and 93% of human gene promoters for transcriptional regulation (Fig. 5b), expanding the TAM or improving targeting efficiency with noncanonical TAMs would render NovaIscB as a more versatile tool. We additionally have shown the potential of NovaIscB to target a shortened TAM in vitro via mutagenesis of TAM-interacting residues in NovaIscB (Supplementary Fig. 2). However, the TAM of NovaIscB is still relatively restrictive, and looking forward, further engineering of the NovaIscB TAM requirement using domain swapping strategies or protein design to be more permissive could expand the use of this tool across more diverse genomic targets. Engineering the fusions of deaminases, reverse transcriptases or any other domains harnessing endogenous machinery^42^ with NovaIscB could also further optimize the use of NovaIscB for base editing, prime editing and epigenome editing applications in which delivery with a single AAV is advantageous.

Combining the ortholog screen, REC domain selection, mutagenesis, REC loop swaps and ωRNA scaffolds, we investigated more than 1,000 variants in total to create NovaIscB, a promising scientific and therapeutic candidate in the genome editing toolbox. The combined evolution- and structure-guided protein engineering approach used to create NovaIscB provides a framework for dramatically optimizing protein functions, with potential applications extending far beyond genome editing.

## Methods

### Selection and curation of IscB orthologs

The extended database of IscB sequences generated previously^14^ was collected, resulting in diverse genomic and metagenomic loci containing IscB proteins along with their closest 50% sequence identity cluster representative in the previously described IscB, IsrB and Cas9 phylogenetic tree. For each locus that was selected to be experimentally tested, the full IscB system was generated as follows. First, the putative IscB coding sequences and ωRNAs, as previously determined, were refined as follows. All protein sequences within the same 50% sequence identity cluster were aligned to the candidate IscB protein using multiple alignment using fast Fourier transform (MAFFT)^43^. If the candidate IscB protein contained a large (≥50 aa) C-terminal insertion relative to the other proteins within the cluster, the locus was discarded. If the candidate IscB protein contained an N-terminal insertion (≥10 aa) but contained a downstream start codon site that would eliminate the insertion without removing any of the conserved N-terminal PLMP domain (previously named after the frequently observed PLMP amino acid motif in this domain), the downstream start site was selected in place of the computationally determined start site. For metagenomic sequences with multiple related protein sequences within the same 95% sequence identity cluster, all proteins within the cluster were aligned using MAFFT^43^. The most accurate IscB protein sequence was determined to be the one that most closely matched the consensus sequence of this alignment. If the candidate IscB locus did not contain the most accurate IscB protein sequence, the candidate locus was switched to the locus that contains the most accurate IscB protein sequence. For determination of ωRNA boundaries, the upstream region of the IscB protein coding sequence was aligned using MAFFT^43^ to loci from the same 50% sequence identity IscB protein cluster as well as phylogenetically related loci as determined by the previously determined phylogenetic tree^14^. The 3′ boundary of the ωRNA was selected to be ~2 bp upstream from the protein start site to match experimentally observed ωRNA boundaries. The 5′ end of the ωRNA was selected as the 5′ end determined by the CMAlign covariance model for the corresponding ωRNA type^14^ if the first two bases of the 5′ of the CMAlign model matched the first two bases of the ωRNA coding sequence in the given locus. However, in cases in which the model and the candidate ωRNA did not agree at the 5′ end, the ωRNA 5′ location was determined to be the 5′ most position where a sharp increase in conservation in the alignment was observed, signifying the beginning of the ωRNA. In cases in which the ωRNA 5′ could not be resolved, the candidate locus was discarded.

Multiple criteria were used to select the initial set from the large set of possible IscBs to be tested experimentally. The main criterion was phylogenetic diversity—we sampled systems from representative branches across the previously described phylogenetic tree^14^. The next criterion was human-related pathogens. For this subset, NCBI taxon IDs were matched when available to candidate IscB loci when available. IscB loci belonging to bacteria known to have human hosts were selected for this round. For another criterion, IscBs with REC-like insertions were prioritized. For this subset, IscBs were aligned using MAFFT^43^ and the alignment columns between the bridge helix and RuvC-II domains were inferred as REC-like insertions. Candidate IscBs with insertions (>20 aa) in this region were selected for their potential REC-like domains. For the second set, we selected IscB systems based on similarity along the tree to other systems that we found had genome editing activity in human cells.

AlphaFold2 models of tested orthologs were generated as follows. Each tested protein sequence was searched against the full dataset of all IscBs and Cas9s described previously^14^ using MMSeqs2 keeping the top 501 protein hits (sorted by *e*-value) beyond the query protein^44^. Alignments were generated using clustal omega^45^ and used as input multiple sequence alignments for AlphaFold2 running under ColabFold package^27,46^ without the use of templates and with up to 16 recycles using model 3, stopping if the pLDDT exceeds 95 and using Amber relaxation for the side chains.

A phylogenetic tree of the main tested type II-D and IscB orthologs, excluding CasIscBs, though including TbaIscB due to its relationship as the founding member of type II-D Cas9s, was constructed as follows. All tested IscBs were aligned with MAFFT-einsi^43^, and then alignment columns with >50% gaps were removed. The processed alignment was then used to create a phylogenetic tree using IQ-Tree2 default parameters with 2,000 ultra fast bootstraps and using the optimal substitution model determined by ModelFinder^47,48^. Protein length and ωRNA length for each system were determined based on the manually curated sequences for the protein and ωRNA, respectively. IVTT-determined TAM sequences and human genome editing activity (through the multi-guide panel) were determined experimentally as described in the sections below.

### Cell-free transcription–translation TAM interference assay

IscB protein sequences were human codon optimized using the GenScript codon optimization tool. IscB genes and ωRNA scaffolds were custom synthesized by Twist Biosciences, and transcription–translation templates were generated by PCR from custom synthesis products. Cell-free transcription–translation reactions were carried out using a PURExpress In Vitro Protein Synthesis Kit (NEB) as per the manufacturer’s protocol with half-volume reactions, using 75 ng of template for the protein of interest, 125 ng of template for the corresponding ωRNA with a guide targeting the TAM library and 25 ng of TAM library plasmid. Reactions were incubated at 37 °C for 4 h, then quenched by placing at 4 °C or on ice and adding 10 µg RNase A (Qiagen) and 8 units Proteinase K (NEB) each followed by a 5-min incubation at 37 °C. DNA was extracted by PCR purification columns and adaptors were ligated using an NEBNext Ultra II DNA Library Prep Kit for Illumina (NEB) using the NEBNext Adaptor for Illumina (NEB) as per the manufacturer’s protocol. Following adaptor ligation, cleaved products were amplified specifically using one primer specific to the TAM library backbone and one primer specific to the NEBNext adaptor with a 12-cycle PCR using NEBNext High Fidelity 2X PCR Master Mix (NEB) with an annealing temperature of 63 °C, followed by a second 18-cycle round of PCR to further add the Illumina i5 adaptor. Amplified libraries were gel extracted, quantified by qPCR using a KAPA Library Quantification Kit for Illumina (Roche) on a StepOne Plus machine (Applied Biosystems, Thermo Fisher Scientific) and subject to single-end sequencing on an Illumina MiSeq with read 1 80 cycles, index 1 8 cycles and index 2 8 cycles. TAMs were extracted and the enrichment score for each TAM or PAM was calculated by filtering for all TAMs or PAMs present more than once and normalizing to the TAM or PAM frequency in the input library subject to the same IVTT and quenching reactions. A position weight matrix based on the enrichment score was generated, and both WebLogos and Krona plots were visualized based on this position weight matrix using a custom Python script.

### Mammalian cell culture and transfection

Mammalian cell culture experiments were performed in the HEK293FT line (Thermo Fisher Scientific) and AML12 line (CRL-2254, ATCC). HEK293FT cells were grown in Dulbecco’s modified Eagle medium with high glucose, sodium pyruvate and GlutaMAX (Thermo Fisher Scientific). AML12 cells were grown in Dulbecco’s modified Eagle medium/Nutrient Mixture F-12 (Thermo Fisher Scientific), supplemented with 40 ng ml^−1^ dexamethasone (Sigma-Aldrich) and 1× Insulin–Transferrin–Selenium (Thermo Fisher Scientific). All cells were additionally supplemented with 1× penicillin–streptomycin (Thermo Fisher Scientific), 10 mM HEPES (Thermo Fisher Scientific) and 10% fetal bovine serum (VWR Seradigm). All cells were maintained at confluency below 80%.

For DNA transfection, all transfections were performed with Lipofectamine 3000 (Thermo Fisher Scientific). Cells were plated 16–20 h before transfection to ensure 90% confluency at the time of transfection. For 96-well plates, cells were plated at 20,000 cells per well, and for 24-well plates, cells were plated at 100,000 cells per well. For each well on the plate, transfection plasmids were combined with 2 µl of P3000 solution per every 1 µg DNA and OptiMEM I Reduced Serum Medium (Thermo Fisher Scientific) to a total of 25 µl. Separately, 23 µl of OptiMEM was combined with 2 µl of Lipofectamine 3000. Plasmid and Lipofectamine solutions were then combined and pipetted onto cells.

For RNA transfection, all transfections were performed with Lipofectamine MessengerMAX transfection reagent (Thermo Fisher Scientific). Cells were plated similarly to the DNA transfections described above. For each well of 96-well plates, a total amount of 200 ng RNA was combined with 0.6 µl Lipofectamine MessengerMAX reagent and OptiMEM to make 10 µl of transfection mixture, which was pipetted onto cells. For different mass ratios (1:4, 1:2, 1:1, 2:1, 4:1) of in vitro-transcribed ωRNAs to mRNAs (OMEGAoff construct), ωRNA and mRNA were combined at the indicated ratio in a total of 200 ng RNA. ωRNA templates were amplified using Q5 High-Fidelity DNA Polymerase (NEB) and purified with QIAquick spin columns (Qiagen), and RNA was transcribed using a HiScribe T7 Quick High Yield RNA Synthesis Kit (NEB) and purified using an RNA Clean & Concentrator-25 Kit (Zymo Research). For mRNA encoding the OMEGAoff protein, we first digested a plasmid encoding the protein using AanI (Thermo Fisher Scientific) to obtain a linear DNA fragment. In vitro transcription (IVT) reactions were assembled with T7 buffer (NEB), 100 mM ATP (NEB), 100 mM GTP (NEB), 100 mM CTP (NEB), 100 mM pseudo-UTP (Trilink), CleanCap AG (Trilink) and T7 RNA Polymerase (NEB) and incubated at 37 °C for 5 h. The reaction was further treated with TURBO DNAse enzyme (Thermo Fisher Scientific) followed by LiCl (Thermo Fisher Scientific)-based purification before transfection as described.

### Mammalian genome editing

ωRNA scaffold backbones were cloned into a pUC19-based human U6 expression backbone by Gibson Assembly. Human codon-optimized IscB genes were cloned into an immediate early promoter enhancer of cytomegalovirus (CMV) expression backbone by Gibson assembly using 2X Gibson Assembly Master Mix (NEB) to generate pCMV-SV40 NLS-IscB protein-nucleoplasmin NLS-3xHA constructs. For initial testing, 12-guide libraries were cloned in a pool mixing primers to add each of the 12 guides in a given pool at equimolar ratios, and ωRNA scaffold backbones were subject to whole plasmid amplification with guide primers annealing to the U6 promoter and a second primer annealing to the start of the ωRNA scaffold using Phusion Flash High-Fidelity 2X Master Mix (Thermo Fisher Scientific). PCR products were gel extracted and eluted in 30 μl, then blunt-end ligated to circularize by addition of 5 units T4 PNK (NEB), 200 units T4 DNA Ligase (NEB) and final 1X T4 DNA Ligase Buffer (NEB) followed by incubation for 1.5 h at room temperature before transformation in Stbl3 chemically competent *Escherichia coli* (NEB). For individual guide constructs, oligos with appropriate overhangs were synthesized by Genewiz, annealed and phosphorylated using T4 PNK (NEB) and cloned into ωRNA backbones by restriction–ligation cloning. Human codon-optimized IscB genes were cloned into a CMV expression backbone by Gibson assembly using 2X Gibson Assembly Master Mix (NEB) to generate pCMV-SV40 NLS-IscB protein-nucleoplasmin NLS-3xHA constructs.

Before individual guides were tested, each tested IscB protein was screened for activity in HEK293FT cells using a pool of 12 guides cloned as described. For this 12-guide pooled initial screening of IscB proteins, 800 ng of protein expression construct and 800–1,200 ng of the corresponding guide pool with corresponding ωRNA scaffold were transfected in one well of a 24-well plate as described. After 60–72 h, genomic DNA was collected by washing the cells once in 1× Dulbecco’s phosphate buffered saline (DPBS) (Sigma-Aldrich) and dry trypsinizing cells using TrypLE (Thermo Fisher Scientific). Trypsinized cells were collected in 1 ml 1× DPBS and pelleted by centrifugation at 300 × *g* at 4 °C for 5 min. The supernatant was removed, and cells were resuspended in 50 μl QuickExtract DNA Extraction Solution (Lucigen) and cycled at 65 °C for 15 min, 68 °C for 15 min and then 95 °C for 10 min to lyse cells. Then, 2.5 µl of lysed cells was used as input into each PCR. Amplification of each region targeted by a guide in a given guide pool was performed individually.

For all experiments in which individual guide sequences were used, unless otherwise indicated below, 100 ng guide expression plasmid and 100 ng protein expression plasmid were transfected in each of 3 or 4 wells as indicated as biological replicates in a 96-well plate for each guide condition as described. For the experiments in Fig. 2g, 50 ng guide expression plasmid and 100 ng protein expression plasmid were transfected. After 60–72 h, genomic DNA was collected directly without any enrichment of editing events by washing the cells once in 1× DPBS (Sigma-Aldrich) and adding 50 μl QuickExtract DNA Extraction Solution (Lucigen). Cells were scraped from the plates to suspend in QuickExtract and cycled at 65 °C for 15 min, 68 °C for 15 min and then 95 °C for 10 min to lyse cells. Subsequently, 2.5 µl of lysed cells was used as input into each PCR.

For library amplification, target genomic regions were amplified with a 12-cycle PCR using NEBNext High Fidelity 2X PCR Master Mix (NEB) with an annealing temperature of 63 °C for 15 s, followed by a second 18-cycle round of PCR to add Illumina adapters and barcodes. The libraries were gel extracted and subject to single-end sequencing on an Illumina MiSeq with read 1 300 cycles, index 1 8 cycles and index 2 8 cycles. Indel frequency was analyzed using CRISPResso2 (ref. ^49^), with a quantification window center of −9 and a window size of 6 based on a previous analysis of IscB cleavage patterns^14^. To eliminate noise from PCR and sequencing error, only indels with at least two reads or more than one base inserted or deleted were counted toward reported indel frequencies. For 12-guide pooled screens, read alignments were further inspected manually for presence of ‘true’ indels to select candidates for validation. For individual guide–ωRNA experiments, to assess statistical significance, two-tailed *t*-tests were performed using nontargeting guide–ωRNA conditions as a negative control. All indel data are available in Supplementary Table 2. Base editing frequency was analyzed using a previously reported Python script^50^, and all data are available in Supplementary Table 7. All the primer sequences used for genome PCR were listed in Supplementary Table 8.

### Cell-free transcription–translation cleavage assays

To test the cleavage activities of OrufIscB with various REC insertions and NovaIscB variants on target dsDNA with different TAM sequences, cell-free transcription–translation reactions were performed using a PURExpress In Vitro Protein Synthesis Kit (NEB) with half-volume reactions, using 75 ng of template for the NovaIscB variant of interest, 125 ng of template for the ωRNA and 50 ng of target dsDNA. IVTT templates for IscB variants and ωRNAs were prepared as described in Cell-Free Transcription–Translation TAM Interference Assay. Target DNA was prepared by amplifying a pUC19 plasmid containing the target sequence and adjacent TAM with Cy3 and Cy5 primers (IDT) using Q5 Hot Start Hi-Fidelity 2X Master Mix (NEB) as per the manufacturer’s protocol with 3% DMSO added. The reactions were incubated at 37 °C for 4 h, then quenched using 10 µg RNase A (Qiagen) and 1 μl Proteinase K (Qiagen) by a 5 min incubation at 37 °C. DNA was extracted by PCR purification (Qiagen), run on 4% E-gels (Thermo Fisher Scientific) and imaged on a BioRad Chemidoc in the Cy5 channel to visualize cleavage products.

### Cell-free transcription–translation mismatch tolerance assay

The target library was designed by selecting 100 random sites from coding sequences in the human genome (hg38 assembly) adjacent to ATAAA TAMs. Target sites were selected to avoid homopolymeric sequences of four or more Ts or Gs to avoid guides with potential termination signals for later use with PolIII promoters and those with potential to form G quadruplexes. Targets were also selected to have an edit distance of at least 3 away from all other targets in the TAM-proximal 7 bp and an edit distance of at least 10 in total. All possible single mismatches for each target were then generated, and progressive mismatch targets were also generated by selecting 8 sequences each with TAM-distal mismatches ranging from 2 to 7 bp at the 5′ end of the target, with an edit distance of at least 8 from any of the nontemplate original targets in the library. A total of 316 random sequences with an edit distance of at least 10 from all other library members were added as negative controls. In addition, 10 N randomized barcodes were added to each individual target for distinguishing target identity after cleavage. The library was synthesized by GenScript and cloned into a pUC19 vector by Gibson Assembly.

An associated guide library was synthesized by IDT and cloned into a backbone containing the OrufIscB ωRNA scaffold by Gibson Assembly. In vitro transcription templates for the pooled guide library were then generated by PCR and were transcribed using a HiScribe T7 Quick High Yield RNA synthesis kit (NEB). IVTT templates for IscB proteins with REC domain insertions were prepared as described in Cell-Free Transcription–Translation TAM Interference Assay. Cell-free transcription–translation reactions were carried out using a PURExpress In Vitro Protein Synthesis Kit (NEB) as per the manufacturer’s protocol with half-volume reactions, using 75 ng of template for the protein of interest, 1.5 μM final concentration of the in vitro-transcribed ωRNA guide library and 25 ng of target library plasmid. Cas9 and a guide targeting a nonlibrary control were also included as an internal activity control for downstream library preparation and sequencing. Reactions were carried out and libraries were prepared as in Cell-Free Transcription–Translation TAM Interference Assay. Barcodes were extracted and used for quantifying reads using a custom Python script. The relative activity score was calculated by dividing the cleaved read count for each perfectly matched target by the read count of the Cas9 target cleaved in each reaction, then each of those quotients was divided by the normalized read count of the same cleaved target in the WT condition in the same sequencing run. The median for each REC insertion was plotted, and any variants with an activity score of 0.5 or greater was assessed for effective guide length as measured by increasing mismatch tolerance at the TAM-distal end of the guide. For mismatch targets, the read count of all mismatch targets was normalized to the read count of the associated perfectly matched target to generate relative scores for cleavage of ‘off-target’ substrates.

### Purification of IscB proteins

To purify OrufIscB, OrufIscB-REC and NovaIscB proteins, human codon-optimized IscB proteins were cloned into a pET45b(+) backbone with an N-terminal His14-Twin-strep-bdSUMO tag. These plasmids were transformed into BL21(DE3) competent cells (Thermo Fisher Scientific). Cells were grown at 37 °C in terrific broth (TB) medium supplemented with 100 μg ml^−1^ ampicillin. Once the culture reached an optical density of approximately 0.6, the culture was shifted to 18 °C and supplemented with 0.2 mM isopropyl β-d-1thiogalactopyranoside (IPTG) for overnight induction at 18 °C. The pellet was collected by centrifugation and resuspended in the lysis buffer (50 mM Tris (pH 8), 1 M NaCl, 5 mM MgCl_2_, 5% glycerol, 40 mM imidazole and 5 mM β-mercaptoethanol) with PMSF protease inhibitors. The pellets were lysed by passing twice through an LM20 Microfluidizer (Microfluidics) at 28,000 psi. The soluble fraction was collected after centrifugation at 15,060 × *g* for 30 min, then bound to Ni-NTA Agarose (Qiagen). The Ni beads were washed first with 12 column volumes (CV) of lysis buffer, then 5 CV of high-salt buffer (50 mM Tris (pH 8), 2 M NaCl, 5% glycerol, 5 mM MgCl_2_, 40 mM imidazole and 5 mM β-mercaptoethanol) and 5 CV of low-salt buffer (50 mM Tris (pH 8), 500 mM NaCl, 5% glycerol, 5 mM MgCl_2_, 40 mM imidazole and 5 mM β-mercaptoethanol) in turn. The proteins were eluted with elution buffer (50 mM Tris (pH 8), 500 mM NaCl, 5% glycerol, 5 mM MgCl_2_, 300 mM imidazole and 5 mM β-mercaptoethanol), cleaved using bdSENP1 protease to remove the N-terminal tag and then dialyzed overnight in dialysis buffer (50 mM Tris (pH 8), 500 mM NaCl, 5% glycerol, 5 mM MgCl_2_, and 5 mM β-mercaptoethanol). The proteins were concentrated, aliquoted and stored at −80 °C.

### In vitro cleavage assay with purified proteins

For the in vitro cleavage assays, the labeled double-stranded DNA substrates were generated by PCR amplification of pUC19 plasmids containing the target and TAM sequences using Cy5 and DyLight800-conjugated DNA oligonucleotides (IDT) as primers as described in Cell-Free Transcription–Translation Cleavage Assays. All ωRNAs used in this assay were in vitro transcribed using the same protocol described in the RNA transfection protocol described in Mammalian Genome Editing. Each reaction of the cleavage assay contained 10 nM DNA substrate, 1.2 μM protein and 1.1 μM ωRNA in a final reaction buffer of 20 mM HEPES (pH 7.5), 50 mM NaCl and 5 mM MgCl_2_. Reactions were incubated at 42 °C for 1 h, followed by RNase A treatment (Qiagen) and proteinase K treatment (NEB). DNA was then purified with QIAquick spin columns (Qiagen), resolved by gel electrophoresis on E-gels (Thermo Fisher Scientific) and imaged on a BioRad Chemidoc imager.

### TTISS

TTISS assays were performed as described^12^ with minor modifications as follows. Briefly, donor oligos (5′ - /5phos/G*T*TGTGAGCAAGGGCGAGGAGGATAACGCCTCTCTCCCAGCGACT*A*T - 3′ and 5′- /5phos/A*T*AGTCGCTGGGAGAGAGGCGTTATCCTCCTCGCCCTTGCTCACA*A*C - 3′, where * represents phosphothioate backbone modification) were annealed in nuclease-free duplex buffer (30 mM HEPES (pH 7.5), 100 mM potassium acetate) at a final concentration of 10 µM by incubating for 5 s at 95 °C and ramping down at 0.1 °C s^−1^ to 4 °C. HEK293FT cells were transfected in 12-well plates using GeneJuice (MilliporeSigma) as per the manufacturer’s instructions with 1 µg protein expression plasmid, 2 µg combined omegaRNA expression plasmids and 1.5 µg annealed donor oligos. Subsequently, 72 h after transfection, each well was washed with 1 ml Dulbecco’s PBS (MilliporeSigma) and dry trypsinized with 200 µl TrypLE (Thermo Fisher Scientific). Cells were resuspended in 1 ml PBS and centrifuged at 300 × *g* for 5 min at 4 °C to pellet. The pellet was resuspended in 200 µl PBS and used as input to the Qiagen DNEasy Blood & Tissue kit (Qiagen) to extract genomic DNA. Then, 2 µg of purified genomic DNA from each sample was mixed with 20 µl of purified Tn5 enzyme loaded with a Tn5 adaptor (5′-CTGTCTCTTATACACATCTCCGAGCCCACGAGAC-3′) and 1× TAPS buffer (10 mM TAPS, 1 mM MgCl_2_) and incubated at 55 °C for 10 min. Tagmented samples were purified using a QiaQuick DNA purification kit (Qiagen) and amplified twice using KOD Hot Start 2× PCR Master Mix (MilliporeSigma) with primers 5′-GTCGCTGGGAGAGAGGCGTTATC-3′ and 5′-GTCTCGTGGGCTCGGAGATGTGTATAAGAGACAG-3′ with 12 cycles and an annealing temperature of 60 °C in the first round of PCR, then with primers 5′-AATGATACGGCGACCACCGAGATCTACACTATAGCCTACACTCTTTCCCTACACGACGCTCTTCCGATCTTTATCCTCCTCGCCCTTGCTCAC-3′ and 5′-CAAGCAGAAGACGGCATACGAGATNNNNNNNNGTCTCGTGGGCTCGGAGATGTGT-3′ (NNNNNNNN refers to the barcode sequence) with 18 cycles and an annealing temperature of 65 °C in the second round of the PCR. Libraries were sequenced on an Illumina NextSeq. Within each experiment, the resulting FASTQ files were randomly downsampled so each sample had the same number of reads, for direct comparison. Across all experiments, at least 10 million and up to 50 million reads per sample were used for analysis. Reads were mapped using BrowserGenome.org (ref. ^51^). Off-targets for each guide were counted using a custom Python script, allowing for up to 7 mismatches in the guide sequence for 20-nt guides or 5 mismatches for 16-nt and 14-nt guides, and 1 mismatch in the TAM regardless of length. Libraries for associated on-target indel quantification were generated using the purified genomic DNA as described in Mammalian Genome Editing. For each variant, the specificity values (the percentages of total TTISS reads corresponding to detected off-targets) and activity values (the average indel fold changes of each variant versus WT OrufIscB across all guides included in each experiment) were plotted.

### Mammalian base editing assays

Constructs expressing base editors and associated guide RNAs were transfected, genomic DNA was collected and libraries were prepared as described for individual protein–guide combinations in Mammalian Cell Culture and Transfection and Mammalian Genome Editing above. Editing was quantified by counting the number of reads at which the expected edited position in the amplicon was called as a G (on the top strand) or C (on the bottom strand) and dividing by the total number of reads in the sample using a previously described custom Python script^50^. Unless otherwise noted, all reported data are the average of four biological replicates.

### Assessment of OMEGAoff by qPCR

Constructs expressing OMEGAoff or CRISPRoff proteins and associated guide RNAs were transfected as described for individual protein–guide combinations in Mammalian Cell Culture and Transfection and Mammalian Genome Editing. RNA was extracted after 5 days unless otherwise specified using an RNeasy 96 Plus kit (Qiagen) and reverse transcribed using a RevertAid First Strand cDNA Synthesis Kit (Thermo Fisher Scientific) using random hexamer primers as previously described^52^. RNA expression was measured by qPCR using commercially available TaqMan probes (Thermo Fisher Scientific) on a LightCycler 480 II (Roche) with GAPDH as an endogenous internal control in 5-μl multiplexed reactions. Each of the four biological replicates is the average of four technical qPCR replicates, and relative expression was calculated using the double delta Ct (ddCt) method^53^ with a negative control condition (average of all nontargeting replicates) consisting of the corresponding OMEGAoff or CRISPRoffv2.1 expression plasmid co-transfected with an *AAVS1* targeting guide and *GAPDH* as the endogenous control. Statistical significance was assessed using a two-tailed *t*-test. All qPCR quantification data are available in Supplementary Table 7.

For gene activation experiments, we transfected 133 ng dOrufIscB-KRK–VPR of dCas9–VPR plasmids with 66-ng guide plasmids into 96-well plates of HEK293FT cells. RNA was extracted 5 days after transfection, then reverse transcribed and measured by qCPR as discussed above.

### Western blot of PCSK9 protein after OMEGAoff repression

To perform a western blot to detect PCSK9 protein levels in AML12 cells after AAV transduction, cells were plated 16–20 h before infection to ensure 90% confluency at the time of transduction. For 12-well plates to be infected, cells were plated at 200,000 cells per well. Different AAV amounts (5 μl, 10 μl, 20 μl and 40 μl) were added. Then, the cells were transferred into 6-well plates after 2 days and collected 7 days after infection as follows. For each well, cells were washed with cold PBS and incubated for 30 min with 300 μl cold RIPA lysis buffer (Thermo Fisher Scientific) containing 3 μl Halt Protease Inhibitor Cocktail (Thermo Fisher Scientific) and 3 μl 0.5 M EDTA (Thermo Fisher Scientific). Samples were then prepared using LDS sample buffer (Thermo Fisher Scientific) and run on a 4–12% Bolt Bis–Tris gel (Thermo Fisher Scientific) in MOPS buffer for 30 min at 200 V. Membrane transfer was performed using iBlot2 Transfer Stacks (Thermo Fisher Scientific) on an iBlot2 machine for 7 min at 20 V. The membrane was blocked for 1 h at room temperature in 5% nonfat dry milk in TBS with 0.05% Tween-20 (TBS-T) buffer, then incubated with 1:1,000 Anti-PCSK9 antibody (Abcam, ab185194) and 1:10,000 Monoclonal Anti-β-Actin antibody (Sigma-Aldrich, A2228) in 2% nonfat dry milk in TBS-T buffer overnight. The membrane was then washed 5× in TBS-T buffer for 5 min each at room temperature, then incubated for an additional 2 h with secondary antibodies (Cell Signaling Technology, 7074S and 7076P2) at a 1:20,000 dilution in 2% nonfat dry milk in TBS-T buffer. The membrane was then washed an additional 3× as above and imaged on a BioRad Chemidoc imager.

### RNA-seq

HEK293FT cells were transfected with OMEGAoff or CRISPRoff with nontargeting guides, *CLTA*-targeting guides and *CALD1*-targeting guides in 12-well plates. A total amount of 1.8 μg of plasmids was transfected per well including 1.2 μg OMEGAoff or CRISPRoff plasmid and 0.6 μg guide RNA plasmid. Total RNA was extracted using a Direct-zol RNA MiniPrep (Zymo) kit 14 days after transfection. The TruSeq Stranded mRNA Library Preparation Kit (Illumina) was used to prepare RNA-seq library samples starting with 1,000 ng RNA for each sample as per the manufacturer’s protocol. The library was quantified using a Qubit dsDNA HS Assay Kit (Thermo Fisher Scientific) and KAPA Library Quantification Kit (Roche). The final library was sequenced with read 1 50 cycles, index 1 6 cycles and index 2 6 cycles on an Illumina NextSeq. Spliced Transcripts Alignment to a Reference (STAR)^54,55^ was used to align the sequencing reads to the human genome (GRCh37), and Salmon^54^ was used to calculate the normalized transcripts per million (TPM) of each transcript. The transcripts whose TPM values were 0 in any one replicate and those showing more than twofold of difference between the two replicates were filtered out. The differentially expressed genes were identified using DESeq2 (ref. ^56^) by the comparison of *CLTA*-targeting or *CALD1*-targeting samples to nontargeting samples. The differential expressed transcripts (−log_10_(*P* value) > 5.5, log_2_(fold change) < −1 or >1) were labeled. The significant *P* value was determined by Bonferroni correction.

### AAV production

For AAV viral production, HEK293FT cells were used and maintained as described in Mammalian Cell Culture and Transfection. Five 15-cm plates were used per virus prep. For each prep, 60 μg adenoviral helper plasmid, 50 μg pAAV8 serotype packaging (AAV2/8) plasmid and 30 μg transgene plasmid carrying both ωRNA and OMEGAoff protein construct were added to 5 ml OptiMEM with 500 μl 1 mg ml^−1^ PEI max solution (Polysciences). After mixing by vortexing, the mixture was incubated at room temperature for 5–10 min. Then, 1 ml of the transfection mixture was added to each plate dropwise immediately after mixing by pipetting to ensure distribution across the plates. Subsequently, 4 days after transfection, media were collected by PEG precipitation to isolate the particles as follows. Briefly, a solution containing 40% PEG 8000 (Promega) and 2.5 M NaCl was added into the medium at a ratio of 1:4. The mixture was incubated on ice for at least 2 h on a rocker, then centrifuged at 3,000 × *g* for 30 min. The large white pellet was then suspended in PBS and treated with 50 μl 100 mM MgCl_2_ and 50 μl 10 mg ml^−1^ DNAse for 60 min incubation at 37 °C. The solution was then subjected to an iodixanol gradient, and the virus-containing fractions were identified by qPCR and combined. Zeba Spin Desalting columns (Thermo Fisher Scientific) were used to purify and concentrate the viral particles. The titers of AAVs were determined by qPCR using ITR (inverted terminal repeat) primers (F: 5′-AACATGCTACGCAGAGAGGGAGTGG-3′, R: 5′-CATGAGACAAGGAACCCCTAGTGATGGAG-3′) with Roche Lightcycler 480.

### In vivo experiments

All mice used for in vivo experiments were maintained at the vivarium facility of the Broad Institute with a standard diet, light cycles, temperature and humidity conditions. All the experiments were conducted on 5–6-week-old male C57/BL6 mice (The Jackson Laboratory) following IACUC (Institutional Animal Care and Use Committee)-approved protocols. The animals were made to fast for 12 h, and blood was collected through saphenous vein bleeds to measure the serum PCSK9 and total cholesterol levels. Each time, no more than 100 μl blood was collected. Twice, blood collection was performed before injection to evaluate pre-injection PCSK9 and cholesterol levels. After injection, blood collection was conducted once a week starting from 2 weeks postinjection. The AAV vectors, including *Rosa26* targeting and two *Pcsk9*-targeting ωRNAs, were intravitreally injected into mice at a dosage of 2 × 10^11^ total viral particles per animal. The injected volume was adjusted to 100 μl with sterile PBS. A PBS injection was included as a negative control. Five mice were injected for each condition. The animals were randomly chosen for each condition. To measure serum PCSK9 and cholesterol, blood samples were centrifuged at 2,000 × *g* for 15 min. The serum was then separated and stored at −20 °C for subsequent analysis. Serum PCSK9 levels were measured by ELISA using the Mouse Proprotein Convertase 9/PCSK9 Quantikine ELISA Kit (R&D Systems) using a 200-fold dilution as per the manufacturer’s instructions. Total cholesterol levels were measured using an Amplex Red Cholesterol Assay Kit (Thermo Fisher Scientific) following the manufacturer’s instructions. For liver function tests, the serum total bilirubin and ALT levels at the 24-week (6-month) time point were measured using a Bilirubin Assay Kit (Sigma-Aldrich) and an ALT Activity Assay Kit (Sigma-Aldrich) following the manufacturer’s instructions. All the assays were performed by BioTek Synergy Neo2 multi-mode reader (Thermo Fisher Scientific).

### Cryo-EM sample preparation and data collection

The purified OrufIscB-REC–swap 49–ωRNA RNP complexes were loaded onto a Superose 6 Increase 10/300 column (Cytiva) equilibrated with a buffer containing 20 mM HEPES (pH 7.5), 150 mM NaCl, 2 mM MgCl_2_ and 4.5 mM TCEP. The fractions of RNP were pooled and concentrated to 5 mg ml^−1^ using Amicon Ultra-15 Centrifugal Filter Unit (50 kDa nominal molecular weight limit, Millipore UFC905024). To reconstitute the ternary complex, the RNP was mixed with double-strand target DNA, which was formed by the annealing of two DNA oligos encoding the target sequence, and incubated at 37 °C for 30 min. Then, 3 μl of ternary complex was applied onto glow-discharged CryoMatrix R1.2/1.3 300-mesh gold holey grids with amorphous alloy film (Zhenjiang Lehua Technology). The grids were blotted for 3 s under 100% humidity at 4 °C and then vitrified by plunging into liquid ethane using a Vitrobot Mark IV (Thermo Fisher Scientific).

The prepared grids were transferred to the EF-Krios (Thermo Fisher Scientific) operating at 300 kV with a GatanK3 imaging system and the data collected at 105,000× nominal magnification. The calibrated pixel size of 0.4125 Å was used for processing. Zero-loss images were taken using an energy filter slit width of 20 eV. Videos were collected using Leginon 3.6 (ref. ^57^). Data were collected at a dose rate of 27.12 e^−^ Å^−2^ s^−1^ with a total exposure of 1.80 s, resulting in an accumulated dose of 48.82 e^−^ Å^−^^2^. Intermediate frames were recorded every 0.05 s for a total of 40 frames per micrograph. A total of 7,580 images were collected at a nominal defocus range of 0.7–2.4 μm. Ice thickness was determined as described in refs. ^58,59^.

### Cryo-EM data processing and model building

Image processing was performed on CryoSPARC v4.2.0 (ref. ^60^) and RELION 4.0 (ref. ^61^). Image stacks were subjected to beam-induced motion correction using MotionCor2.0 (ref. ^62^). Contrast transfer function parameters for each nondose-weighted micrograph were determined by CTFFIND4 (ref. ^63^). On-the-fly particle picking was done by Warp^64^. Automated particle picking yielded 2,701,471 particles, which were extracted on a binned dataset with a pixel size of 1.65 Å and were subjected to reference-free two-dimensional classification and 8 rounds of heterogeneous refinement, producing 258,164 particles with well-defined structural features of a ternary complex. These particles were re-extracted with a pixel size of 0.825 Å and subjected to nonuniform refinement^65^, which generated a map with an indicated global resolution of 2.58 Å at a Fourier shell correlation of 0.143. The particles were subjected to three-dimensional classification, a subset with 68,817 particles showing features of the inserted REC domain, an extended guide–DNA heteroduplex and an HNH domain. These particles were then subjected to nonuniform refinement, generating a map with an indicated global resolution of 2.71 Å at a Fourier shell correlation of 0.143.

Protein models predicted by AlphaFold2 (refs. ^27,46^) and an ωRNA model of OrufIscB–ωRNA–target DNA complex (Protein Data Bank: 7XHT) were used as initial models. The models were docked into the cryo-EM density maps using ChimeraX 1.7 (ref. ^66^), followed by iterative manual adjustment and rebuilding in ISOLDE^67^ and Coot 0.8.9 (ref. ^68^), against the cryo-EM density. Real space refinements were performed using PHENIX 1.18 (ref. ^69^). The model statistics were validated using MolProbity 4.5 (ref. ^70^). The refinement statistics are provided in Supplementary Table 6. Structural figures were prepared in ChimeraX 1.7.

### Selection and in silico testing of REC swaps

AlphaFold2 was used to create protein structural models of representative IscBs and type II-D Cas9s. Structures were then aligned along the bridge helix and RuvC-II region using PyMol’s super align algorithm to identify regions of homology that may exist near REC insertions. Additional type II-D Cas9 and IscB sequences were retrieved using an HMM search (HMMER) against all protein coding sequences (clustered at 100% sequence identity using MMSeqs2) from the genomic or metagenomic database as described in a previous study^28^. Using MAFFT, the retrieved IscBs and early type II-D Cas9s were aligned, and all regions with REC-like insertions between the bridge helix and RuvC-II domains were selected. The regions corresponding to the structurally homologous regions were then identified and checked for sequence conservation. The positions of conserved residues near the bridge helix and the RuvC-II were used as anchor positions for recombining sequences. Proteins without homologous sequences at the anchor positions were not considered for REC swaps. REC insertions from other orthologs were then swapped into the OrufIscB ortholog by switching the region between the conserved residues. For a select set of divergent Cas9s with crystal structures, structural homology alone was used to select potential REC swaps with anchor points in similar regions as described above. However, because most Cas9s outside of type II-D do not have the conserved charged motif found after the bridge helix in most IscBs, the RECs in these cases were truncated at the N-terminus in a structurally similar location to maintain the overall folding of the REC domain. AlphaFold2 models of OrufIscB along with swapped REC domains were performed as described above for the native orthologs.

The sequence conservation Shannon information mapping was performed as follows. All type II-D Cas9s with REC domains were aligned using MAFFT along with a panel of IscBs, including OrufIscB. This alignment was then trimmed to match the REC domain boundaries of Nba-1 REC, which was then set as a reference for the alignment. For each position in the reference sequence of Nba-1 REC, the distribution of nongap sequences was determined, and the Shannon information was calculated for this distribution (using log base 2). High Shannon information positions indicate high conservation in the structure and were used to determine conserved residues for REC loop swaps for regions 1, 2 and 3. For each region (1, 2 and 3), swaps were created by exchanging the OrufIscB sequence within the conserved flanking residues with sequences from another REC ortholog, based on the multiple sequence alignment.

For Fig. 2a, AlphaFold3 (ref. ^71^) was used to generate a full RNP prediction with target DNA, nontarget DNA, RNA and protein. However, due to the low quality of the RNA prediction, only the guide portion of the RNA was shown. AlphaFold3 (ref. ^71^) models were generated for the WT OrufIscB system, OrufIscB-REC system and NovaIscB system.

### RNA model

The secondary structure model of the OrufIscB ωRNA was generated by the RNAstructure webserver^72^ on the OrufIscB ωRNA scaffold. The prediction contained a spurious set of two base pairs between 69 and 141 as well as 70 and 140, which were removed, as positions 69 and 70 are thought to separate the adaptor hairpin and the large pseudoknot stemloop and are thus unlikely to form additional stemloop contacts in the structure.

### Calculating the distribution of potential guides of OrufIscB within 500 bp of transcription start sites

Human transcription factor start sites were downloaded from refTSS_v4 (ref. ^73^) and mapped onto the human genome (version GRCh38.110). As refTSS_v4 may contain multiple transcription start sites for the same mRNA or gene, only transcription start sites up to 100 bp into the predicted mRNA start site or upstream from the predicted mRNA start site were selected. Of these remaining transcription start sites, the one selected to correspond to a specific gene was the one with the closest distance to the gene. These resulting transcription start sites formed the processed refTSS_v4 dataset. Coding sequences (CDSs) were identified within each genomic chromosome (ignoring the mitochondrial genome) based on the GenBank annotation, along with its corresponding predicted mRNA. For each gene’s mRNA, the standard gene name was used to cross-reference with the refTSS_v4 database to identify the transcription start site, using the predicted mRNA start site from the human genome GenBank file as the default transcription start site if there was no corresponding entry in the processed refTSS_v4. For each gene with a CDS, a ±500 bp window was formed around the transcription start site and searched for NTAAA or TTTAN (reverse complement of NTAAA) sequences to identify the number of potential guides and target sites within a 500-bp window around the transcription start site. The distribution of the number of potential guides (within 500 bp of the transcription start site) was then calculated across all CDS-containing genes.

The same approach was used as above for calculating the number of genes available for NovaIscB for knockout (KO). This was done by taking each gene’s genome-mapped coding sequence segments and counting the number of TAM sites that would enable the guide to have position 10 of the guide inside the coding sequence. Position 10 was used as this results in guides in which the cleavage site will be located inside the CDS. The distribution of the number of theoretical guides that could target a given gene was then calculated across all CDSs.

### Reporting summary

Further information on research design is available in the Nature Portfolio Reporting Summary linked to this article.

## Online content

Any methods, additional references, Nature Portfolio reporting summaries, source data, extended data, supplementary information, acknowledgements, peer review information; details of author contributions and competing interests; and statements of data and code availability are available at 10.1038/s41587-025-02655-3.

## Supplementary information

Supplementary Information Captions for Extended Data Figs. 1–10, Supplementary Figs. 1 and 2, and Supplementary Table 6.

Reporting Summary

Supplementary Table 1 Summary of IscB orthologs, related to Fig. 1. Sheet 1 contains all main IscBs and II-D Cas9 orthologs tested. Sheet 2 contains a small panel of extra CasIscBs that were additionally tested (beyond TbaIscB) but not included in the main diversity survey.

Supplementary Table 2 Summary of indel percentages for all the IscB variants, related to Figs. 1–4. The sheet names refer to the figures corresponding to the data contained in the sheet.

Supplementary Table 3 Summary of all the tested REC domains and library design for REC screening, related to Fig. 2. This table contains the amino acid sequences of the tested REC insertions.

Supplementary Table 4 Summary of all the tested REC loop swappings, related to Fig. 2. Unnamed ortholog IDs follow the convention of genome_accession && contig_accession && start_end_strand, where start, end and strand are the start coordinate, end coordinate and DNA strand that the protein occurs on in the contig.

Supplementary Table 5 Off-target sites detected by TTISS, related to Fig. 3b,c,e and Supplementary Fig. 8g,i.

Supplementary Table 7 Summary of all the results for genome and epigenome modification by engineered OrufIscB, related to Fig. 4. Sheet 1 contains target A-to-G editing efficiencies by OrufIscB–KRK fused to the ABE8e system. Sheet 2 contains target gene repression data by OMEGAoff and CRISPRoff. Sheet 3 contains the results of AAV-delivered OMEGAoff in mice.

Supplementary Table 8 Primers used for sequencing in this study, related to all the figures.

## Source data

Source Data Fig. 2 Uncropped gels.

Source Data Fig. 4 Uncropped gels.

Source Data Fig. 5 Uncropped gels.

Source Data Extended Data Fig. 4 Uncropped gels.

Source Data Extended Data Fig. 6 Uncropped gels.

## Data Availability

Data supporting the findings of this study are available in the Article and Supplementary Information. Primer information is provided in Supplementary Table 8. Plasmids are available on Addgene. The PDB structure 7XHT is available on RCSB PDB. The OrufIscB-REC–swap 49 structure is available on RCSB PDB under accession 9NVU, with associated cryo-EM data available on EMDB under accession EMD-49856. Source data are provided with this paper.

## Code availability

All described custom scripts and analyses are available via Zenodo at 10.5281/ZENODO.15058686 (ref. ^74^). Other code used in this publication is described in previously published work, as described in Methods.
