# Supplementary material for: Evolution-guided protein design of IscB for persistent epigenome editing in vivo
Source: Nat Biotechnol. 2025 May 7;44(5):759–70. doi: 10.1038/s41587-025-02655-3 (PMC13180657; doi:10.1038/s41587-025-02655-3)
Supplement: Supplementary file 1 — Captions for Extended Data Figs. 1–10, Supplementary Figs. 1 and 2, and Supplementary Table 6. [file 41587_2025_2655_MOESM1_ESM.pdf]

nature biotechnology

Supplementary information https://doi.org/10.1038/s41587-025-02655-3

Evolution-guided protein design of IscB for persistent epigenome editing in vivo

In the format provided by the authors and unedited

Supplemental Information

Evolution-guided protein design of IscB for persistent epigenome editing in vivo

Authors: Soumya Kannan[1,2,3,4,5]†, Han Altae-Tran[1,2,3,4,5]†, Shiyou Zhu[1,2,3,4,5]†, Peiyu Xu[1,2,3,4,5]‡, Daniel Strebinger[1,2,3,4,5]‡, Rachel Oshiro[1,2,3,4,5]‡, Guilhem Faure[1,2,3,4,5], Lukas Moeller[1,2,3,4,5], Julie Pham[1,2,3,4,5], Kepler S. Mears[1,2,3,4,5], Heyuan M. Ni[5], Rhiannon K. Macrae[1,2,3,4,5] & Feng Zhang[1,2,3,4,5]*

Supplemental Information File:

Captions for Extended Data Figures 1-10

Supplementary Figures 1-2

Supplementary Table 6

Additional Supplemental Items:

Supplementary Tables 1 – 5, 7, 8 (provided as separate Excel files)

Extended Data Figure 1. Ortholog screening of IscB family, related to Figure 1.

(**A**) Experimental traversal of the IscB phylogenetic tree to identify IscB orthologs active for human genome editing. Initial set of 6 tested IscBs shown on left. Set 1 of phylogenetically diverse IscBs/II-D Cas9s shown in middle. Set 2 of mainly larger IscBs with potential REC inserts shown on right.

(**B**) TAMs of active IscB proteins determined by in vitro cleavage assays. All the protein sequences and accession of source contigs are listed in Table S1.

(**C**) TAM wheel representation of OrufIscB TAM. All motifs adding up to 5% or more of cleaved TAMs are listed to the right. Constituents of each relevant motif are labeled around the circumference of the TAM wheel.

(**D**) Schematic of the ortholog screening in HEK293FT cells (results shown in Figure 1C).

(**E**) Zoomed in y-axis representation of Indel formation mediated by TbaIscB with individual guides in HEK293FT cells displayed in Figure 1D. Data are presented as mean +/- SD, n=4 replicate transfections.

(**F**) Indel formation mediated by WT OrufIscB with individual guides in HEK293FT cells. Data are presented as mean +/- SD, n=4 replicate transfections.

Extended Data Figure 2. Sequence and structure comparison of IscBs and II-D Cas9s, related to Figure 1, and evolution of the Cas9 REC domain triple helical bundle, related to Figure 2.

(**A**) Alignment of tested IscBs and early II-D Cas9s in the location between the bridge helix (BH) and RuvC-II regions using MAFFT. The consensus sequence is at the top, followed by the sequence logo. OrufIscB is set as the reference sequence for comparison. The conserved FNNR and LTP motifs flanking REC insertions are shown. Hidden insertions (from less than 2% of loci) are indicated by red jagged lines.

(**B**) Comparison of AlphaFold2 models of OrufIscB, CzcbIscB, and NbaCas9-1 focusing on the REC insertion region between the bridge helix and RuvC-II. The conserved FNNR and LTP sites are shown. The conserved zinc finger is also shown along with the REC lobe of NbaCas9-1. OrufIscB contains a unique beta hairpin REC linker between the FNNR motif and bridge helix.

(**C**) AlphaFold2 models of the REC domain and surrounding regions from two IscBs and two Type II-D Cas9s were superimposed onto the OgeuIscB structure (PDB: 7XHT[16]), using the RuvC-II and bridge helix regions as templates for alignment. The guide:target duplex from OgeuIscB is shown in the final model, but the OgeuIscB protein itself is not shown.

(**D**) Left: Zoom in to the previously described phylogenetic tree[14] focusing on early Cas9 evolution from IscB. Cluster names are shown along the tree. Right: AlphaFold2 models of the

cluster representatives. Early II-D Cas9s contain RECc insertions and RECb insertions into the triple helical REC bundle that emerged quickly after IscB evolved into Cas9.

(**E**) Architecture of the triple helical REC bundle. Top: structure model of a triple helical REC bundle. Bottom: schematic of the triple helical rec bundle. In both cases, arrows point to where 3 different classes of REC domains insert into (RECa, RECb, and RECc).

Extended Data Figure 3. In silico testing of correct folding of OrufIscB REC grafts, related to Figure 2.

AlphaFold2 models of REC domains grafted into OrufIscB. REC domains are colored in blue. The FNNR motif is colored in black, while the LTP motif is colored in brown. The beta hairpin REC linker is colored in orange. The bridge helix is additionally shown, but not colored. OrufIscB + Nba-1 REC, for example, maintains correct folding of the FNNR motif, the beta hairpin REC linker, and the corresponding REC lobe, as well as the REC zinc finger.

Extended Data Figure 4. Experimental validation and screening of REC grafts into OrufIscB, related to Figure 2.

(**A**) IVTT cleavage assay of OrufIscB (WT) with various REC insertions using IVTT protein and WT ωRNA with labeled target (target strand: Cy3; non-target strand: Cy5). Gel was imaged in Cy5 channel only. WT: Wildtype, NTS: non-target strand.

(**B**) Indel formation mediated by WT OrufIscB with various REC insertions for two different guides in HEK293FT cells. Data are presented as mean +/- SD, n=4 replicate transfections.

(**C**) Schematic of the IVTT REC screening assay that tests for cleavage with varying levels of TAM distal mismatches.

(**D**) In vitro cleavage activity score of all the screened REC domains in IVTT REC screening assay.

(**E**) Indel formation mediated by WT OrufIscB and variants with various REC domain insertions in HEK293FT cells. Data are presented as mean +/- SD, n=2 replicate transfections.

(**F**) Indel formation mediated by OrufIscB (WT) vs OrufIscB-REC (OrufIscB + Nba-1 REC) across a larger panel of guides in HEK293FT cells. Data are presented as mean +/- SD, n=4 replicate transfections.

Extended Data Figure 5. Point mutation selection and testing for increasing OrufIscB-REC activity, related to Figure 2.

(**A**) Multiple sequence alignment and weblogo of all tested IscBs and early Cas9 (II-D) against a reference sequence of OrufIscB-REC, including the proposed point mutations for increasing activity of IscB. Most proposed point mutations were selected from the natural amino acid distribution at each position, prioritizing mutations that may improve contacts with the DNA or improve packing of the protein. The first 4 non-OrufIscB-REC sequences are shown as examples in the figure, but the multiple sequence alignment used for this analysis contains all sequences.

(**B**) Indel formation mediated by OrufIscB-REC with each point mutation across 2 target sites. Data are presented as mean +/- SD, n=4 replicate transfections.

(**C**) Indel formation mediated by OrufIscB-REC with single, double, and triple mutation combinations using the top 3 mutations from distant parts of the protein that were hypothesized to combine synergistically across a panel of 12 guides. Data are presented as mean +/- SD, n=4 replicate transfections.

(**D**) Indel formation mediated by OrufIscB-KRK using guide lengths ranging from 12 to 28 nt at two target sites. Data are presented as mean +/- SD, n = 3.

(**E**) Specificity analysis based on the average indel fold changes of OrufIscB-REC or OrufIscB-KRK vs WT OrufIscB and % off-target reads in total from tagmentation-based tag insertion site sequencing (TTISS) using a pool of 20 nt guides with WT OrufIscB, OrufIscB-REC, and OrufIscB-KRK.

Extended Data Figure 6. Structure guided engineering of the REC domain in OrufIscB-REC, related to Figure 2.

(**A**) IVTT cleavage assay of OrufIscB-REC with 12 single loop swaps in the REC domain using IVTT protein and WT ωRNA with labeled target (target strand: Cy3; non-target strand: Cy5). Gel was imaged in Cy5 channel only. NTS: non-target strand.

(**B**) Indel formation mediated by OrufIscB-REC harboring 52 single loop swaps in the REC domain with either a 20-nt ωRNA or a 14-nt ωRNA targeting HPRT1, DYNC1H1 and DYRK1A. The indel ratio (20 nt/14 nt) and indel activity with the 20-nt guide for each variant are plotted. The selected candidates for further combination tests were labeled in shades of red. Double dagger indicates swap 49. Data are presented as mean +/- SD, n=3 replicate transfections.

(**C**) Cryo-EM ternary complex structure of OrufIscB-REC-swap 49 with a native ωRNA and target DNA heteroduplex with a focus on the hydrophobic interactions between the engineered REC domain and the guide:target heteroduplex. Swap 49 in swap region 3 is shown in red.

(**D**) Indel formation mediated by OrufIscB-REC harboring 54 double loop swaps in the REC domain with either a 20-nt ωRNA or a 14-nt ωRNA targeting DYNC1H1. The indel ratio (20 nt/14 nt) and indel activity with the 20-nt guide for each variant are plotted. Variant c12 (red, swap combo 12) was selected as NovaIscB. Data are presented as mean +/- SD, n=3 replicate transfections.

(**E**) In vitro cleavage assay with OrufIscB-REC with swap combo 6 using ωRNAs 11 to 20-nts long.

(**F**) On-target indel formation at 4 target sites mediated by WT OrufIscB, OrufIscB-REC, NovaIscB or OrufIscB-KRK in TTISS samples using 14-nt (top) or 20-nt (bottom) guide pool.

(**G**) Specificity analysis of OrufIscB-REC, NovaIscB, OrufIscB-KRK and WT OrufIscB with 14-nt guides. % off-target reads is the average of the number of off-target reads divided by total number of reads for each guide from TTISS using a pool of four 14-nt guides. Indel fold change is the average of fold change of percent indels generated relative to WT OrufIscB across the same four guides as measured by amplicon sequencing of the same samples. Error bars represent standard deviation. The numbers of detected off-target sites are included in brackets.

(**H**) Specificity analysis of OrufIscB-KRK with 20-nt guides. X and Y axes are calculated as in (G).

(**I**) On-target indel formation at 4 target sites mediated by NovaIscB, enOgeuIscB, eIscB, AsCas12f-YHAM and SpCas9 in TTISS samples using individually transfected guides.

Extended Data Figure 7. Structural analysis of OrufIscB-REC-swap 49-ωRNA-target DNA complex, related to Figure 2.

(**A**) Flow chart of cryoEM data analysis of OrufIscB-REC-swap 49-ωRNA-target DNA complex including example micrographs of the imaged particles (top row), estimated particle clusters (second row).

(**B**) Representative cryo-EM image from 7,580 movies (top) and representative 2D averages (bottom) of the OrufIscB-REC-swap 49-ωRNA-target DNA complex.

(**C**) Cryo-EM density of the OrufIscB-REC-swap 49-ωRNA-target DNA complex. Light blue: OrufIscB-REC-swap 49; green, inserted REC domain; purple: ωRNA; pink, DNA non-target strand; light pink, DNA target strand.

(**D**) EM density and atomic model of the inserted REC domain, HNH domain, the catalytic site of the RuvC domain and the catalytic site of the HNH domain.

Extended Data Figure 8. Structural comparison of OrufIscB-REC-swap 49 to OgeuIscB, related to Figure 2.

(**A**) Structural comparison of OgeuIscB (PDB: 8CTL) and OrufIscB-REC-swap 49 RNPs showing conserved protein domain architecture except the engineered REC lobe in OrufIscB-REC-swap 49.

(**B**) Close-up view of the interaction between REC domain and extended RNA/DNA duplex and the catalytic cleavage mechanisms on HNH domain in OrufIscB-REC-swap 49.

Extended Data Figure 9. Conditional activation switch of OrufIscB's activities, related to Figure 4.

(**A**) IVTT cleavage assay with WT OrufIscB and ωRNAs progressively truncated from the 3′ end at single nucleotide resolution. WT: wild-type; NTS: non-target strand. (Note a subset of this data is shown in Fig. 4B).

(**B**) Indel formation using ωRNAs with combined 5′/3′ truncations plus truncations in the nexus hairpin using the OrufIscB-KRK protein at two target sites. Data are presented as mean +/- SD, n=3 replicate transfections.

(**C**) IVTT cleavage assay with WT OrufIscB and ωRNAs progressively truncated from the 3′ end at single nucleotide resolution with or without the truncated component included as an additional RNA in trans (transRNA). The red circle indicates cleavage with the 35 nt transRNA. WT: wild-type; NTS: non-target strand.

(**D**) Schematic of secondary structure and sequence of OrufIscB ωRNA U149-G205. Red line indicates the sequence truncated and reconstituted in trans as indicated by the red circle in (C).

(**E**) Indel formation in HEK293FT cells by OrufIscB-KRK with full-length ωRNA (blue), or ωRNA with 35 nt truncated from the 3′ end, with (red) or without (gray) the truncated 35 nt sequence added back in trans (transRNA). Data are presented as mean +/- SD, n=4 replicate transfections.

Extended Data Figure 10. Applications of NovaIscB, related to Figure 5.

(**A**) A-to-G conversion efficiency of OrufIscB-KRK-ABE8e at 10 target sites in HEK293FT cells. Data are presented as mean +/- SD, n=4 replicate transfections.

(**B**) Distribution of the number of guides targeting each human CDS for knockdown by OrufIscB/NovaIscB using an NTAAA TAM.

(**C**) Normalized target transcript levels in HEK293FT cells co-transfected with CRISPRoff or OMEGAoff plasmids and negative control (AAVS1 targeting) guides or guides targeting NP63 and PCSK9 as assayed by RT-qPCR. Data are presented as mean +/- SD, n=4 replicate transfections.

(**D**) Normalized target (FABP4) transcript levels in HEK293FT cells transfected with plasmids or in vitro transcribed RNAs encoding ωRNAs and OMEGAoff as assayed by RT-qPCR. Data are presented as mean +/- SD, n=4 replicate transfections.

(**E**) DNA methylation levels at CpG sites in the targeted FABP4 promoter region assayed by bisulfite sequencing (BS-Seq) in HEK293FT cells co-transfected with OMEGAoff plasmids and negative control (AAVS1 targeting) ωRNA or FABP4 targeting ωRNA. Data are presented as

mean +/- SD, n=3 replicate transfections. The amplicon sequence for BS-Seq is included. The guide sequences are labeled as red. The two CpG sites are labeled as blue. Gray shading denotes primer binding regions to make the amplicon.

(**F**) Normalized target transcript levels in HEK293FT cells at time points from 1 to 3 weeks after co-transfection of CRISPRoff or OMEGAoff plasmids and guides targeting FABP4 or ASCL1 relative to an AAVS1 targeting negative control guide as assayed by RT-qPCR. Data are presented as mean +/- SD, n=4 replicate transfections.

(**G**) Normalized target (ASCL1) transcript levels in HEK293FT cells transfected with various plasmid amounts of OMEGAoff or CRISPRoff and associated guide RNAs as assayed by RT-qPCR. Data are presented as mean +/- SD, n=4 replicate transfections.

(**H**) Normalized target transcript levels in HEK293FT cells co-transfected with dCas9-VPR or dOrufIscB-KRK-VPR plasmids and negative control (AAVS1 targeting) guides or guides targeting ASCL1, CA2 or CALD1 as assayed by RT-qPCR. Data are presented as mean +/- SD, n=4 replicate transfections.

(**I**) Quantification of PCSK9 protein levels assayed by western blot in AML12 cells after AAV infection of the OMEGAoff targeting Rosa26 (non-targeting) or Pcsk9. Assays were conducted 1 week post infection with different volumes of viruses as indicated, related to Fig. 5G.

(**J**) RNA-seq analysis of HEK293FT cells co-transfected with OMEGAoff or CRISPRoff plasmids and guides targeting CLTA or CALD1 relative to untransfected. The comparison of target guides vs negative control was conducted by DESeq2 and displayed as volcano plots. The differential expressed transcripts (-Log10P-value > 5.5, Log2 fold change < -1 or > 1) were labeled, n=2 replicate transfections.

(**K**) Liver function tests (total Bilirubin and ALT assays) of serum samples from uninjected, PBS injected, Rosa26-targeting, and Pcsk9-targeting ωRNAs injected mice at the time point of 24 weeks (6 months) after injection. Data are presented as mean +/- SD, n=4 animals.

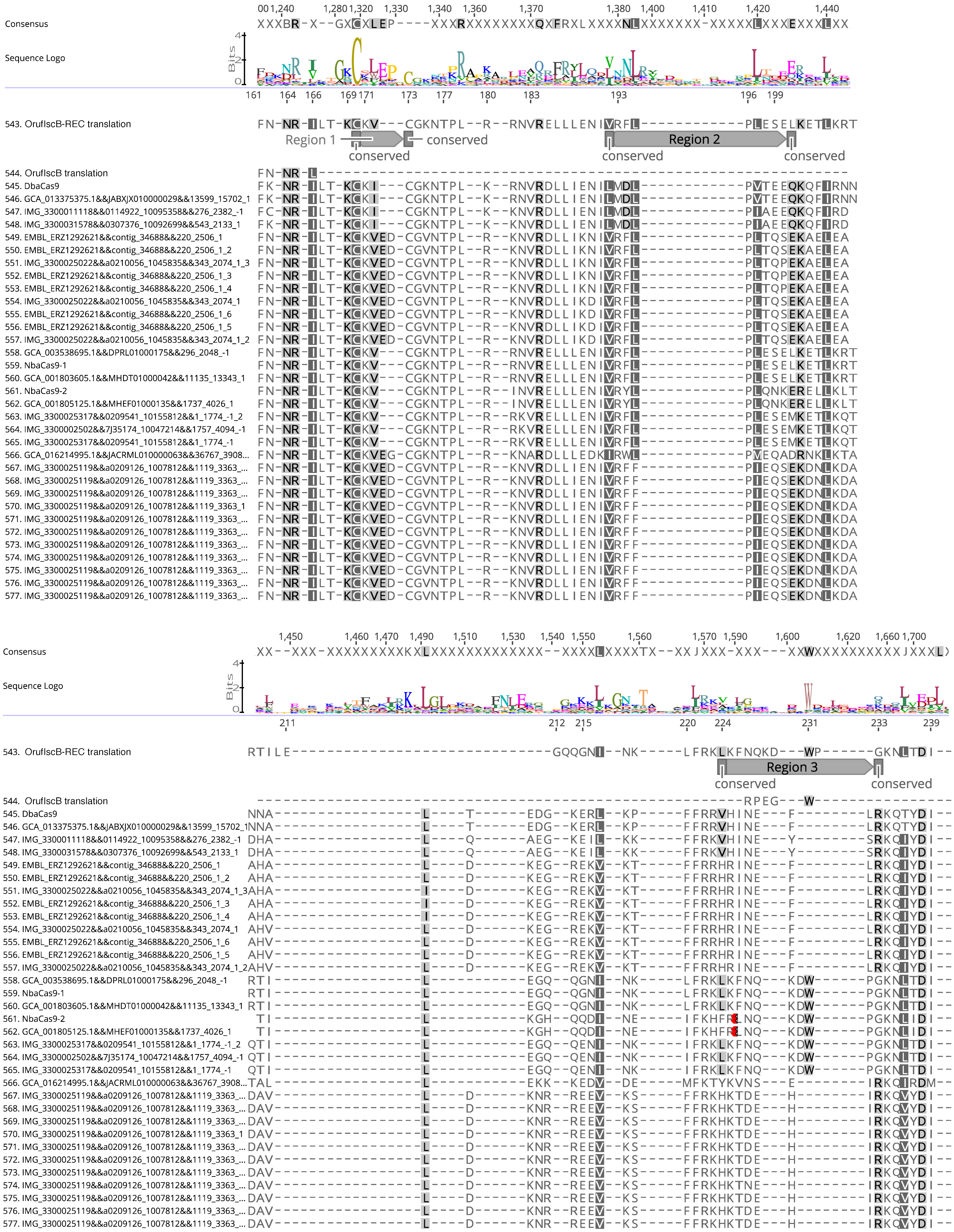

Supplementary Figure 1. Multiple sequence alignment of REC loop swaps, related to Figure 2.

Multiple sequence alignment of various REC domains identified from the early type II-D Cas9 search with example sequences shown. Sequences are referred to by source contig identifiers. OrufIscB-REC is shown as a reference.

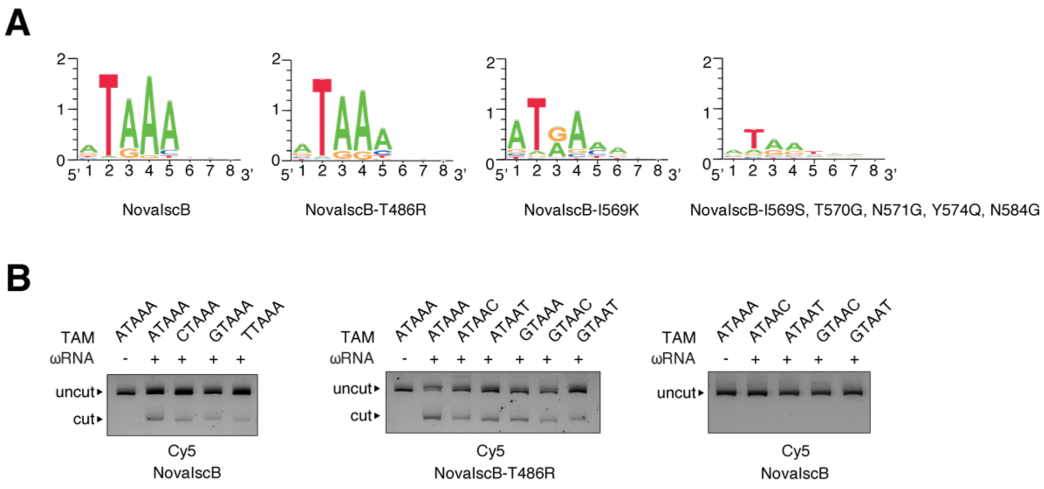

Supplementary Figure 2. TAM engineering of NovaIscB.

(A) TAMs of NovaIscB and NovaIscB with mutants including T486R, I569K, and a combination of I569S, T570G, N571G, Y574Q, N584G determined by IVTT TAM screens.

(B) In vitro cleavage of NovaIscB and NovaIscB-T486R on dsDNA with different 3′ TAM sequences.

Supplementary Table 1. Summary of IscB orthologs, related to Figure 1. Sheet 1 contains all main IscBs and II-D Cas9 orthologs tested. Sheet 2 contains a small panel of extra CasIscBs that were additionally tested (beyond TbaIscB) but not included in the main diversity survey.

Supplementary Table 2. Summary of indels percentages for all the IscB variants, related to Figures 1, 2, 3 and 4. The sheet names refer to the figures corresponding to the data contained in the sheet.

Supplementary Table 3. Summary of all the tested REC domains and library design for REC screening, related to Figure 2. Contains the amino acid sequences of the tested REC insertions.

Supplementary Table 4. Summary of all the tested REC loop swappings, related to Figure 2. Unnamed ortholog ids follow the convention of genome_accession && contig_accession && start_end_strand, where start, end, and strand are the start coordinate, end coordinate, and DNA strand that the protein occurs on in the contig.

Supplementary Table 5. Off-target sites detected by TTISS, related to Figures 3B, 3C, 3E, S8G, S8I

Supplementary Table 6. Cryo-EM data collection, refinement and validation statistics, related to Figure S9.

	**Engineered OrufIscB-omegaRNA-target DNA complex**<br>(EMDB-x)<br>(PDB x)
**Data collection and processing**	
Magnification	130,000
Voltage (kV)	300
Electron exposure (e–/Å^2)	48.82
Defocus range (μm)	-0.8 to -2.2
Pixel size (Å)	0.825
Symmetry imposed	C1
Initial particle images (no.)	1,386,183
Final particle images (no.)	68,817
Map resolution (Å)	2.71
FSC threshold	0.143
Map resolution range (Å)	2.5-5.0
**Refinement**	
Initial model used	AlphaFold2
Model resolution (Å)	
Map sharpening method	DeepEMhancer
Model composition	
Non-hydrogen atoms	16,415
Protein residues	603
Nucleotide residues	228
Ligands	6

B factors (Å^2)	
Protein	80.63
Nucleotide	22.81
Ligand	26.23
R.m.s. deviations	
Bond lengths (Å)	0.013
Bond angles (°)	2.064
Validation	
MolProbity score	1.26
Clashscore	0.3
Poor rotamers (%)	2.84
Ramachandran plot	
Favored (%)	95.67
Allowed (%)	4.33
Disallowed (%)	0.00

Supplementary Table 7. Summary of all the results for genome and epigenome modification by engineered OrufIscB, related to Figure 5. The sheet names refer to the figures corresponding to the data contained in the sheet.

Supplementary Table 8. Primers used for sequencing in this study, related to all the figures.
