# Supplementary material for: Evolution-guided protein design of IscB for persistent epigenome editing in vivo
Source: Nat Biotechnol. 2025 May 7;44(5):759–70. doi: 10.1038/s41587-025-02655-3 (PMC13180657; doi:10.1038/s41587-025-02655-3)
Supplement: Supplementary file 2 — Reporting Summary [file 41587_2025_2655_MOESM2_ESM.pdf]

nature portfolio

Corresponding author(s): Feng Zhang

Last updated by author(s): Mar 18, 2025

Reporting Summary

Nature Portfolio wishes to improve the reproducibility of the work that we publish. This form provides structure for consistency and transparency in reporting. For further information on Nature Portfolio policies, see our Editorial Policies and the Editorial Policy Checklist.

Statistics

For all statistical analyses, confirm that the following items are present in the figure legend, table legend, main text, or Methods section.

n/a	Confirmed	
☐	☒	The exact sample size (*n*) for each experimental group/condition, given as a discrete number and unit of measurement
☐	☒	A statement on whether measurements were taken from distinct samples or whether the same sample was measured repeatedly
☐	☒	The statistical test(s) used AND whether they are one- or two-sided<br>*Only common tests should be described solely by name; describe more complex techniques in the Methods section.*
☒	☐	A description of all covariates tested
☐	☒	A description of any assumptions or corrections, such as tests of normality and adjustment for multiple comparisons
☐	☒	A full description of the statistical parameters including central tendency (e.g. means) or other basic estimates (e.g. regression coefficient) AND variation (e.g. standard deviation) or associated estimates of uncertainty (e.g. confidence intervals)
☐	☒	For null hypothesis testing, the test statistic (e.g. *F*, *t*, *r*) with confidence intervals, effect sizes, degrees of freedom and *P* value noted<br>*Give P values as exact values whenever suitable.*
☒	☐	For Bayesian analysis, information on the choice of priors and Markov chain Monte Carlo settings
☒	☐	For hierarchical and complex designs, identification of the appropriate level for tests and full reporting of outcomes
☒	☐	Estimates of effect sizes (e.g. Cohen's *d*, Pearson's *r*), indicating how they were calculated

Our web collection on statistics for biologists contains articles on many of the points above.

Software and code

Policy information about availability of computer code

Data collection	Illumina MiSeq with system software version 4.1.0 and NextSeq with system software 4.2.0 were used for NGS data collection. BioRad ChemiDoc Image Lab Touch Software version 2.4.0.03 was used for collecting gel images. BioTek Gen5 version 3.02 software was used for plate reader data collection. Lightcycler 480 softare version 1.5.1.62 SP3 was used for qPCR data collection. Leginon 3.6 was used for collecting cryo-EM data.
Data analysis	Publicly available software and platforms: CRISPResso2, Python 3.7, STAR 2.7.9a, Salmon 1.10.1; R packages: DESeq2 1.46.0; Mmseqs2, AlphaFold2.4, Python3.8, MAFFT7, Pymol2, Infernal1.1.5, Clustal Omega 1.2.4, IQ-Tree2, AlphaFold3, HMMER3.4, CryoSPARC v4.2.0, RELION 4.0, MotionCor 2.0, CTFFIND4, Warp 2.0.0dev33, ChimeraX 1.7, ISOLDE 1.6, Coot 0.8.9, PHENIX 1.18, MolProbity 4.5

For manuscripts utilizing custom algorithms or software that are central to the research but not yet described in published literature, software must be made available to editors and reviewers. We strongly encourage code deposition in a community repository (e.g. GitHub). See the Nature Portfolio guidelines for submitting code & software for further information.

Data

Policy information about availability of data

All manuscripts must include a data availability statement. This statement should provide the following information, where applicable:

- Accession codes, unique identifiers, or web links for publicly available datasets
- A description of any restrictions on data availability
- For clinical datasets or third party data, please ensure that the statement adheres to our policy

All data are available in the main text or the supplementary materials. Primer information is in Table S8. Plasmids are available on Addgene. PDB structure 7XHT is available on RCSB PDB. The OrufIscB-REC-swap 49 structure is available on RCSB PDB under accession 9NVU, with associated cryo-EM data available on EMDB under accession EMD-49856.

Research involving human participants, their data, or biological material

Policy information about studies with human participants or human data. See also policy information about sex, gender (identity/presentation), and sexual orientation and race, ethnicity and racism.

Reporting on sex and gender	No human participants were part of this study
Reporting on race, ethnicity, or other socially relevant groupings	No human participants were part of this study
Population characteristics	No human participants were part of this study
Recruitment	No human participants were part of this study
Ethics oversight	No human participants were part of this study

Note that full information on the approval of the study protocol must also be provided in the manuscript.

Field-specific reporting

Please select the one below that is the best fit for your research. If you are not sure, read the appropriate sections before making your selection.

☒ Life sciences ☐ Behavioural & social sciences ☐ Ecological, evolutionary & environmental sciences

For a reference copy of the document with all sections, see nature.com/documents/nr-reporting-summary-flat.pdf

Life sciences study design

All studies must disclose on these points even when the disclosure is negative.

Sample size	Sample sizes were determined based on sample sizes used in the work of our and other groups generating reproducible results for similar experiments (e.g., Ran et al., 2015; Neumann et al., 2024)
Data exclusions	No data was excluded
Replication	For cell-based experiments, experiments were reproduced with cells from different batches/passages, typically N of 3 (details in manuscript). All attempts to reproduce results were successful. For in vivo experiments, full replication of the experiment was not possible due to the duration of the experiment, but multiple replicate samples were assayed and results from all replicates are reported in this study.
Randomization	For cell-based experiments, samples were not randomized. Controls were run alongside experimental conditions to control for experimental conditions and covariates. For in vivo experiments, animals were randomly allocated prior to injections.
Blinding	Investigators were not blinded to group allocation in this study. Blinding was not possible as the same investigators prepared reagents and allocated them to sample groups.

Reporting for specific materials, systems and methods

We require information from authors about some types of materials, experimental systems and methods used in many studies. Here, indicate whether each material, system or method listed is relevant to your study. If you are not sure if a list item applies to your research, read the appropriate section before selecting a response.

Materials & experimental systems

n/a	Involved in the study
☐	☒ Antibodies
☐	☒ Eukaryotic cell lines
☒	☐ Palaeontology and archaeology
☐	☒ Animals and other organisms
☒	☐ Clinical data
☒	☐ Dual use research of concern
☒	☐ Plants

Methods

n/a	Involved in the study
☒	☐ ChIP-seq
☒	☐ Flow cytometry
☒	☐ MRI-based neuroimaging

Antibodies

Antibodies used	Anti-PCSK9 antibody (Abcam, ab185194), Monoclonal Anti-β-Actin antibody AC74 (Sigma Aldrich, A2228), Mouse and Rabbit secondary antibodies :Anti-rabbit IgG, HRP-linked Antibody #7074, Anti-mouse IgG, HRP-linked Antibody #7076 (Cell Signaling Technology, 7074S and 7076P2)
Validation	Anti-PCSK9 antibody (Abcam, ab185194) has been validated by PMID: 37898598 and PMID: 35379885. Anti-β-Actin antibody has been validated by PMID:25757558 and PMID: 32075966.

Eukaryotic cell lines

Policy information about cell lines and Sex and Gender in Research

Cell line source(s)	HEK293FT from Thermo Fisher Scientific, female<br>AML12 cells from ATCC, sex unknown
Authentication	None of the cell lines used were authenticated
Mycoplasma contamination	None of the cell lines used were tested for mycoplasma contamination
Commonly misidentified lines<br>(See ICLAC register)	No commonly misidentified cell lines were used.

Animals and other research organisms

Policy information about studies involving animals; ARRIVE guidelines recommended for reporting animal research, and Sex and Gender in Research

Laboratory animals	5-6 week old C57BL/6 mice
Wild animals	This study did not use wild animals
Reporting on sex	Only male mice were used in this study
Field-collected samples	This study did not involve field-collected animals
Ethics oversight	Broad Institute IACUC

Note that full information on the approval of the study protocol must also be provided in the manuscript.

Plants

Seed stocks	No plants were used in this study
Novel plant genotypes	No plants were used in this study
Authentication	No plants were used in this study
