# Supplementary figures and images for: Evolution-guided protein design of IscB for persistent epigenome editing in vivo
Source: Nat Biotechnol. 2025 May 7;44(5):759–70. doi: 10.1038/s41587-025-02655-3 (PMC13180657; doi:10.1038/s41587-025-02655-3)

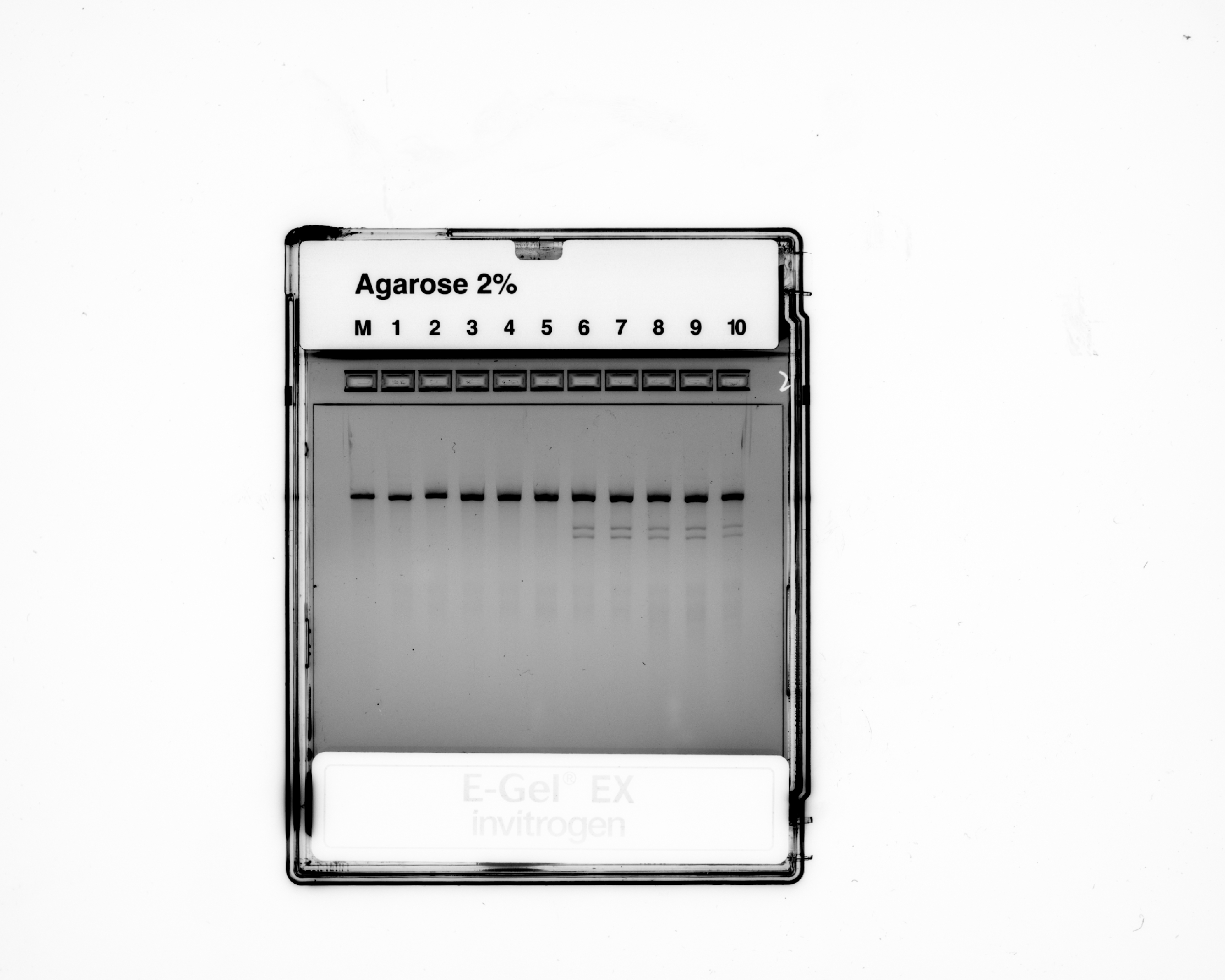

Supplement: Supplementary file 10 — Uncropped gels. [file 41587_2025_2655_MOESM10_ESM.zip › Source Data Fig 2/Figure_2F_NovaIscB.jpg]

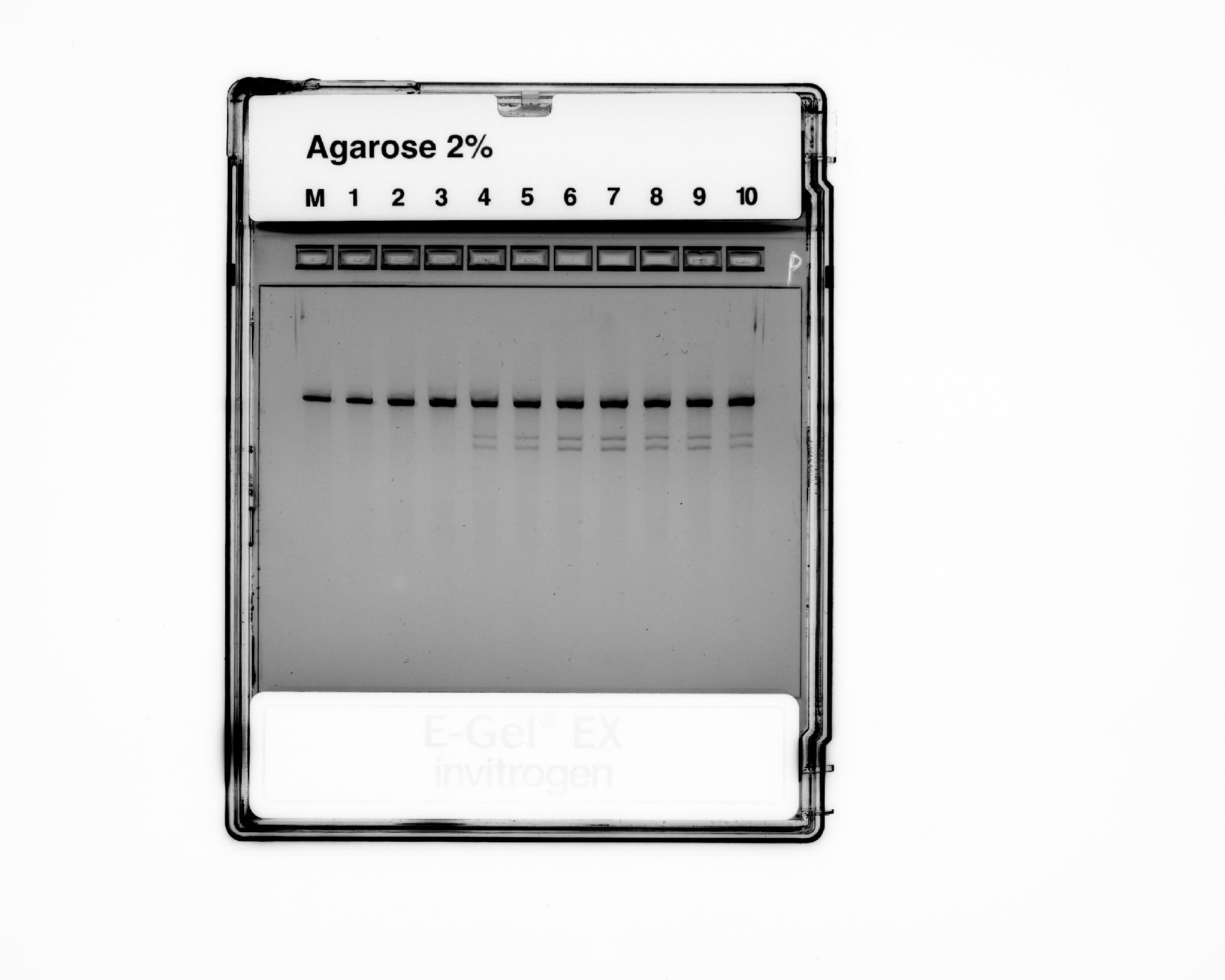

Supplement: Supplementary file 10 — Uncropped gels. [file 41587_2025_2655_MOESM10_ESM.zip › Source Data Fig 2/Figure_2F_OrufIscB-REC.jpg]

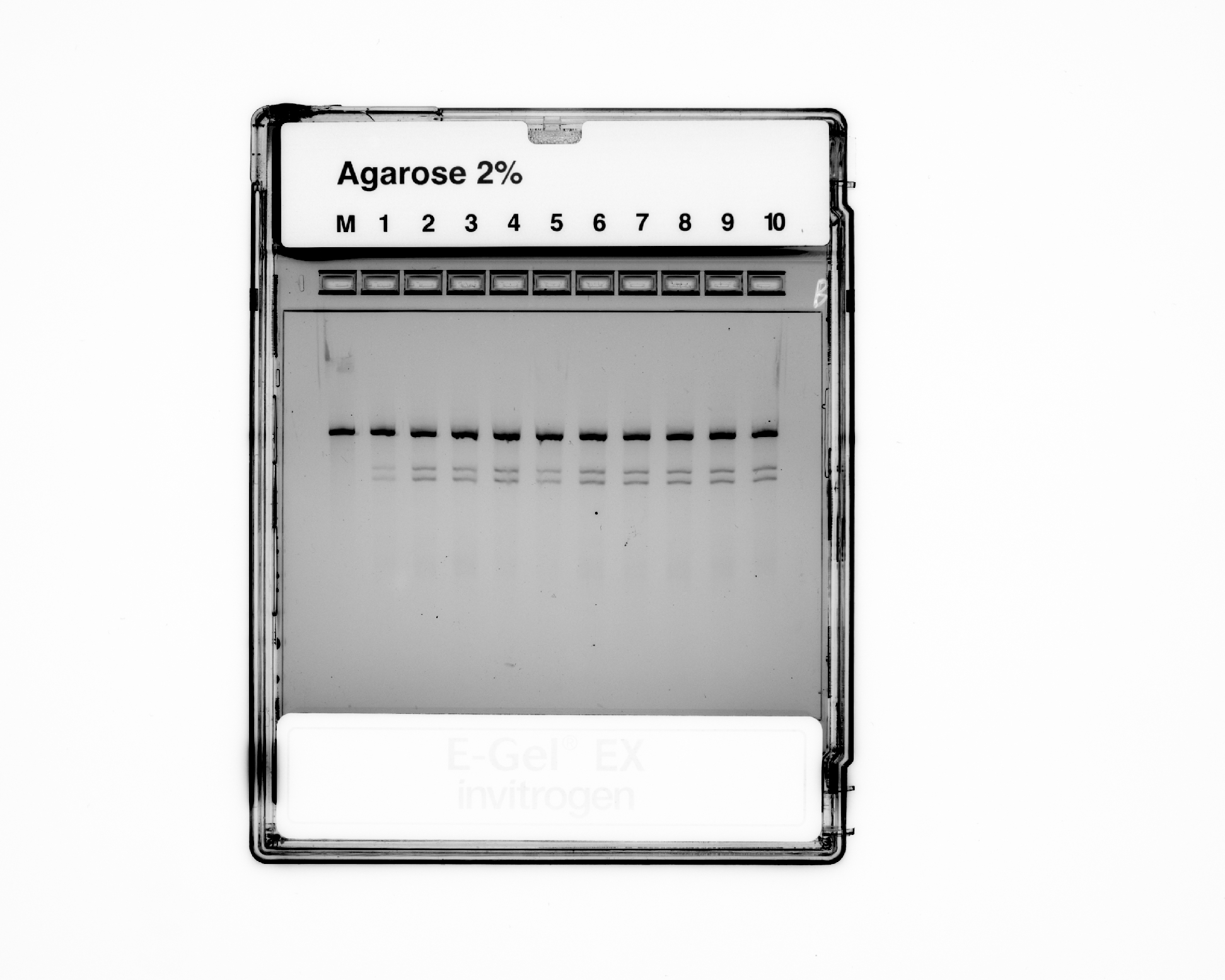

Supplement: Supplementary file 10 — Uncropped gels. [file 41587_2025_2655_MOESM10_ESM.zip › Source Data Fig 2/Figure_2F_OrufIscB.jpg]

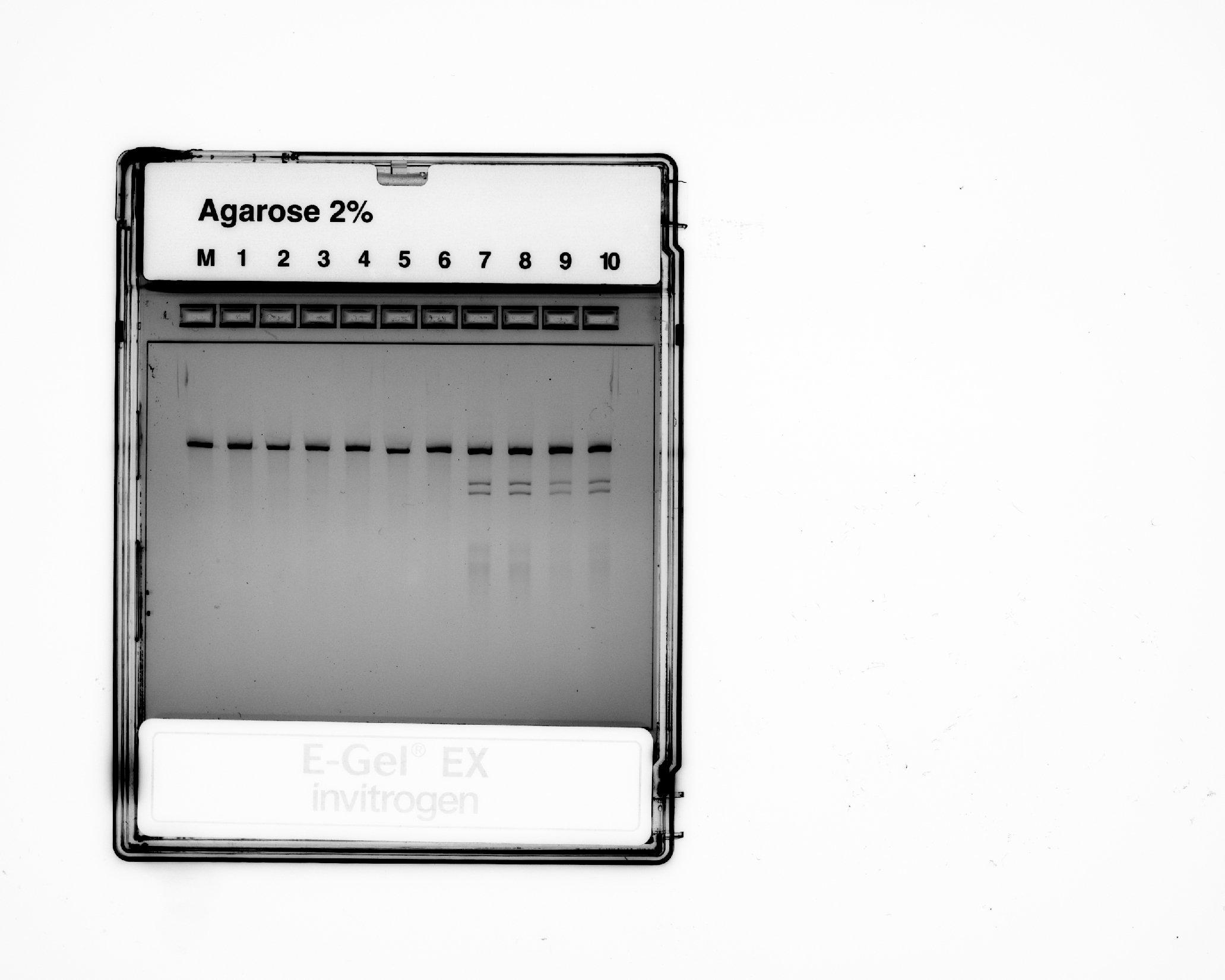

Supplement: Supplementary file 10 — Uncropped gels. [file 41587_2025_2655_MOESM10_ESM.zip › Source Data Fig 2/Figure_2F_SpCas9.jpg]

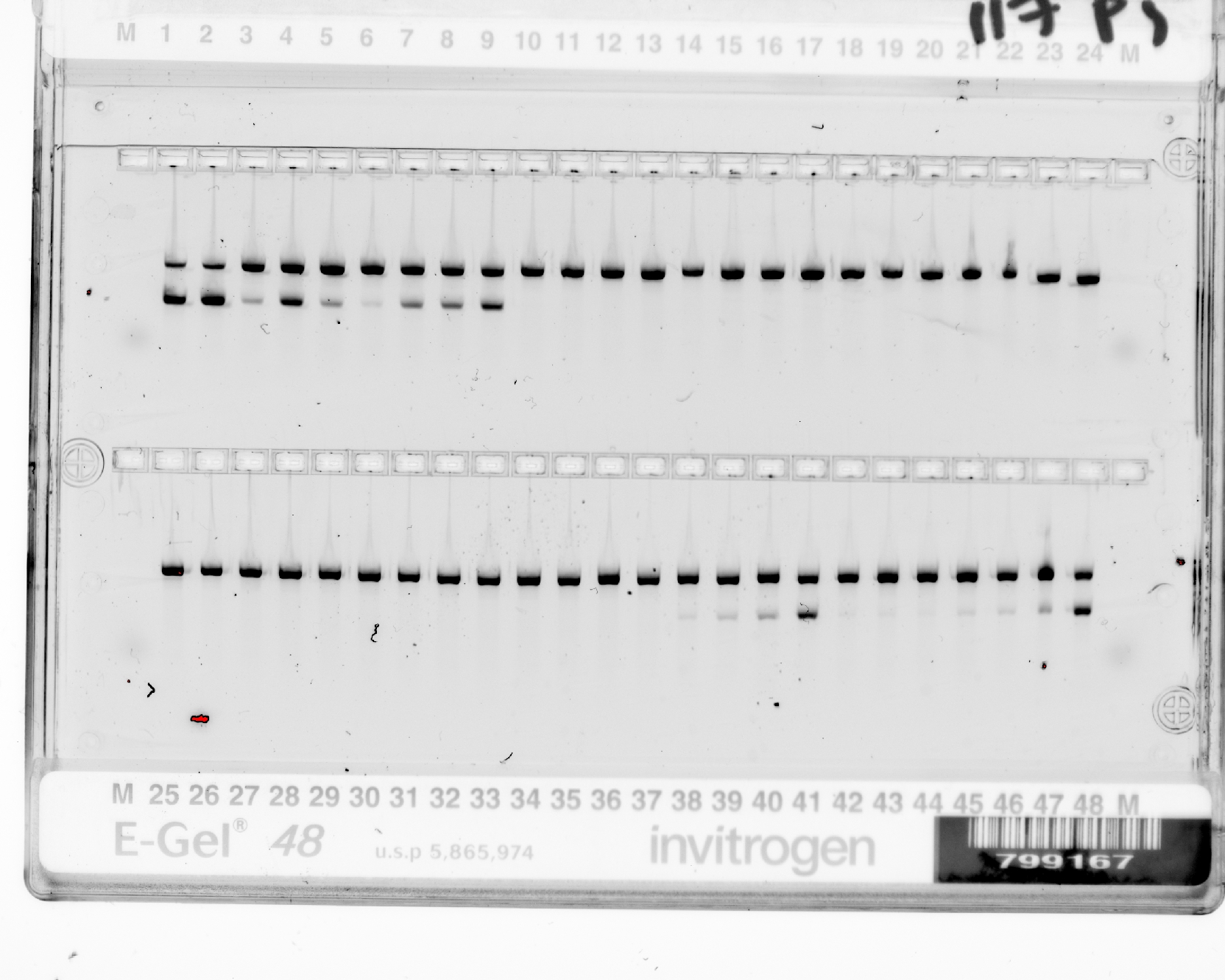

Supplement: Supplementary file 11 — Uncropped gels. [file 41587_2025_2655_MOESM11_ESM.zip › Source Data Fig 4/Fig4B_EDFig9A_Cy5_1.jpg]

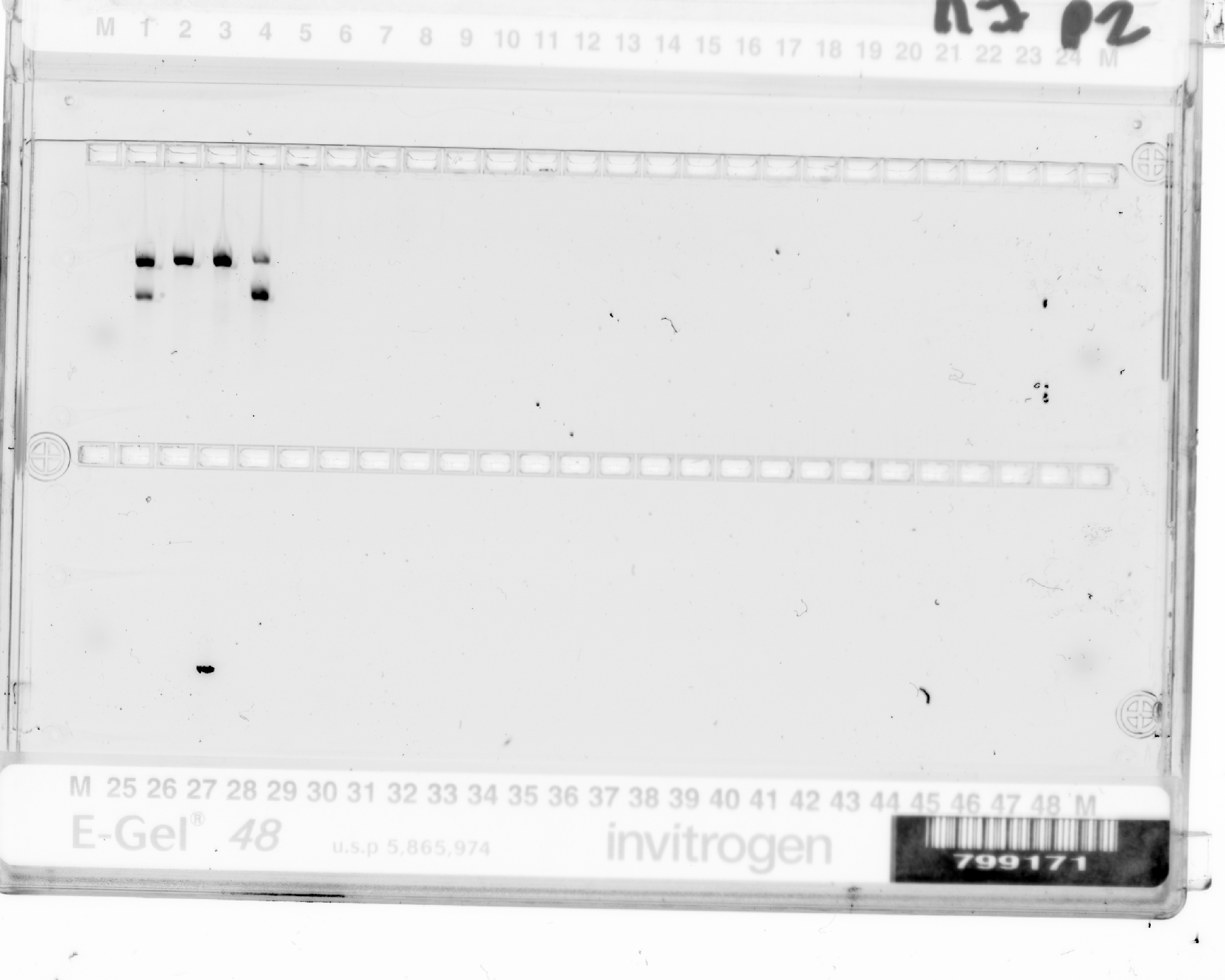

Supplement: Supplementary file 11 — Uncropped gels. [file 41587_2025_2655_MOESM11_ESM.zip › Source Data Fig 4/Fig4B_EDFig9A_Cy5_2.jpg]

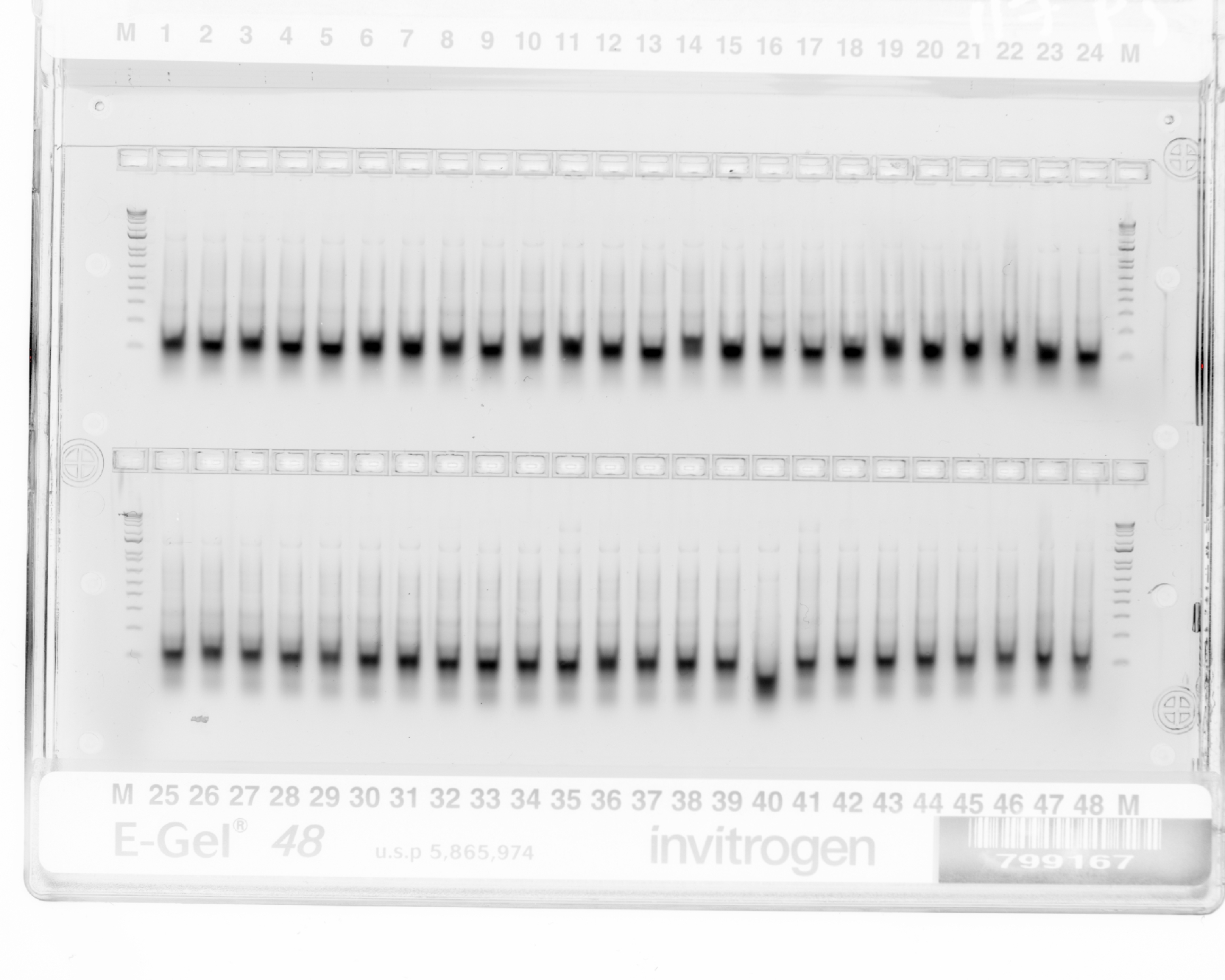

Supplement: Supplementary file 11 — Uncropped gels. [file 41587_2025_2655_MOESM11_ESM.zip › Source Data Fig 4/Fig4B_EDFig9A_SYBRGold_1.jpg]

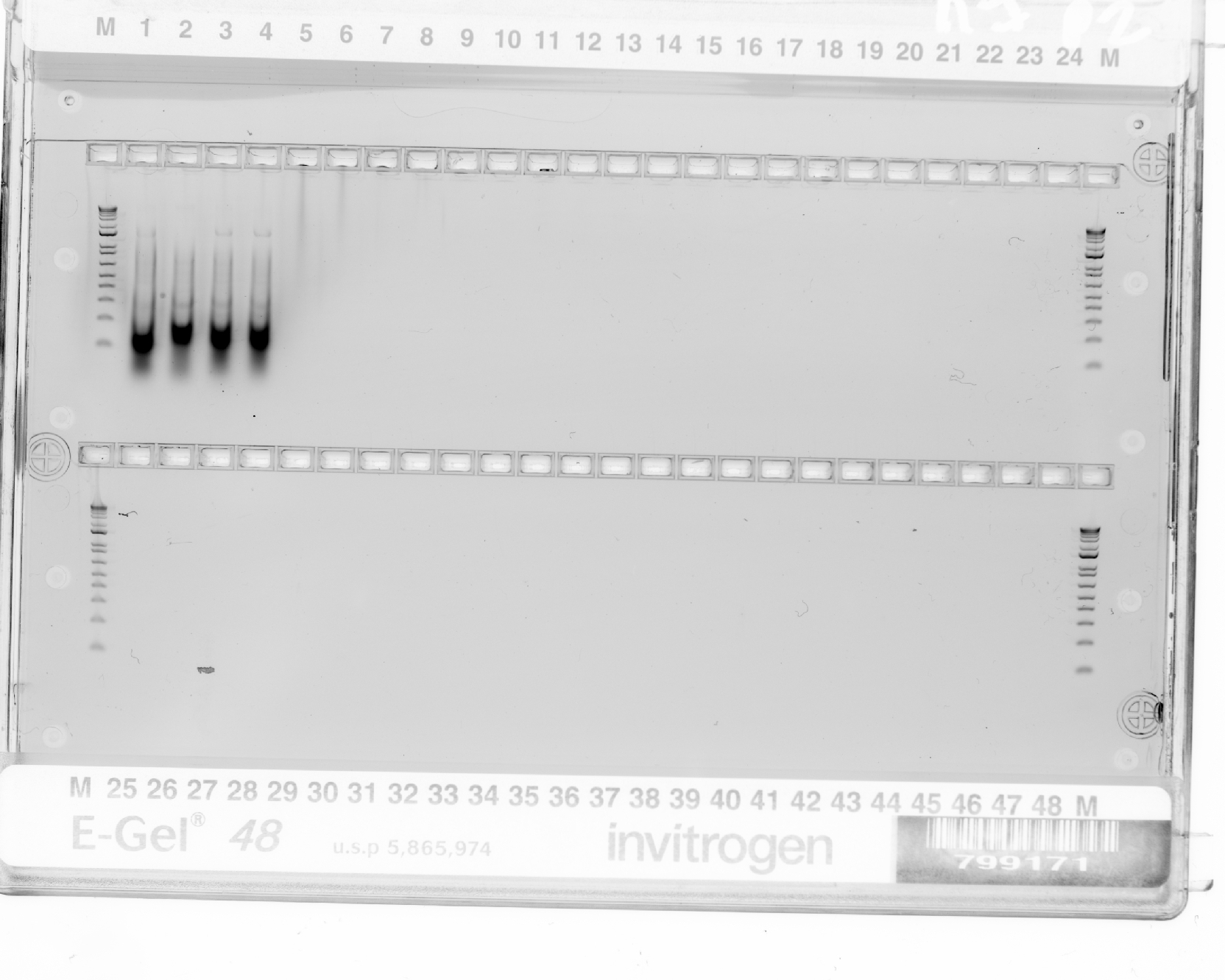

Supplement: Supplementary file 11 — Uncropped gels. [file 41587_2025_2655_MOESM11_ESM.zip › Source Data Fig 4/Fig4B_EDFig9A_SYBRGold_2.jpg]

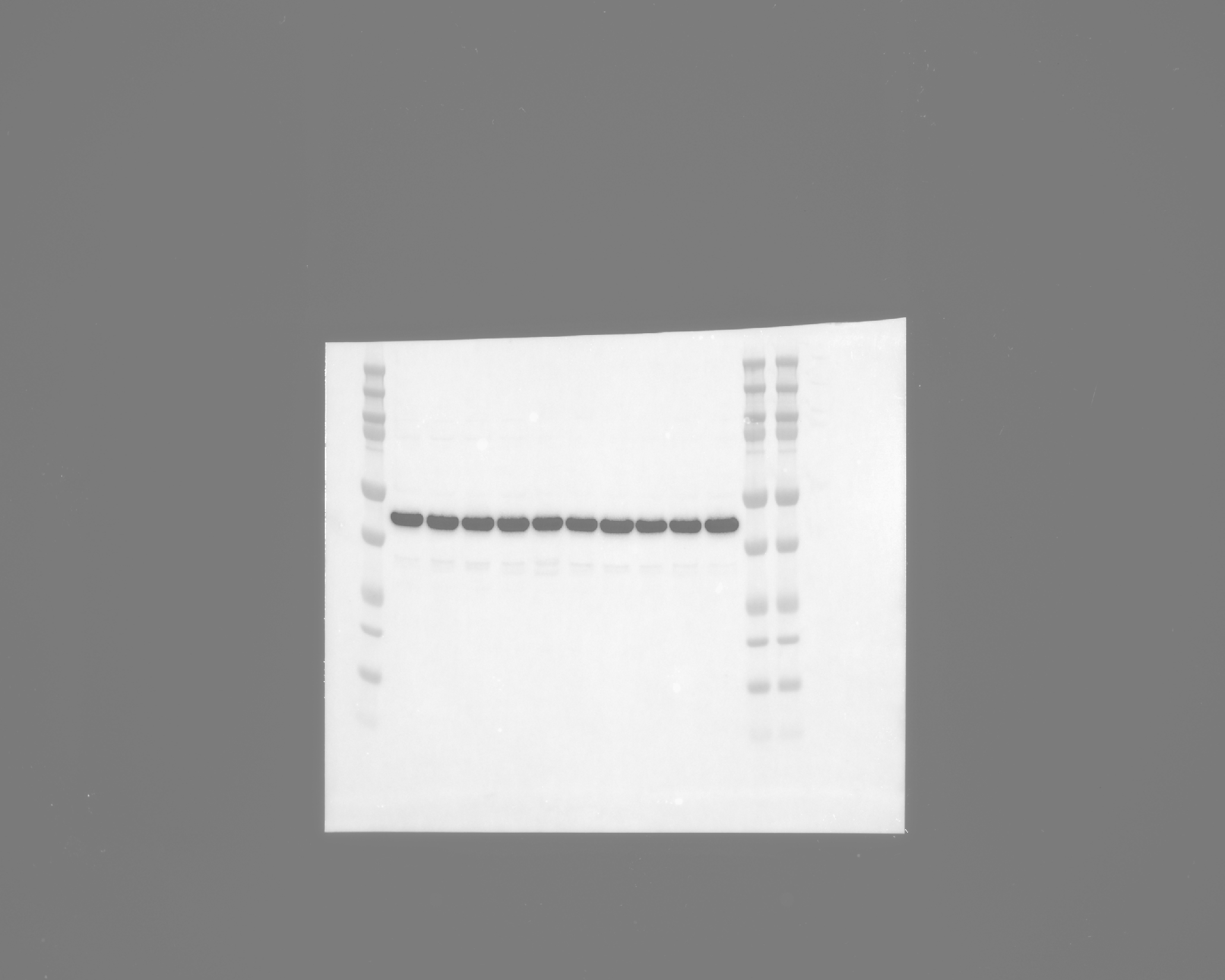

Supplement: Supplementary file 12 — Uncropped gels. [file 41587_2025_2655_MOESM12_ESM.zip › Source Data Fig 5/Figure_5G_beta_actin.jpg]

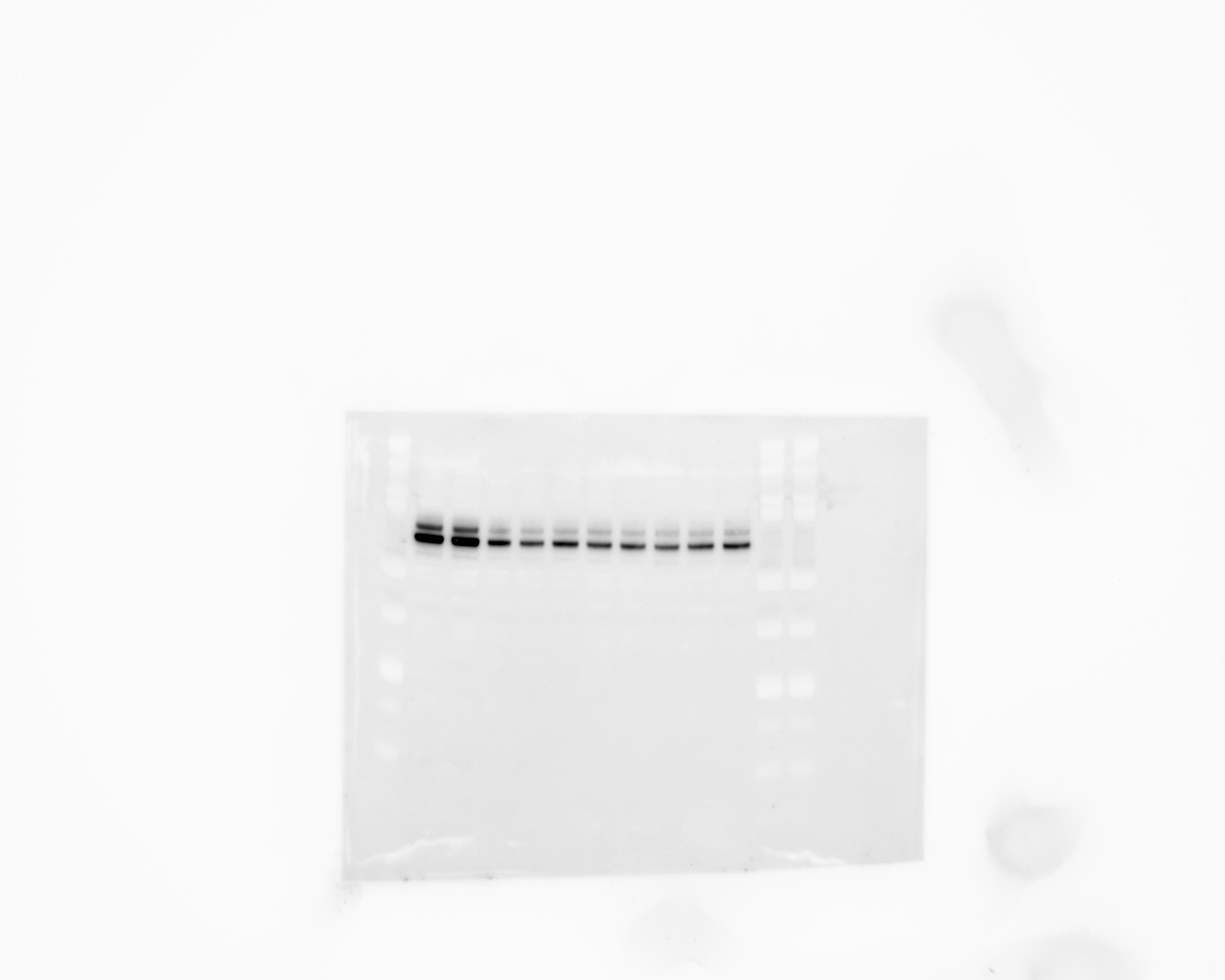

Supplement: Supplementary file 12 — Uncropped gels. [file 41587_2025_2655_MOESM12_ESM.zip › Source Data Fig 5/Figure_5G_Pcsk9.jpg]

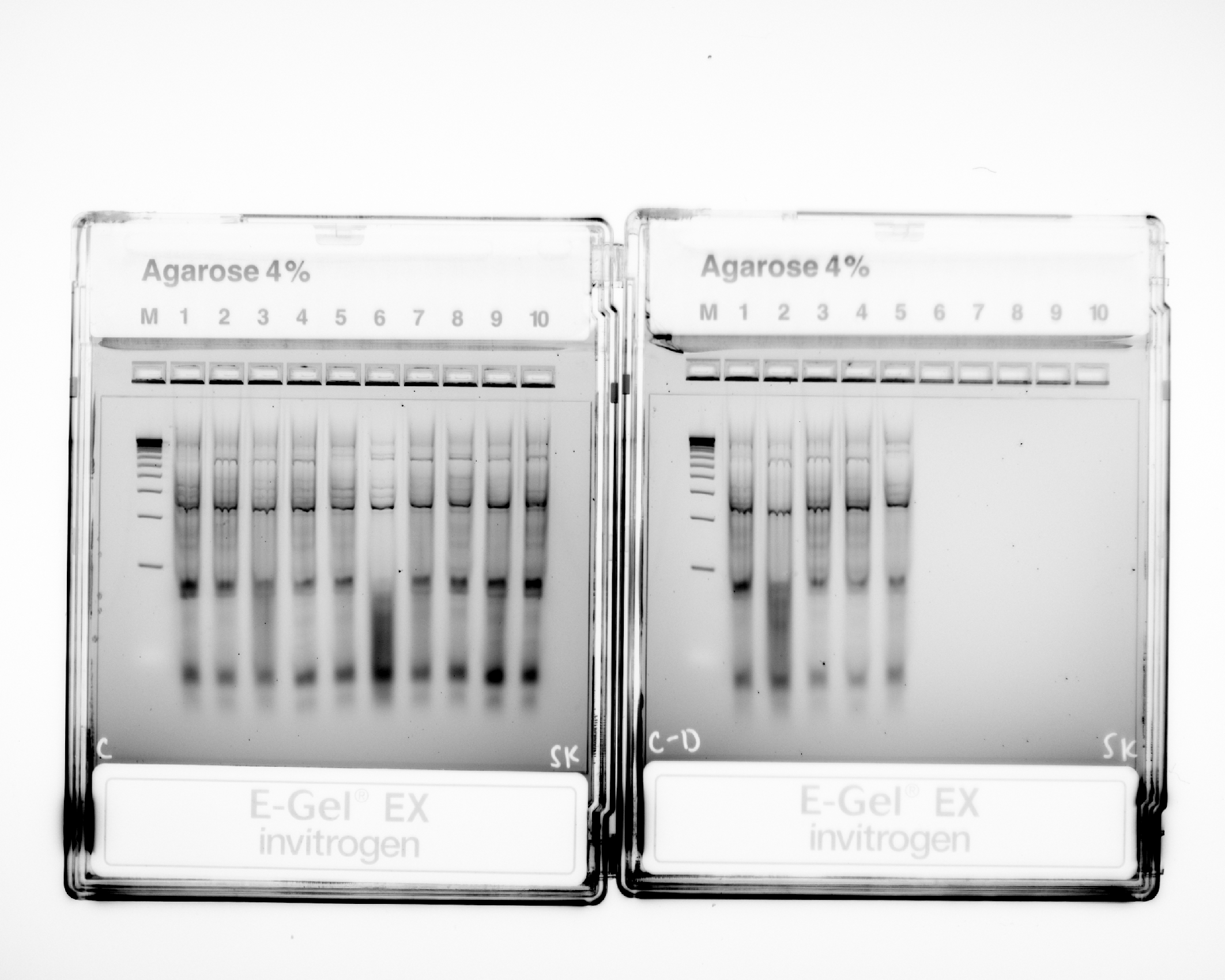

Supplement: Supplementary file 13 — Uncropped gels. [file 41587_2025_2655_MOESM13_ESM.zip › Source Data Extended Data Fig 4/EDFig4A_Cy3.tif]

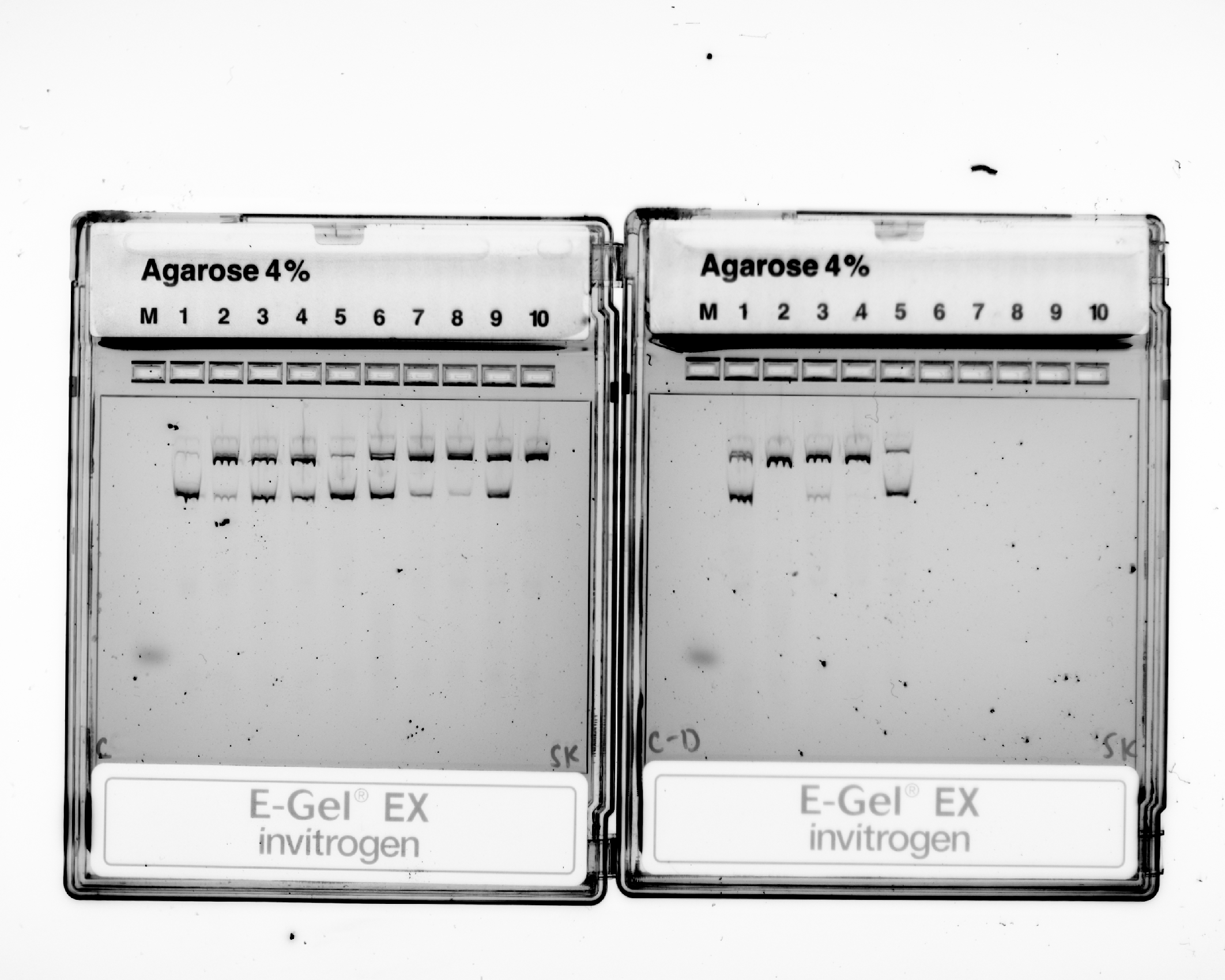

Supplement: Supplementary file 13 — Uncropped gels. [file 41587_2025_2655_MOESM13_ESM.zip › Source Data Extended Data Fig 4/EDFig4A_Cy5.tif]

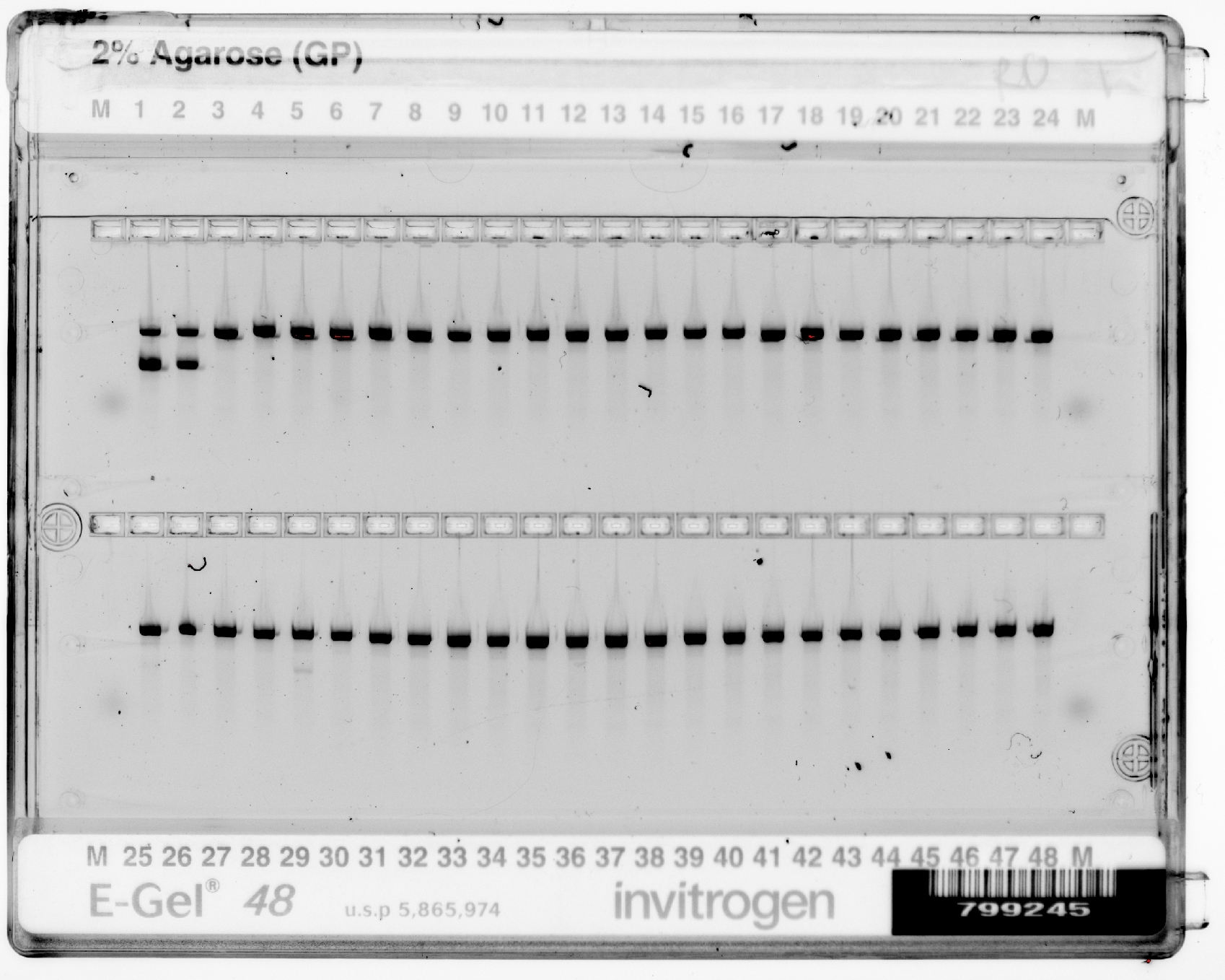

Supplement: Supplementary file 13 — Uncropped gels. [file 41587_2025_2655_MOESM13_ESM.zip › Source Data Extended Data Fig 4/EDFig4C_Cy5.jpg]

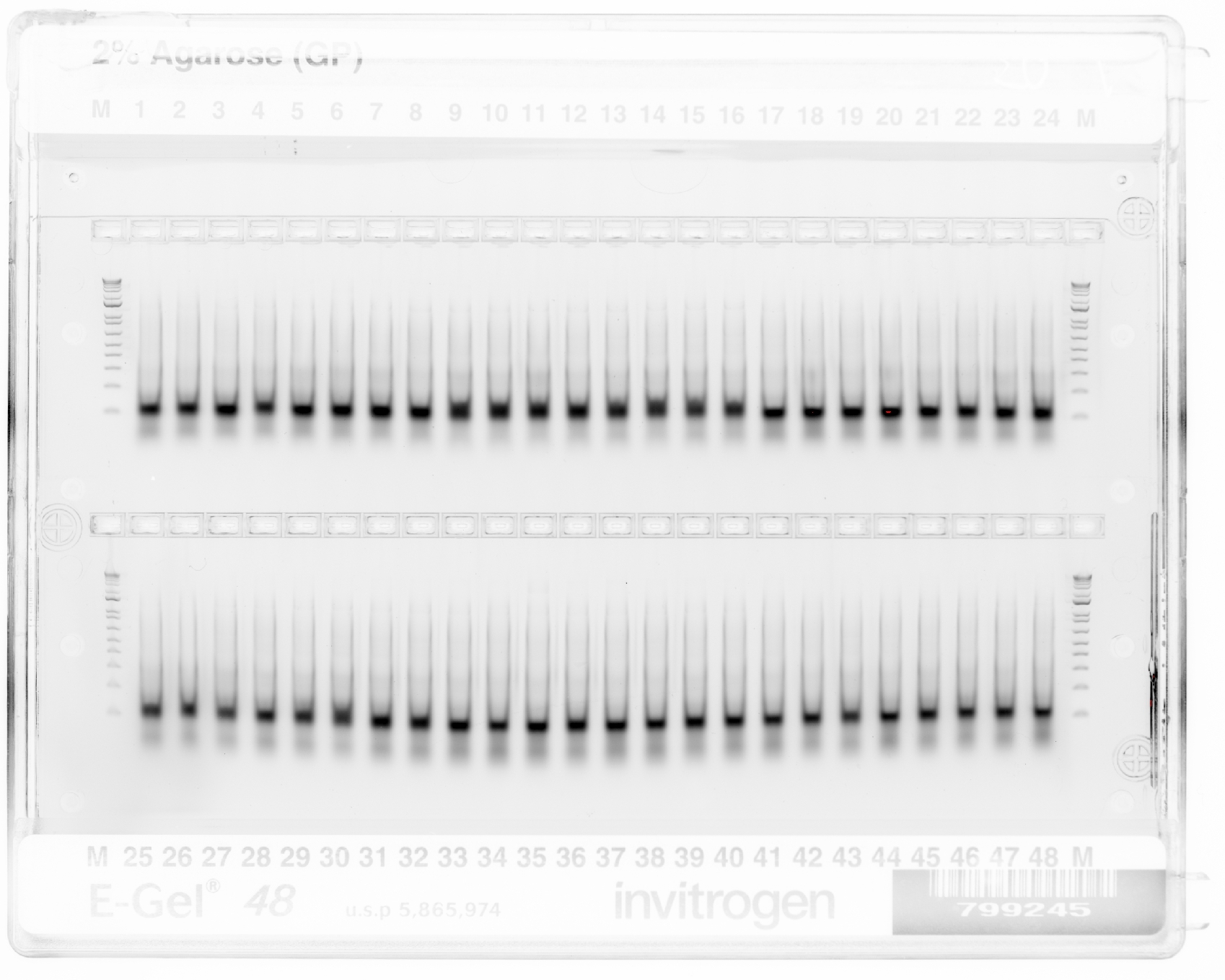

Supplement: Supplementary file 13 — Uncropped gels. [file 41587_2025_2655_MOESM13_ESM.zip › Source Data Extended Data Fig 4/EDFig4C_EtBr.jpg]

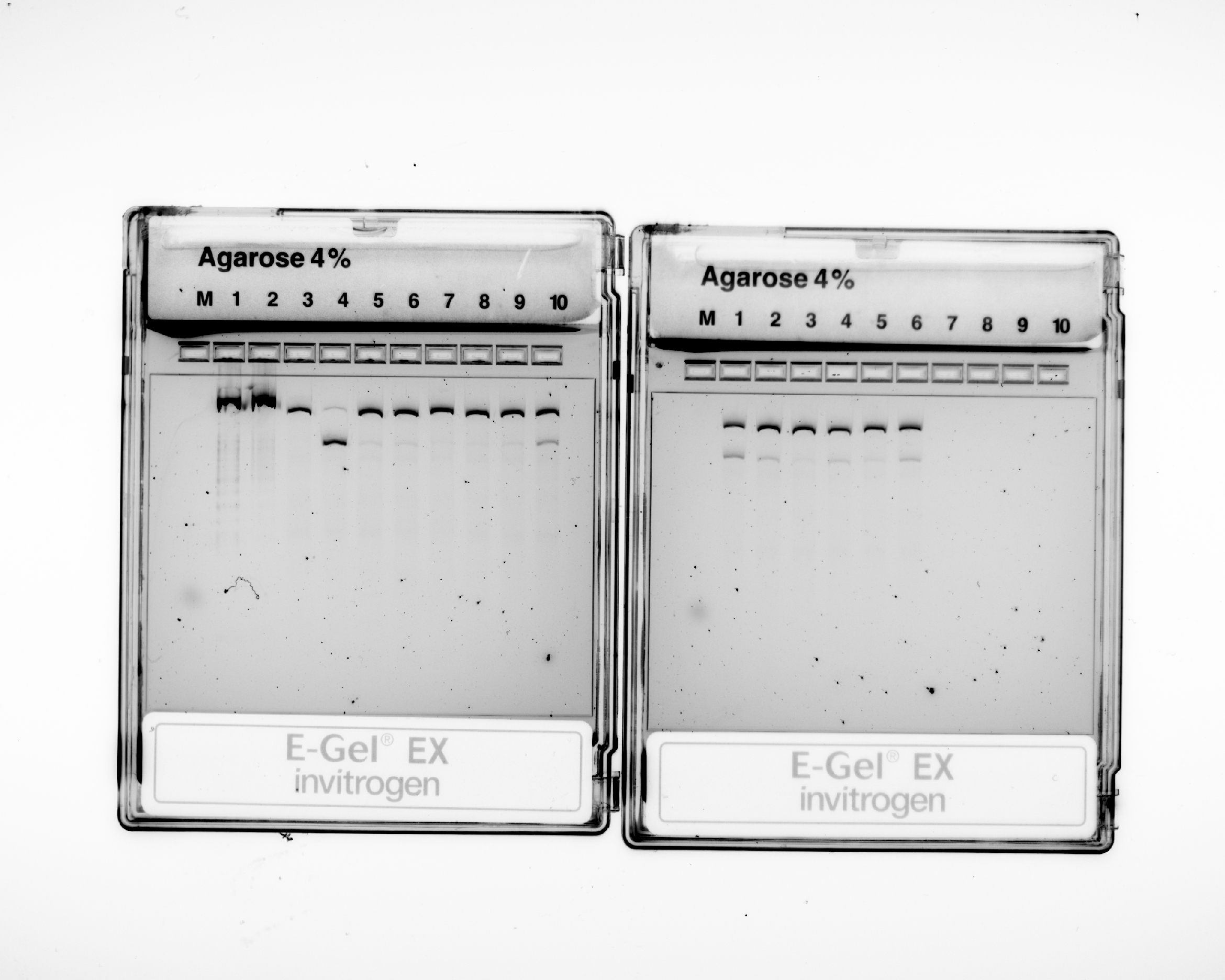

Supplement: Supplementary file 14 — Uncropped gels. [file 41587_2025_2655_MOESM14_ESM.zip › Source Data Extended Data Fig 6/EDFig6A.jpg]

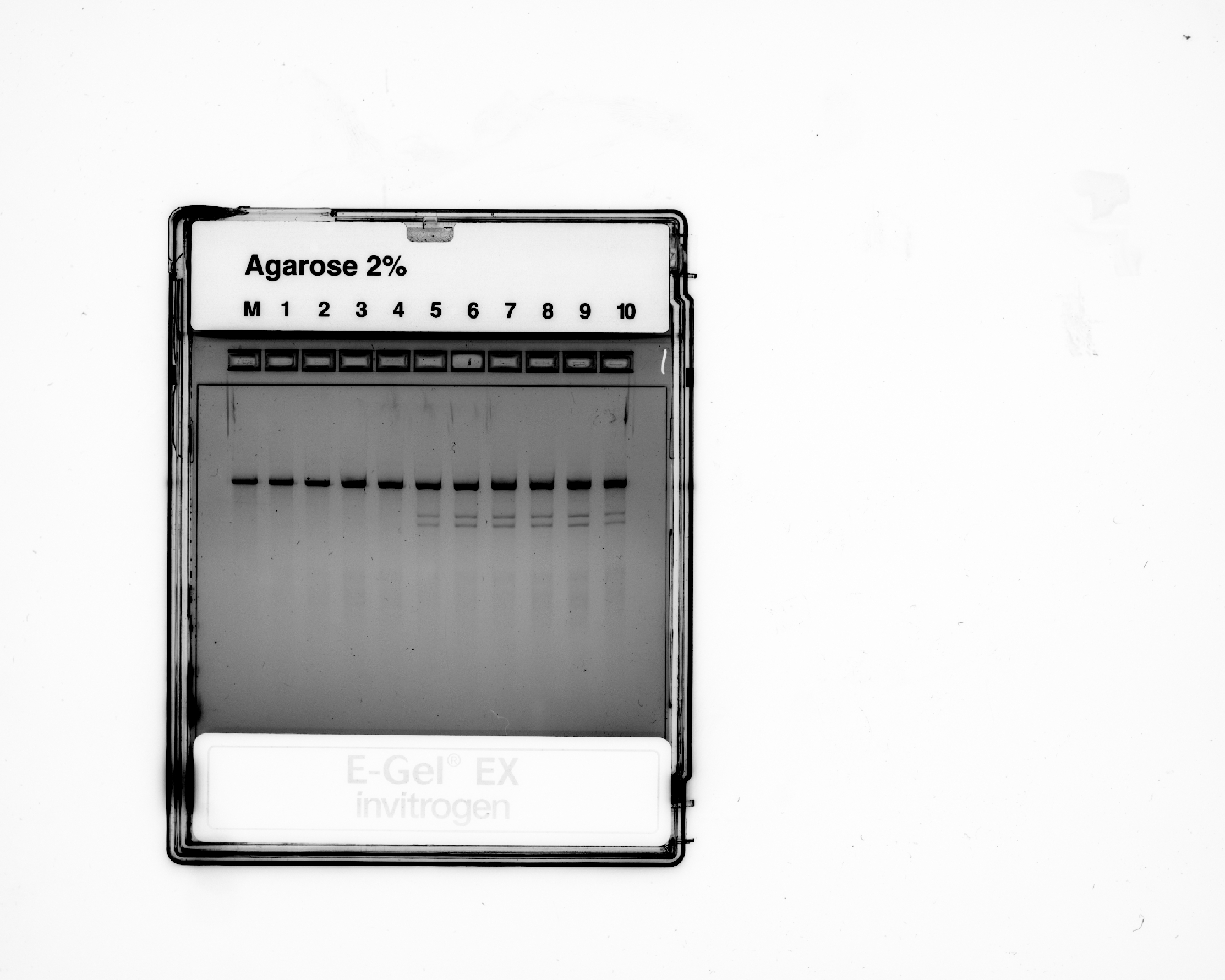

Supplement: Supplementary file 14 — Uncropped gels. [file 41587_2025_2655_MOESM14_ESM.zip › Source Data Extended Data Fig 6/EDFig6E.jpg]
